# Controlling the spatial distribution of electronic excitation in asymmetric D–A–D′ and symmetric D′–A–D–A–D′ electron donor–acceptor molecules†

**DOI:** 10.1039/d5sc01257k

**Published:** 2025-04-04

**Authors:** Evangelos Balanikas, Tommaso Bianconi, Pietro Mancini, Nikhil Ji Tiwari, Manju Sheokand, Rajneesh Misra, Benedetta Carlotti, Eric Vauthey

**Affiliations:** a Department of Physical Chemistry, University of Geneva 30 Quai Ernest-Ansermet CH-1211 Geneva 4 Switzerland eric.vauthey@unige.ch; b Department of Chemistry, Biology and Biotechnology, University of Perugia via elce di sotto 8 06123 Perugia Italy benedetta.carlotti@unipg.it; c Department of Chemistry, Indian Institute of Technology Indore 453552 India rajneeshmisra@iiti.ac.in

## Abstract

Understanding how electronic energy is funnelled towards a specific location in a large conjugated molecule is of primary importance for the development of a site-specific photochemistry. To this end, we investigate here how electronic excitation redistributes spatially in a series of electron donor–acceptor (D–A) molecules containing two different donors, D and D′, and organised in both linear D–A–D′ and symmetric double-branch D′–A–D–A–D′ geometries. Using transient IR absorption spectroscopy to probe the alkyne spacers, we show that for both types of systems in non-polar solvents, excitation remains delocalised over the whole molecule. In polar media, charge-transfer (CT) exciton in the linear D–A–D′ systems localises rapidly at the end with the strongest donor. For the double-branch systems, excited-state symmetry breaking occurs and the CT exciton localises at the end of one of the two branches, even if the D′ terminal donor is not the strongest one. This unexpected behaviour is explained by considering that the energy of a CT state depends not only on the electron donating and withdrawing properties of the donor and acceptor constituents, but also on the solvation energy. This study demonstrates the possibility to control the location of CT excitons in large conjugated systems by varying the nature of the donors and acceptors, the distance between them as well as the environment.

## Abstract 2

Using ultrafast time-resolved IR spectroscopy, we find that charge-transfer excitation does not always localise on the strongest electron donor–acceptor pair. Solvation is another crucial factor which determines where the exciton is trapped.

## Graphical abstract

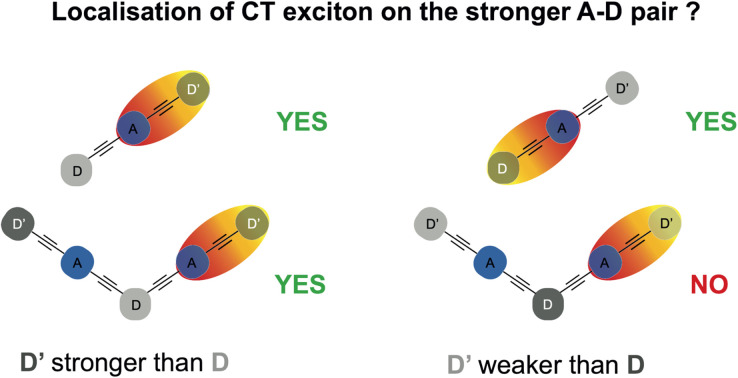

## 1 Introduction

Over the past few years, there has been a growing activity in the investigation of multipolar conjugated molecules consisting of a central electron withdrawing group (A) with two or more identical electron donors (D), or *vice versa*. Molecules with such design were shown to have several advantageous properties, such as a large two-photon absorption cross section,^1–3^ that make them particularly attractive for a broad range of applications, including two-photon fluorescence imaging,^4–6^ two-photon induced polymerisation,^7–9^ organic photovoltaics,^10–12^ OLEDs,^13,14^ and sensing.^15,16^ A puzzling peculiarity of these molecules is their unexpectedly large fluorescence solvatochromism.^17–24^ Its origin was shown to arise from the strong solvent dependence of the spatial distribution of their lowest singlet exciton.^18,25–34^ Indeed, in non-polar media, excitation is distributed evenly over the whole molecule, whereas as the polarity of the solvent increases, it localises more and more on a single D–A branch, resulting in a so-called excited-state symmetry breaking (ESSB) and a dipolar excited state.^33,35–40^ According to a simple excitonic picture,^41^ where each D–A branch is considered as a charge-transfer (CT) chromophore, the delocalised excited state is energetically stabilised relatively to the localised state through the interbranch excitonic coupling, equivalent to half the Davidov splitting. On the other hand, localised excitation results in a dipolar state, hence to an increased solvation energy in polar media. Therefore, occurrence of ESSB depends on the balance between the loss of excitonic stabilisation and the gain of solvation energy.^42–45^ ESSB was shown to have a significant relevance to the photochemical reactivity of large conjugated systems, as it leads to substantial changes of electronic density in a limited area of the molecule.^46^ Therefore, a proper control of the region whereto electronic energy is funnelled would be an important step towards an ‘addressable site-specific’ photochemistry of large conjugated systems. A first step in this direction was recently realised with a series of triads consisting of a central quadrupolar core and two lateral diphenylethynyl branches.^47^ Depending on the end substituents on the core and the lateral branches, three different types of ESSB processes leading to distinct locations of the CT exciton could be evidenced.

**Fig. 1:**
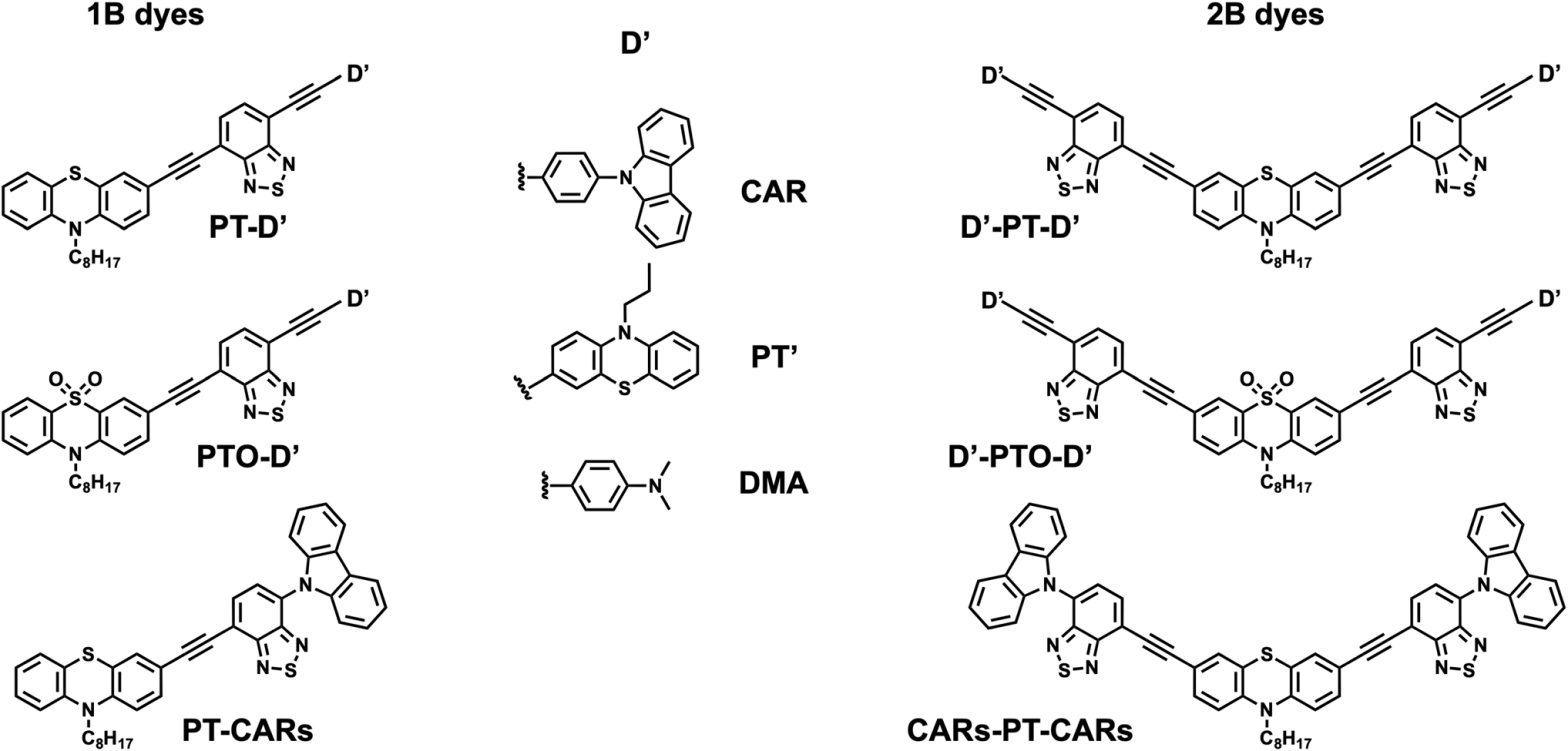
Structures of the single-branch (1B) and double-branch (2B) dyes. (The ‘s’ of CARs stands for single bond.)

Here, we go a step further and investigate how and where electronic excitation localises in a series of asymmetric D–A–D′ and symmetric D′–A–D–A–D′ dyes with alkyne spacers in solution. As illustrated in Fig. 1, the electron withdrawing group, A, is a benzothiadiazole (BTD) unit and is common to all molecules. First, we keep the same D sub-unit constant, *i.e.* a phenothiazine (PT), and vary the donating strength of D′, starting with a weaker donor, carbazole (CAR, *E*_ox_ = 1.15 V *vs.* SCE),^48^ continuing with a similar phenothiazine donor, PT′ (*E*_ox_ = 1.05 V *vs.* SCE),^49^ and a stronger donor, *N*,*N*-dimethylaniline (DMA, *E*_ox_ = 0.7 V *vs.* SCE).^50^ Finally, we increase further the difference in donor strength between D and D′ by replacing D = PT by a phenothiazine 5,5-dioxide (PTO, *E*_ox_ ∼1.4 V *vs.* SCE)^51^ and keeping D′ = DMA. A previous investigation with some of these dyes, namely, CARs-PT-CARs and CAR-PT-CAR, revealed a significant CT character of their excited state.^52^

To probe the location of the electronic excitation, we apply time-resolved IR absorption (TRIR) spectroscopy using the –C≡C– stretching modes of the alkyne spacers as vibrational markers. This was shown to be a powerful approach to detect ESSB in centrosymmetric multibranched D–A molecules,^33,53^ and to determine the location of the electronic excitation in conjugated D–A dyes.^47^ To look at the effect of the donor–acceptor distance, we also investigate a D′–A–D and D′–A–D–A–D′ couple with a single bond between A and D′ (PT-CARs and CARs-PT-CARs). Additionally, this reduces the number of alkyne spacers and simplifies the interpretation of the TRIR data.

We show that the distribution of the electronic excitation varies from delocalised over the whole D′–A–D–A–D′ molecule to localised on a single A–D′ end, depending on the difference of donating strength between D and D′ as well as the solvent polarity and the donor–acceptor distance. These results represent a further step towards the control of the position of CT excitons in large conjugated systems.

## 2 Results

### 2.1 Synthesis

The synthesis of CARs-PT-CARs and CAR-PT-CAR was described in ref. 52. The other compounds were synthesised using a similar approach as described in detail in the ESI.† All dyes were purified by column chromatography and further characterized using NMR and HRMS techniques.

### 2.2 Stationary spectroscopy

**Fig. 2:**
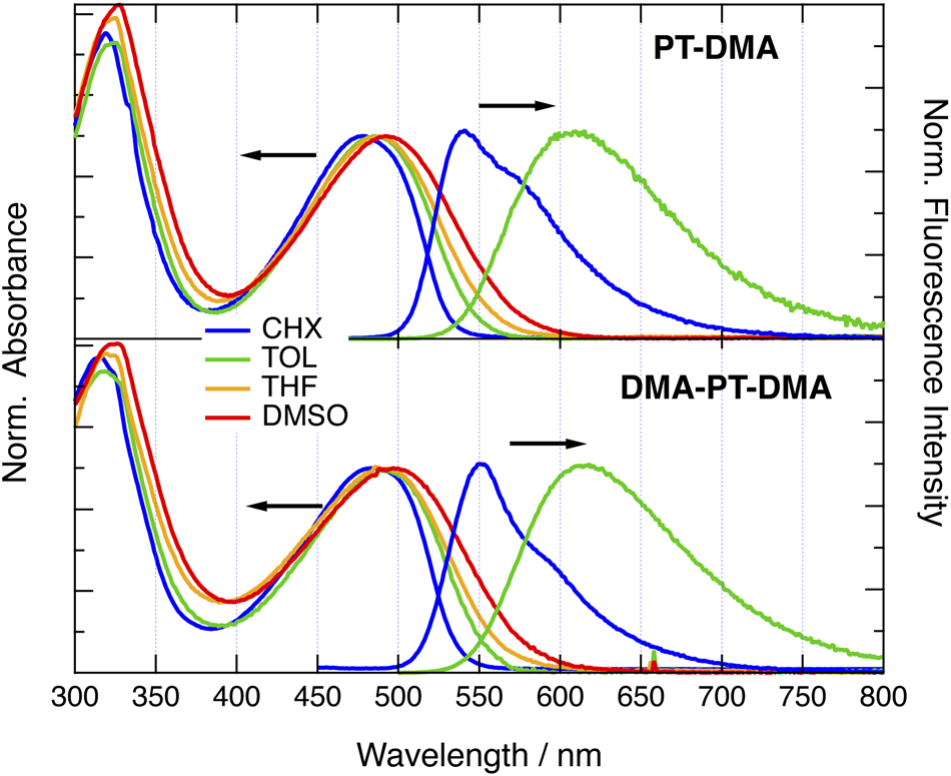
Stationary electronic absorption and fluorescence spectra of PT-DMA and DMA-PT-DMA in various solvents. CHX: cyclohexane; TOL: toluene; THF: tetrahydrofuran; DMSO: dimethylsulfoxide.

All dyes with a PT donor exhibit similar absorption spectra, with a broad structureless band peaking in the 450–500 nm region and a more intense band in the 300–350 nm range (Fig. 2 and S1–S5†). With PTO donor, the low-energy band is slightly up-shifted relatively to the other dyes and the higher energy region exhibits additional spectral features that could be due to a vibronic structure.^52,54^ No evident relationship between the nature of the end donor, D′, and the position of the lower-energy band could be identified. However, at constant D and D′, the lower-energy band of the two-branched (2B) dyes is systematically red-shifted by a few hundreds cm^−1^ relative to the one-branched (1B) analogue. The dyes exhibit only weak absorption solvatochrochromism, indicative of a small permanent dipole moment in the ground state.

All dyes are highly fluorescent with quantum yields above 0.15 in the non-polar cyclohexane (CHX) and the weakly-polar toluene (TOL) (Table S1†).^52^ However, emission decreases below the limit of detection in polar solvents. The fluorescence exhibits strong solvatochromism with spectral shifts of more than 2000 cm^−1^ when going from CHX to TOL (Fig. 2 and S1–S5†). For the 2B dyes soluble in CHX, this solvent shift is smaller by a few hundreds of cm^−1^ compared to their 1B analogues. Similarly to the absorption, the emission maximum of the 2B dyes in CHX is red shifted by 200–300 cm^−1^ relative to that of the 1B dyes. The fluorescence lifetime in these two solvents ranges from 2 to 5 ns (Table S1†).

### 2.3 Quantum-chemistry calculations

**Fig. 3:**
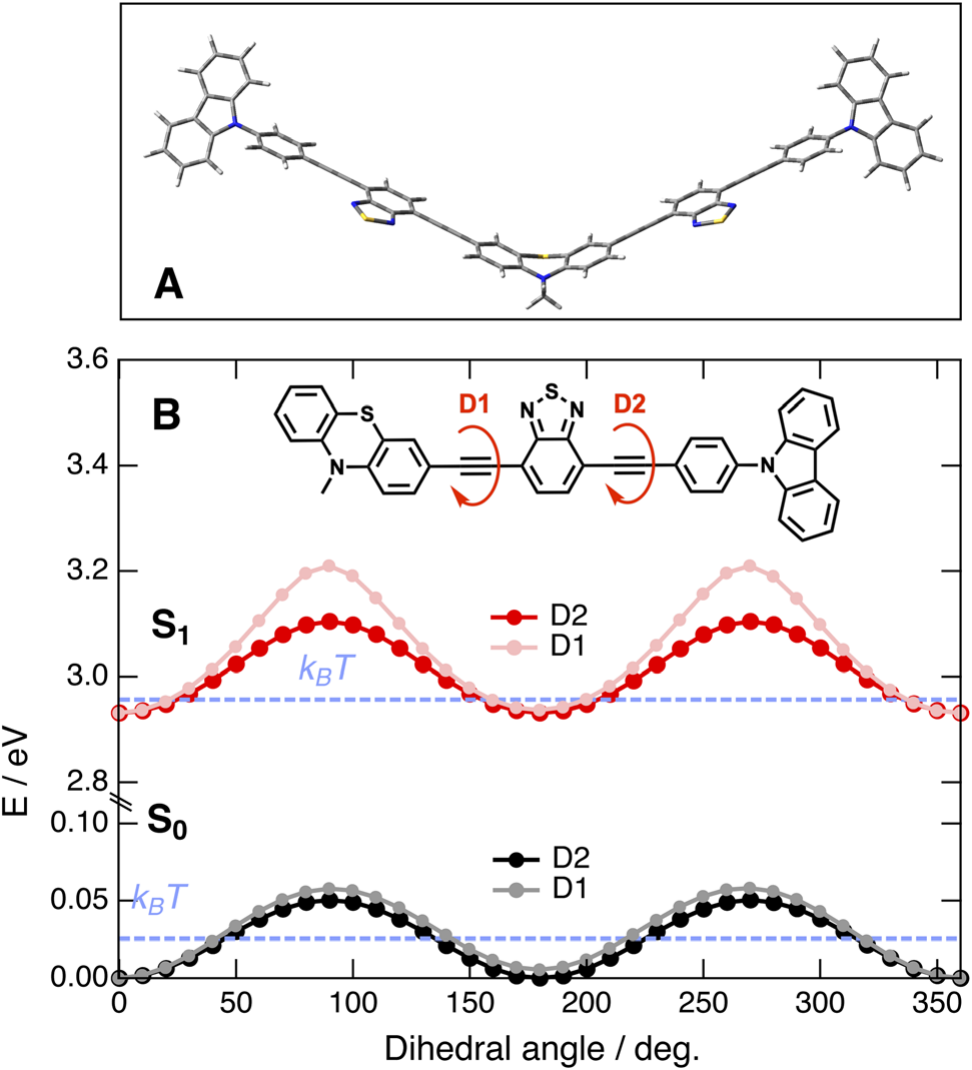
(A) Optimised ground-state geometry of CAR-PT-CAR. (B) Energy of PT-CAR in the ground and Franck–Condon S_1_ states as a function of the dihedral angles D1 and D2 obtained from TD-DFT calculations. For efficiency, the octyl substituent on the PT nitrogen was replaced by a methyl group.

Quantum-chemical calculations at the density functional theory (DFT) and time-dependent (TD) DFT levels (CAM-B3LYP/6-31g(d,p)) were performed to obtain further insight into the nature of the lowest energy electronic transitions of these dyes. As illustrated in Fig. 3A with the optimised ground-state geometry of CAR-PT-CAR, the 2B dyes have a V shape due to the non-planar geometry of the central PT.^55^ Whereas the carbazole unit makes an angle of about 50° with the adjacent phenyl, the latter lies in the same plane as the A and D moieties. However, relaxed scan calculations with PT-CAR along the dihedral angles between the PT and BTD planes and between the BTD and the phenyl planes point to a distribution of torsional angle around the two ethynyl bonds at room temperature (Fig. 3B), with similar disorder for both dihedral angles. Such torsional flexibility is characteristic of molecules with ethynyl spacers.^56–60^ These calculations also predict the existence of two conformers of the 1B dyes, depending on whether the BTD moiety has the S atom on the same side (*cis*) as the S atom of the PT sub-unit or on the opposite side (*trans*) as drawn in Fig. 1. For the 2B dyes, this results in four conformers. According to the calculations with PT-CAR, the geometry with the S atoms in trans configuration is more stable by 5.6 meV. Therefore, these two and four conformers of the 1B and 2B dyes should be equally present at room temperature. For CARs-PT-CARs, the dihedral angle between the planes of the BTD and CAR groups amounts to about 60° due to the steric hindrance associated with the single bond connecting these two moieties (Fig. S7†).

**Fig. 4:**
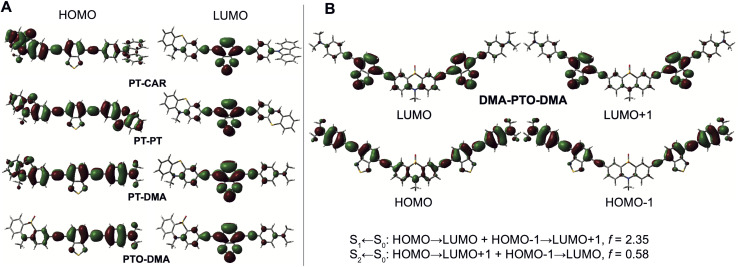
(A) Frontier molecular orbitals of the single-branch dyes illustrating the influence of the relative strength of the two donors, D and D′, on the nature of the CT excited state. (B) Frontier molecular orbitals involved in the first two lower-energy transitions of DMA-PTO-DMA and corresponding oscillator strength (*f*).

TD-DFT calculations with the 1B molecules predict the lowest-energy transition to be dominated by a one-electron HOMO–LUMO transition. The LUMO is always centred on the BTD acceptor, whereas the location of the HOMO depends on the relative electron-donating strength of D and D′ (Fig. 4A). For D = PT, the HOMO has higher amplitude on PT for PT-CAR, on DMA for PT-DMA and equal amplitude for PT-PT′. Finally, with PTO-DMA, the HOMO is mostly centred on the DMA end. According to these calculations, the absorption band measured in the 300–350 nm region is due to higher-energy transitions localised on the PT and BTD moities. This agrees well with the absorption spectra reported for these two molecules in literature.^61,62^

TD-DFT calculations with PT-CAR along the PT–BTD and BTD-CAR torsional coordinates predict barriers for torsion larger by a factor of more than 3 in the Franck–Condon S_1_ state than in the ground state (Fig. 3B). As a consequence, the S_1_–S_0_ gap also depends on these angles. Therefore, the torsional disorder existing in the ground state at room temperature should result in an inhomogeneous broadening of the S_1_ ← S_0_ band, with the red-edge due the absorption of the most planar dyes.^56,59,60^ Moreover, the transition energies and oscillator strengths for the two planar conformers with the S atoms in trans or cis configurations, are essentially the same (Fig. 3). Geometry optimisation of the S_1_ state of PT-CAR, also predict a planar equilibrium geometry with essentially the same energy for both conformers. Therefore, both trans and cis conformers have the same probability to be photoexcited. Whereas calculations point to significant changes in the frequencies of the –C≡C– stretching vibrations upon S_1_ ← S_0_ excitation, negligibly small differences are predicted between the trans and cis conformers (Table S2†).

The frontier molecular orbitals (MOs) of the 2B dyes are similar to those of the 1B dyes, with each 1B MO split into two MOs with gerade and ungerade symmetry (Fig. 4B and S8–S10†). The lowest-energy transition corresponds to a combination of HOMO–LUMO and HOMO−1 to LUMO+1 one-electron transitions and is characterised by a large oscillator strength (*f* > 1), whereas the next one is associated with HOMO–LUMO+1 and HOMO−1 to LUMO transitions and exhibits a smaller oscillator strength (*f* < 0.6) (Fig. 4B and S8–S10†). These calculations suggest symmetric electronic structures of the 2B dyes in the ground state as well as in the Franck–Condon S_1_ and S_2_ states, and substantial changes in quadrupolar moment upon S_1_ ← S_0_ and S_2_ ← S_0_ excitation. This behaviour is consistent with the above-mentioned excitonic model, where each branch is a CT chromophore and the lowest and highest excitonic states are associated with the additive and subtractive combinations of the transition dipole moments of each branch. Unlike centrosymmetric linear multibranched D–A dyes, the transition to the higher excitonic state of the 2B dyes is not symmetry forbidden for one-photon absorption, because the transition dipoles of each branch do not cancel completely.

The energy gap between the S_2_ and S_1_ states corresponds to the Davidov splitting and, thus, to twice the interbranch excitonic coupling, *V*_ib_.^42,45^ According to the S_2_–S_1_ splitting obtained from the TD-DFT calculations, *V*_ib_ should be within the 450–600 cm^−1^ range (Table S3†). Because of the small oscillator strength, the energy of the S_2_ ← S_0_ transition cannot be extracted from the one-photon absorption spectra of the 2B dyes. However, this information can be obtained from the two-photon absorption spectra of CARs-PT-CARs and CAR-PT-CAR reported in ref. 52. These data point to a *V*_ib_ of about 250 and 400 cm^−1^, respectively, in agreement with the calculations.

### 2.4 Electronic transient absorption spectroscopy

**Fig. 5:**
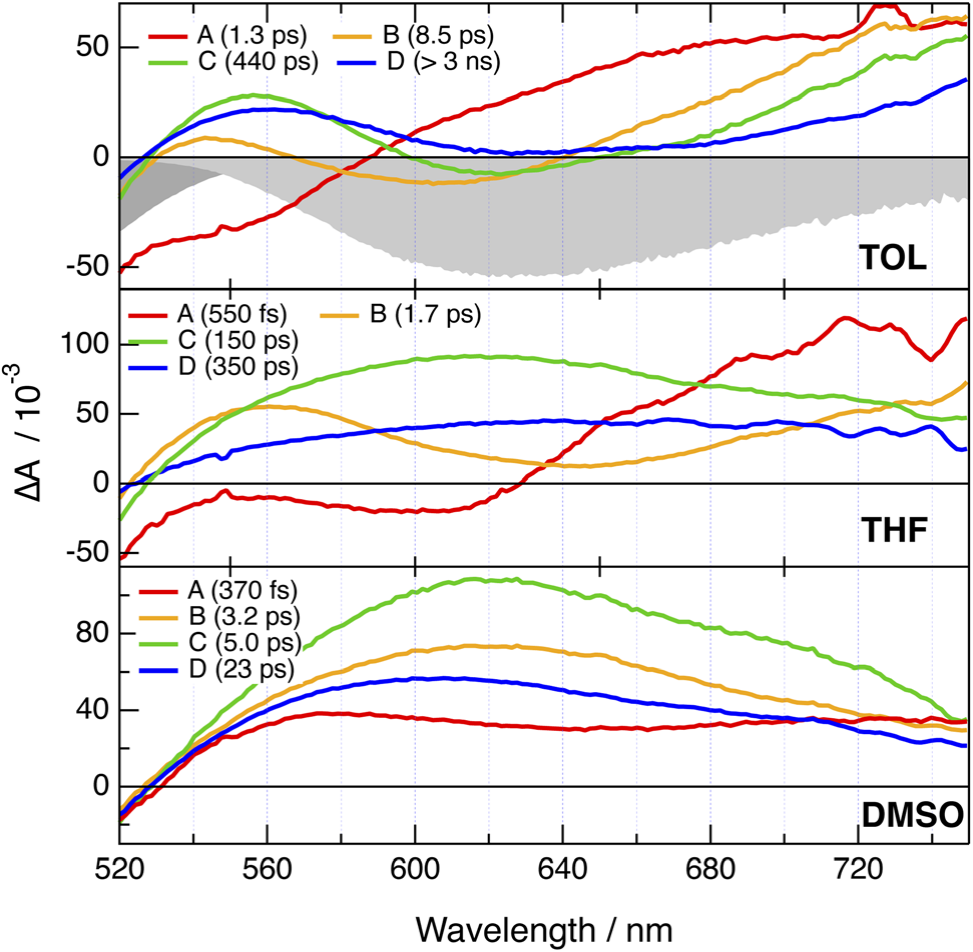
Evolution-associated difference absorption spectra and time constants obtained from a global analysis of the transient absorption data measured with PT-DMA in various solvents, assuming a series of four successive exponential steps. The negative stationary absorption and stimulated emission spectra in TOL are shown in grey.

Electronic transient absorption (TA) measurements were performed with PT-PT′, PT-DMA and PTO-DMA and their 2B analogues in TOL, THF and DMSO (Fig. 5 and S11–S21†), whereas TA spectra measured with CARs-PT-CARs and CAR-PT-CAR were reported previously.^52^ Negligible difference between the 1B and 2B dyes can be observed, as testified by the evolution-associated difference absorption spectra (EADS) obtained from a global analysis assuming a series of successive exponential steps (Fig. 5 and S11–S21†). These EADS do not correspond to well-defined states or species but allow for a better visualisation of the spectral dynamics and their timescales.^63,64^

In all cases, the TA spectra are dominated by a broad positive band starting above 520 nm and extending beyond 700 nm. In TOL, a negative feature that can be assigned to stimulated emission (SE) is visible at early time below 600 nm. This band shifts to longer wavelengths and loses intensity during the first few tens of ps. In THF, SE is only visible in the earliest spectra, and probably shifts outside the spectral window and/or loses too much intensity to be visible after a few ps. In DMSO, the SE can only be distinguished during the first few hundreds fs as a dip centred around 640 nm and overlapping with the broad positive excited-state absorption. These spectral dynamics can be attributed to the dynamic Stokes shift of the SE due to solvent relaxation.^65^ Once this shift of the SE band over, the TA spectra show little change apart from a decay of the amplitude occurring on a timescale decreasing from a few ns to a few tens of ps when going from TOL to DMSO. This is consistent with the ns fluorescence lifetimes measured in TOL (Table S1†), as well as with the negligibly small fluorescence quantum yields in polar media. Altogether, these TA data do not give much detail on the nature of the excited state. The similarity of the TA spectra of the 1B and 2B dyes could be interpreted as a localisation of the excitation on one branch for the 2B dyes. However, no information on whether the excitation is delocalised over the whole D–A–D′ branch or localised on either the D–A or the A–D′ sides can be deduced.

### 2.5 Time-resolved IR spectroscopy

#### 2.5.1 One-branched (1B) dyes

**Fig. 6:**
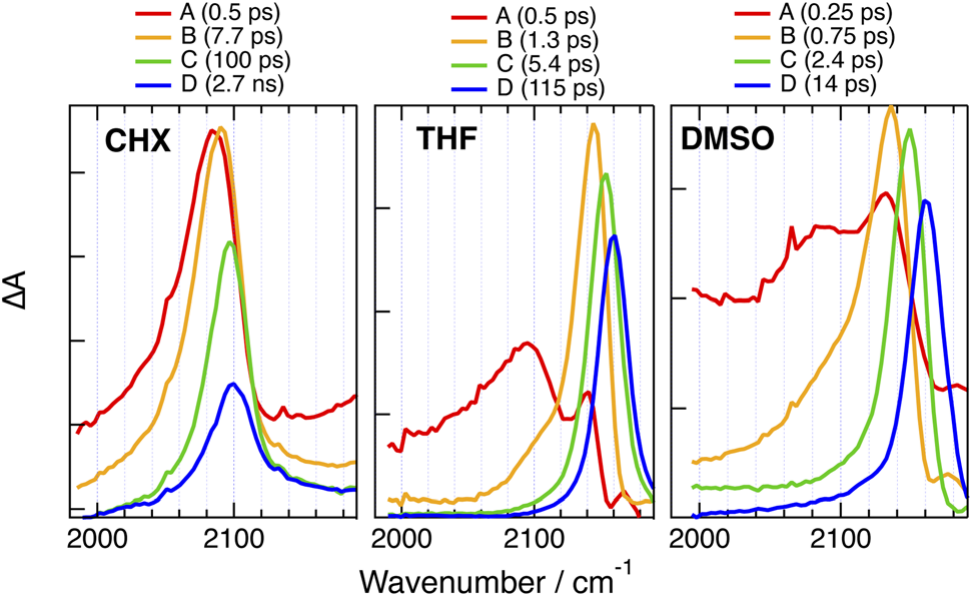
Evolution-associated difference absorption spectra and time constants obtained from a global analysis of the time-resolved IR absorption data measured upon 400 nm excitation of PT-CARs in various solvents assuming a series of successive exponential steps.

Time-resolved IR (TRIR) measurements in the –C≡C– stretching region were carried out with all dyes in CHX and/or TOL, as well as in DMSO and, in some cases, in THF to obtain deeper insight into the localisation of the excitation. We first discuss the results with the 1B dyes, starting with PT-CARs, which bears a single –C≡C– marker. Fig. 6 and S24A† show the EADS and time constants obtained from a global analysis of the TRIR data recorded with this dye assuming a series of successive exponential steps, the TRIR spectra being presented in the ESI (Fig. S22 and S23†). The early spectra in all solvents exhibit a broad band around 2080 cm^−1^. This band narrows and shifts to higher frequency in a few ps to an extent that increases with the solvent polarity, namely to 2100 cm^−1^ in CHX, 2140 cm^−1^ in TOL and 2160 cm^−1^ in THF and DMSO. Afterward, the band decays completely on a timescale ranging from ns in CHX and TOL to a few ps in DMSO.

The spectral dynamics in CHX can be attributed to structural and vibrational relaxation. As excitation was done at 400 nm on the high-energy side of the S_1_ ← S_0_ band, planarisation around the –C≡C– bond could also contribute, as already observed with other ethynyl-based D–A systems.^59,66^ To quantify this contribution, TRIR measurements were repeated upon 530 nm excitation, *i.e.* on the red-edge of the absorption band (Fig. S23†). In this case, the transient spectra are qualitatively the same as those at 400 nm excitation except at early time, where the band is narrower and about 7 cm^−1^ up-shifted (Fig. S24B†).

Consequently, most of the spectral dynamics observed in TOL and more polar solvents can be attributed to the equilibration of the CT state upon solvent relaxation. Polar solvation favours localisation of the charges on the D and A sub-units, leading to a smaller density of the excited electron on the –C≡C– bond, *i.e.*, a weaker π* character. This results in an increase of the –C≡C– bond order and, consequently, the –C≡C– stretching frequency up-shifts towards its value in the electronic ground state, which is above 2200 cm^−1^ (Fig. S6†).^33,67^ These results suggest that in polar solvents, the CT excitation in PT-CARs is mostly localised on the PT–BTD D–A pair. If the excitation were on the BTD-CARs end, there would be little excitation on the alkyne bond and, consequently the –C≡C– stretching band should not be visible in the TRIR spectra or would at least be much weaker.^47^

**Fig. 7:**
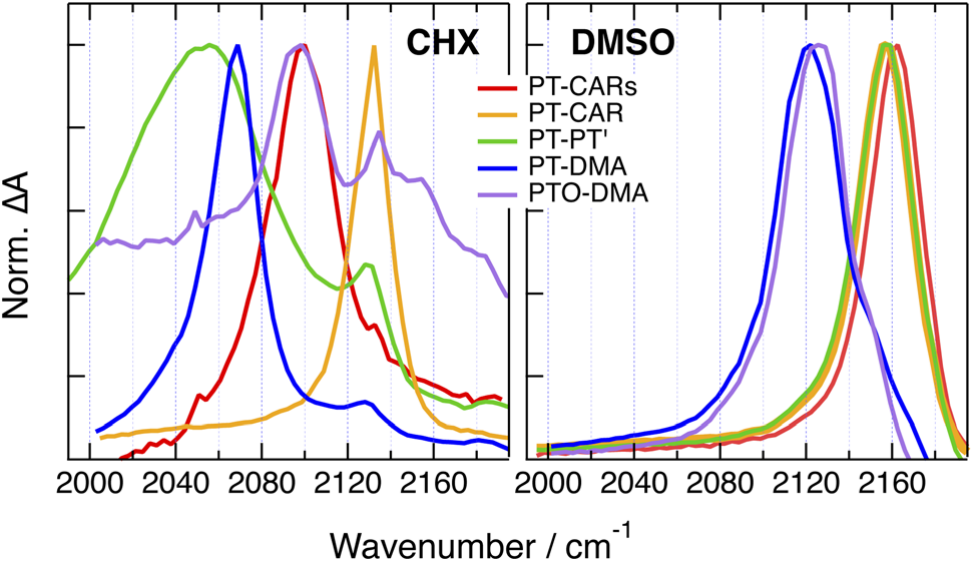
Comparison of the evolution-associated difference absorption spectra of the relaxed excited state of 1B dyes in non-polar (CHX) and highly-polar (DMSO) solvents.

Qualitatively similar TRIR dynamics are observed with the other 1B dyes as discussed in detail in the ESI (Fig. S25–S32†). Here, we concentrate on the spectra associated with the relaxed excited state in CHX and in DMSO. As illustrated in Fig. 7A, the late spectra measured with PT-CAR in CHX also exhibit a single –C≡C– band, but it is up-shifted by 30 cm^−1^. This difference suggests that excitation is not centred on the PT–BTD pair only but is delocalised over the whole molecule. In this case, the band corresponds to the antisymmetric –C≡C– stretching mode. The band due to the symmetric stretching vibration is probably too weak and/or too close to the antisymmetric one to be visible. In DMSO, however, the –C≡C– band is at the same frequency as with PT-CARs (Fig. 7B), indicating a localisation of the excitation at the PT–BTD end of the molecule.

The late TRIR spectra measured in CHX with the other three 1B dyes, namely, PT-PT′, PT-DMA and PTO-DMA, exhibit two bands of different intensity (Fig. 7A), which probably correspond to the antisymmetric and symmetric stretching modes, in agreement with a delocalised excitation. As these molecules are not centro-symmetric, the symmetric stretching mode is not IR forbidden. However, its intensity can be expected to be much smaller than that of the antisymmetric stretching mode. By contrast, a single band is visible in DMSO (Fig. 7B). For PT-PT′, it is at the same frequency as for PT-CARs and PT-CAR, indicative of a localisation at one of the two ends of the molecule, PT–BTD or BTD-PT′. For PT-DMA and PTO-DMA, the band is down-shifted by about 40 cm^−1^. This difference suggests that, for these two dyes, excitation is localised at the other end of the molecule, namely on the BTD–DMA side. This is consistent with the stronger donating ability of DMA relative to PT and PTO.

#### 2.5.2 Two-branched (2B) dyes

**Fig. 8:**
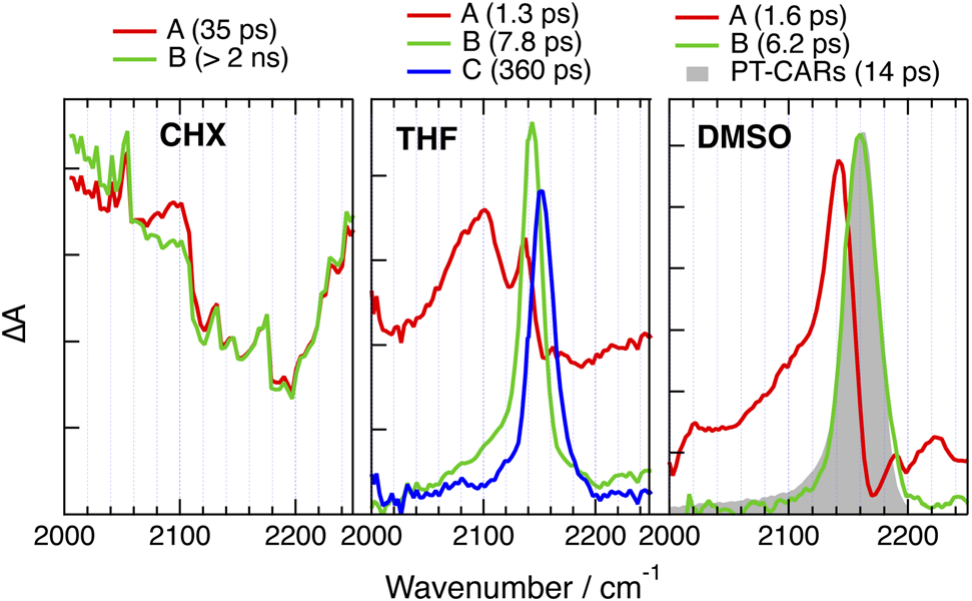
Evolution-associated difference absorption spectra and time constants obtained from a global analysis of the time-resolved IR absorption data measured upon 530 nm (CHX) or 400 nm (THF, DMSO) excitation of CARs-PT-CARs assuming a series of successive exponential steps. For comparison, the last spectrum obtained with PT-CARs in DMSO is shown in grey.

We now consider the 2B dyes starting with CARs-PT-CARs, which contains only two –C≡C– markers. As illustrated in Fig. 8 and S33,† the TRIR spectra measured in CHX upon red-edge excitation at 530 nm exhibit broad continuous absorption over the whole spectral window with a minimum around 2200 cm^−1^, where the ground state bleach of the –C≡C– IR band is expected (Fig. S6†). The spectra remain mostly unchanged within the time window of the experiment. Such broad background signal is also present at early time in THF, but transforms in about 1 ps into a spectrum with a single band around 2143 cm^−1^, which shifts further to 2150 cm^−1^, before decaying in 360 ps. Similar but faster initial dynamics are visible in DMSO and the resulting spectrum is essentially the same as that measured with the 1B analogue with the –C≡C– band at 2160 cm^−1^ (Fig. 8).

Similarly broad TRIR signals have already been reported with several symmetric two- and three-branched D–A molecules in non-polar solvents.^46,53,68^ They were shown to be due to an electronic transition from the delocalised quadrupolar/octupolar S_1_ state and to decay concurrently to ESSB. This background leads to a distortion of the narrower overlapping vibrational bands, similar to a Fano resonance.^69^ The presence of this signal over the whole excited-state lifetime of CARs-PT-CARs in CHX points to an excitation distributed over both branches. However, as polarity increases, *e.g.* in THF and DMSO, ESSB takes place, the background decays entirely and the TRIR spectrum becomes similar to that measured with the 1B analogue (Fig. 8).

Interestingly, the initial spectra recorded in CHX upon 400 nm excitation are dominated by a distinct band at 2090 cm^−1^, but transform in about 40 ps into the same spectrum as measured upon red-edge excitation (Fig. S33 and S34†). This observation suggests that excitation is first localised and then redistributes over the whole molecule. As discussed above, the S_1_ ← S_0_ absorption band is inhomogeneously broadened as a consequence of the torsional disorder in the ground state. Whereas red-edge excitation photoselects planar molecules, irradiation at 400 nm leads to the excitation of disordered molecules. According to the data, the electronic excitation in these distorted molecules is first localised and then spreads over the whole molecule as planarisation takes place. Similar localisation due to distortion was reported recently.^70^

The TRIR dynamics recorded with the other 2B dyes are not largely different from those measured with CARs-PT-CARs and are discussed in detail in the ESI (Fig. S35–S42†). Here, we concentrate on the nature of the equilibrium excited state in the least and most polar solvents. Broad background signal is present with all 2B dyes in CHX or TOL, except for DMA-PTO-DMA. This background, which decays rapidly in polar solvents, points to a delocalised excited state in non-polar media. Its absence with DMA-PTO-DMA does not necessary imply a localised excitation, but could be due to an electronic transition lying outside the spectral window of the experiment.

**Fig. 9:**
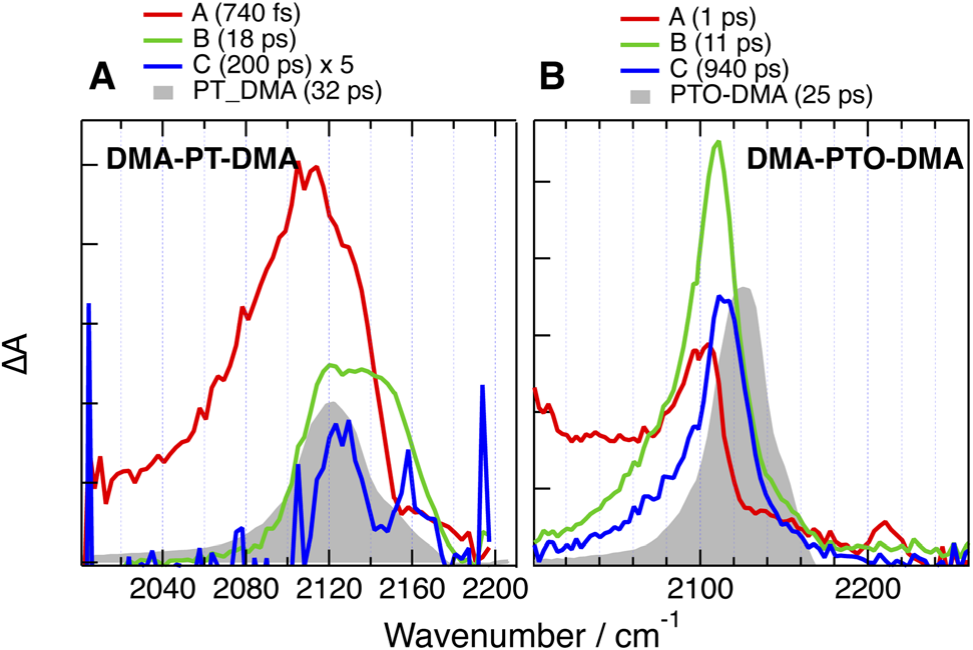
Evolution-associated difference absorption spectra and time constants obtained from a global analysis of the time-resolved IR absorption data measured with (A) DMA-PT-DMA and (B) DMA-PTO-DMA in DMSO assuming a series of successive exponential steps and last spectra obtained with the single branch analogues (grey).

**Fig. 10:**
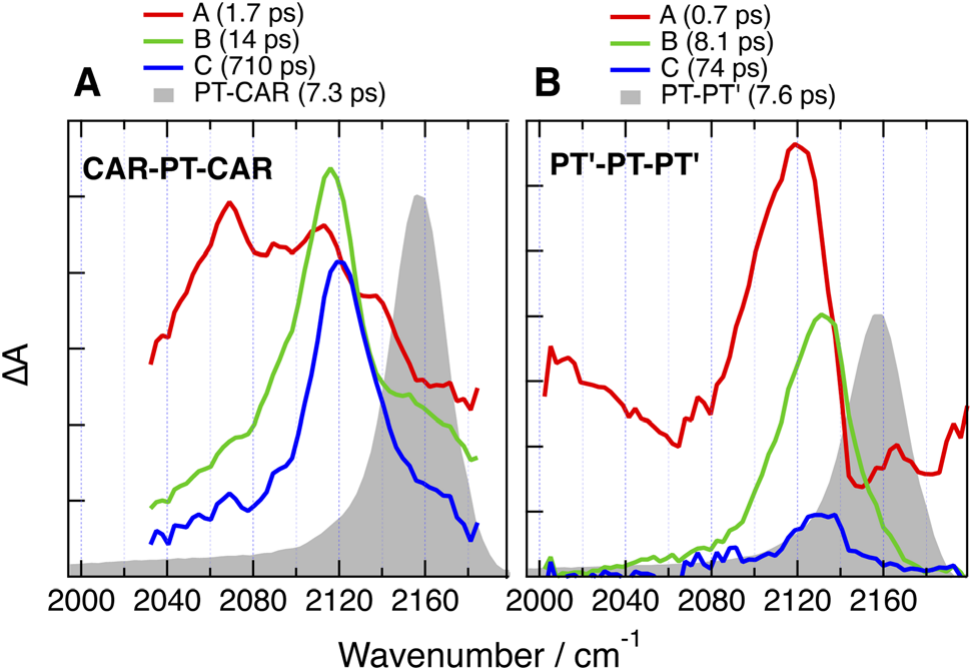
Evolution-associated difference absorption spectra and time constants obtained from a global analysis of the time-resolved IR absorption data measured with (A) CAR-PT-CAR and (B) PT′-PT-PT′ in DMSO assuming a series of successive exponential steps and last spectra obtained with the single branch analogues (grey).

Like for CARs-PT-CARs, the electronic background, if present, decays in less then a ps in DMSO, and a single band is observed in the late spectra (Fig. 9 and 10). For DMA-PT-DMA and DMA-PTO-DMA, this band is at very similar frequency than that measured with the 1B analogues, indicating, that after the initial ES-SB, excitation is funnelled toward one of the two BTD–DMA ends of these molecules. Interestingly, the TRIR spectra of DMA-PT-DMA at intermediate time delays (EADS B in Fig. 9A) show a broad band with a shoulder around 2150 cm^−1^ that is consistent with the PT–BTD –C≡C– vibration. This could indicate that excitation first localises on a single branch with a possible transient equilibrium between the PT–BTD and BTD–DMA CT states, and finally concentrates on a BTD–DMA end as solvent relaxation takes place. It should be noted that the lifetime of the relaxed excited state is markedly longer than for the 1B analogues.

Unlike for the 2B dyes discussed above, the band in the late TRIR spectra of CAR-PT-CAR is not at the same frequency as for PT-CAR, but is down-shifted by 40 cm^−1^ (Fig. 10A). This implies that excitation is not localised on a PT–BTD pair like for the 1B dye, but is rather centred on one of the two BTD-CAR ends. However, in less polar solvents, TOL and THF, the band in the late spectra is at similar frequency as the 1B analogue (Fig. S35 and S36†), suggesting that, in these weak to medium polar environments, excitation is on a central PT–BTD pair. Similar behaviour is found with PT′-PT-PT′, the –C≡C– band being down-shifted by ∼25 cm^−1^ relative to PT-PT′. This frequency shift could arise from a slight disparity in donating strength of PT and PT′ due to their different substituents on the N atom. In these cases again, the decay of the relaxed excited state is slower than for the 1B analogues.

## 3 Discussion

**Fig. 11:**
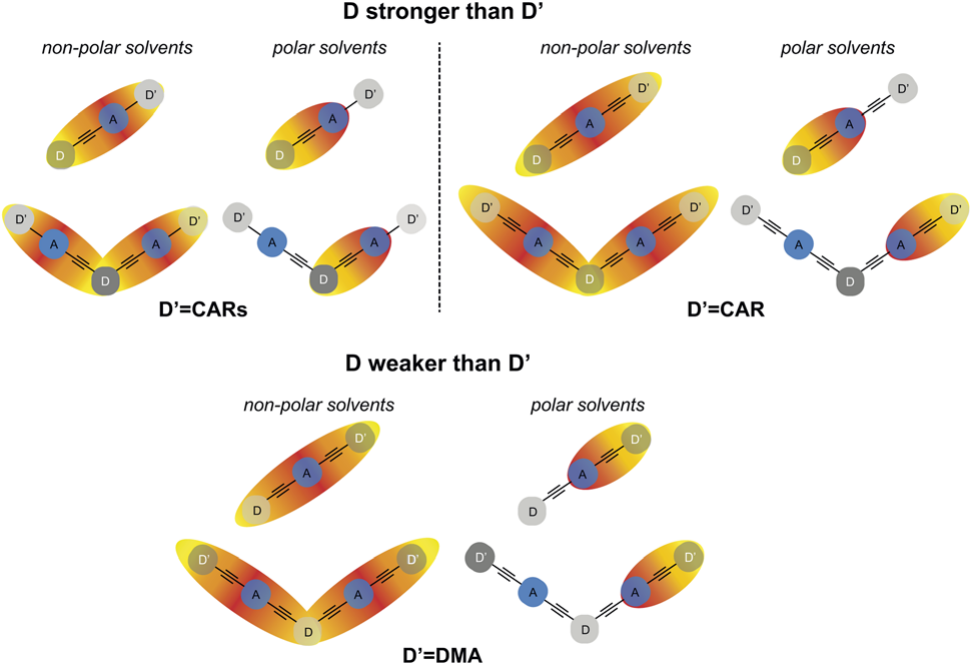
Schematic representation of the spatial distribution of the electronic excitation in single- and double-branch dyes in non-polar and polar solvents when D is a stronger donor than D′ and *vice versa*. For the two-branched dyes in polar solvents, excitation has the same probability to localise at the other end of the molecule. Red and yellow colours represent negative and positive charges, respectively.

The TRIR results obtained with the 1B dyes can be summarised as follows. In non- and weakly-polar solvents (CHX, TOL), excitation is mostly delocalised over the whole molecule (Fig. 11). In polar solvents, the large frequency up-shift of the –C≡C– band reflects an increase of CT character and the localisation of the excitation at one end of the molecule. The side at which localisation occurs depends on the relative electron donating strength of the D and D′ sub-units (Fig. 11). For PT-CARs, and PT-CAR, D is stronger than D′ and excitation localises on the D–A end, *i.e.*, on the PT–BTD pair. For PT-DMA and PTO-DMA, D′ is stronger than D and excitation localises on the A–D′ end, namely, on the BTD–DMA pair. For PT-PT′, the late spectra show the same band as for PT-CARs and PT-CAR, suggesting localisation on the PT–BTD side as well. However, given the close similarity of PT and PT′, localisation on the other end cannot be ruled out.

For the 2B dyes in non- and weakly-polar solvents (CHX and TOL), excitation is delocalised over the two branches, as testified by the characteristic long-lived electronic background signal (Fig. 11). This broad feature leads to a distortion of the narrower vibrational bands and inhibits a precise determination of their frequency. DMA-PTO-DMA makes an exception, as this background is not visible. In this case, the electronic transition could be at an energy outside the spectral window of the TRIR experiment. However, considering the relative large difference of electron donating strength between D (PTO) and D′ (DMA), a localisation of the excitation at one of the A–D′ ends of the molecule cannot be ruled out.

In polar solvents, this background is absent or decays very rapidly, indicating the localisation of the excitation. For CARs-PT-CARs, DMA-PT-DMA and DMA-PTO-DMA, the late spectra are similar to those measured with their 1B analogues, pointing to a localisation on the donor–acceptor pair with the stronger donor, *i.e.*, PT–BTD for the first and BTD–DMA for the two others. By contrast, for CAR-PT-CAR and PT′-PT-PT′, the late spectra are clearly distinct from those of the 1B analogues. This implies that excitation does not localise on the pair with the stronger donor, but rather on a terminal pair, *i.e.*, BTD-CAR and BTD-PT′, respectively.

In other words, for all the 2B dyes but CARs-PT-CARs in highly polar solvents, excitation always localises at one of the two ends of the molecule, independently of the relative donating strength of the central and terminal donors (Fig. 11). Two hypotheses can be proposed to account for this a priori unexpected behaviour.

(1) The donating strength of the central PT moiety is weaker in the 2B than in the 1B dyes, because of the conjugation of PT with the second A–D′ arm. Such an effect is supported by quantum-chemistry calculations of two phenothiazine derivatives, one with an alkyne group on position 3 and one with alkyne groups on positions 3 and 8. They predict the HOMO of the di-substituted molecule to be lower in energy by about 60 meV than that of the mono-substituted one. However, this difference is small and the gas-phase calculations still predict PT to be a stronger donor than CAR even for the 2B molecule.

(2) The solvation energy of a CT state localised at one end of a 2B molecule is larger than that of a CT state located at the centre. Indeed, in the first case, the hole is on a terminal donor, D′, and should be better exposed to the solvent. Such larger interaction with the solvent is supported by molecular dynamics (MD) simulations of CAR-PT-CAR and PT′-PT-PT′ in DMSO, discussed in more detail in the ESI (Fig. S43 and S44†). For CAR-PT-CAR, the gain in solvation energy upon localisation of the hole on a CAR sub-unit probably compensates for its smaller donating strength. This results in the stabilisation of the BTD-CAR CT state relative to the PT–BTD CT state. In less polar solvents, this gain in solvation energy is not sufficient and the hole localises on the stronger donor, *i.e.* on the central PT unit, as observed in THF.

CARs-PT-CARs differs from the other 2B molecules, as the CT excitation does not localise at a terminal A–D′ pair even in highly polar solvents (Fig. 11). This can also be explained in terms of solvation energy. As the CAR group is directly linked to the BTD acceptor, the electric dipole moment of the corresponding CT state is relatively small compared to that of BTD and CAR linked *via* an phenyl-ethynyl group. Given that the solvation energy of an electric dipole scales with the square of its size, it cannot compensate for the weaker donating strength of CAR relative to PT, contrary to the case of CAR-PT-CAR.

The results with the 2B dyes reveal that ESSB already takes place in medium polar solvents like THF. As mentioned in the introduction and discussed in more detail in ref. 44,45, ESSB occurs as soon as the loss of excitonic interbranch coupling energy, *V*_ib_, is compensated by a gain in solvation energy. Consequently, if the interbranch coupling is strong, *i.e.* in the 2000–3000 cm^−1^ range as observed previously with linear quadrupolar A–π–D–π–A and D–π–A–π–D dyes,^44,45^ ESSB may only result in a partial localisation of the excitation on one branch even in the most polar solvents. The localisation observed here in THF points to a relatively weak interbranch coupling. This agrees well with the *V*_ib_ values in the 450–600 cm^−1^ range deduced from the quantum-chemical calculations (Table S3†) and those in the 250–400 cm^−1^ range extracted from the one- and two-photon absorption spectra of CARs-PT-CARs and CAR-PT-CAR.

These values are also consistent with the blue-shift of the S_1_–S_0_ absorption and emission bands of a few hundreds of cm^−1^ observed by going from the 2B to the 1B dyes in CHX, which should also reflect the loss of interbranch coupling. The very large shift of the fluorescence spectrum found here with the 1B dyes when going from CHX to TOL points to a large dipole moment of the symmetry-broken state. This implies that the gain in solvation energy upon ESSB should easily exceed *V*_ib_ in a medium polar solvent like THF. These relatively small *V*_ib_ are probably due to the V shape of the central PT unit, which decreases the conjugation between the two branches.

Further evidence of the weak interbranch coupling of these dyes is that torsional disorder is sufficient for ESSB to take place transiently in a non-polar solvent, as observed with CARs-PT-CARs in CHX (Fig. S34†). Of course in this case, a symmetric distribution of the excitation is rapidly recovered upon planarisation of the branches, which enhances conjugation. However, this observation reveals that torsion around an ethynyl bond is enough to make interbranch coupling negligibly small and enable ESSB.

Finally, the above results reveal that the lifetime of the equilibrated excited state of the 2B dyes in the highly polar DMSO is longer than that of the 1B dyes (Fig. 9 and 10). This effect cannot be explained by a different localisation of the excitation, like it is the case for CAR-PT-CAR, because it is also present with DMA-PT-DMA and DMA-PTO-DMA. Such differences are not unique to these dyes, but were also observed with a fluorenone-based linear D–A–D molecules in THF.^33^ Given the strong CT character of the excited state in polar solvents, the decay of the relaxed excited states of these dyes can be considered as an intramolecular charge recombination (CR) process. The dynamics of such CR is known to be very sensitive to the energy gap between the CT and ground states, and to accelerate with decreasing gap,^71,72^ as predicted by Marcus electron transfer theory for the inverted region.^73^ This explains the spectacular decrease of the excited-state lifetime of the 1B dyes observed upon increasing solvent polarity, *i.e.* from ns to hundreds of ps and to a few ps when going from CHX to THF and DMSO, respectively. The large spectral shift of the fluorescence observed by going from CHX to TOL points to a strong decrease of the energy gap for CR upon increasing solvent polarity. An acceleration of the CR dynamics is also observed with the 2B dyes, but is less pronounced, changing from a few ns in CHX to hundreds of ps in DMSO. Different solvation energies of the CT states of the 1B and 2B dyes could possibly account for this effect. Given that dipole–dipole interaction is of relatively long-range nature, the presence of the second ‘inactive’ arm in the 2B dyes can be expected to have a detrimental effect on the solvation energy, compared to the 1B analogues. This should result in a larger energy gap for CR for the 2B dyes. The smaller spectral shift of the fluorescence of the 2B dyes compared to the 1B analogues observed between CHX and TOL, supports this hypothesis. In polar solvents, fluorescence is too far in the near IR and too weak to be measured. Therefore, differences in the energy gap for CR in polar solvents cannot be confirmed. In the case of the above-mentioned fluorenone-based D–A–D molecule, the fluorescence could be detected in the more polar diethylether.^33^ The spectral shift in this solvent relatively to CHX was smaller by a factor two compared to the D–A analogue, supporting our hypothesis.

## 4 Conclusions

This work evidences the subtle dependence of the localisation of charge-transfer excitons in asymmetric D–π–A–π–D′ molecules and in their symmetric double-branch analogues on the relative strength of the donors, on the D–A distance and on the environment. In non-polar media, excitation is mostly delocalised over the whole molecule in both cases, conferring a strong quadrupolar character to the excited state. In polar media, solvation energy favours a localisation to a dipolar CT excited state. Our results reveal that the location of the excitation is not simply governed by the relative donating strength of D and D′, but also by the solvation energy of the resulting CT state, which is larger for a CT state located at one end of the molecule than in the centre. Solvation energy is also larger for a CT state with the D and A states separated by a phenyl-ethynyl group rather than directly linked. In single-branch dyes, where both ends are equally well solvated, the CT exciton localises in the side with the stronger donor. This is no longer the case of the two-branched dyes, for which the exciton always localises at a branch end due to a larger solvation energy. The exception is CARs-PT-CARs, for which solvation energy is smaller for localising at a branch end than at the centre because of the direct linkage of the CAR donor to the BTD acceptor.

This investigation demonstrates how the location of a CT exciton in multibranched DA molecules can be predicted and possibly controlled by tuning not only the nature of the donor and acceptor constituents, but also the distance between them and the environment. In principle, a similar behaviour as that observed here with alkyne spacers can be expected with other conjugated spacers, such as phenylenes or thienylenes. However, because of the lack of specific vibrational markers, information about the localisation of the excitation would not be easily accessible. This results represent a significant step towards the development of an ‘addressable site-specific photochemistry’ of large conjugated molecular architectures.

## Data availability

All data can be downloaded from https://doi.org/10.26037/yareta:kspwkaeskfavjec5wmt23cw4e.

## Author contributions

E. Balanikas, T. Bianconi, and P. Mancini designed and performed the spectroscopic experiments, analysed the data and contributed to the initial draft. B. Carlotti supervised the stationary and electronic transient absorption experiments. N. J. Tiwari and M. Sheokand synthesised the compounds under the supervision of R. Misra. E. Vauthey supervised the transient IR study, performed the QM calculations and MD simulations and wrote the final version with the help of all authors.

## Conflicts of interest

There are no conflicts to declare.

## Supplementary Material

SC-OLF-D5SC01257K-s001
