# Supplementary material for: Controlling the spatial distribution of electronic excitation in asymmetric D–A–D′ and symmetric D′–A–D–A–D′ electron donor–acceptor molecules
Source: Chem Sci. 2025 Apr 4;16(19):8443–53. doi: 10.1039/d5sc01257k (PMC11987036; doi:10.1039/d5sc01257k)
Supplement: SC-016-D5SC01257K-s001 [file SC-016-D5SC01257K-s001.pdf]

Supplementary Information (SI) for Chemical Science.
This journal is © The Royal Society of Chemistry 2025

Controlling the spatial distribution of electronic excitation in asymmetric D-A-D' and symmetric D'-A-D-A-D' electron donor-acceptor molecules

Evangelos Balanikas[a††‡], Tommaso Bianconi[b¶‡], Pietro Mancini[b], Nikhil Ji Tiwari[c], Manju Sheokand[c], Rajneesh Misra[*c], Benedetta Carlotti[*b], and Eric Vauthey[*a]

[a] *Department of Physical Chemistry, University of Geneva, 30 Quai Ernest-Ansermet, CH-1211 Geneva 4, Switzerland. E-mail: eric.vauthey@unige.ch*
[b] *Department of Chemistry, Biology and Biotechnology, University of Perugia, via elce di sotto 8, 06123 Perugia, Italy. E-mail: benedetta.carlotti@unipg.it*
[c] *Department of Chemistry, Indian Institute of Technology, Indore 453552, India. E-mail: rajneeshmisra@iiti.ac.in*

‡ These authors contributed equally to this work.
†† Present address: Laboratoire d'Optique et Biosciences, Ecole Polytechnique, CNRS-INSERM, Institut Polytechnique de Paris, 91120 Palaiseau, France
¶ Present address: Department of Chemistry, University of Wisconsin-Madison, University Ave 1101, 53706 Madison (WI), USA

Contents

S1 Experimental Details **6**
S1.1 Synthesis and characterisation . . . 6
S1.2 Stationary electronic spectroscopy . . . 11
S1.3 Time-resolved fluorescence . . . 11
S1.4 Electronic transient absorption spectroscopy . . . 11
S1.5 Time-resolved IR spectroscopy . . . 11
S1.6 Quantum-chemical calculations . . . 11
S1.7 Molecular dynamics simulations . . . 12

S2 Additional results **13**
S2.1 Stationary electronic spectroscopy . . . 13
S2.2 Stationary vibrational spectroscopy . . . 15
S2.3 Quantum-chemical calculations . . . 15
S2.4 Electronic transient absorption spectroscopy . . . 18
S2.5 Time-resolved IR spectroscopy . . . 22
S2.6 Molecular Dynamics (MD) simulations . . . 38
S2.7 NMR spectroscopy and MS spectrometry . . . 39

List of Figures

S1 Stationary electronic absorption and fluorescene spectra of **PT-CARs** and **CARs-PT-CARs** in various solvents. CHX: cyclohexane; TOL: toluene; THF: tetrahydrofuran; DMSO: dimethylsulfoxide. 13

S2 Stationary electronic absorption and fluorescene spectra of **PT-CAR** and **CAR-PT-CAR** in various solvents. The long-wavelength tail of the spectrum of **CAR-PT-CAR** in CHX point to the formation of aggregates. 13

S3 Stationary electronic absorption and fluorescene spectra of **PT-PT'** and **PT'-PT-PT'** in various solvents. 13

S4 Stationary electronic absorption and fluorescene spectra of **PT-DMA** and **DMA-PT-DMA** in various solvents. 14

S5 Stationary electronic absorption and fluorescene spectra of **PTO-DMA** and **DMA-PTO-DMA** in various solvents. 14

S6 Stationary IR absorption spectra of **PT-PT'** and **PT'-PT-PT'** in toluene. 15

S7 Ground-state optimised geometry of **CARs-PT-CARs**. 15

S8 Frontier molecular orbitals involved in the first two electronic transitions of **CAR-PT-CAR** and associated oscillator strength. 16

S9 Frontier molecular orbitals involved in the first two electronic transitions of **PT'-PT-PT'** and associated oscillator strength. 16

S10 Frontier molecular orbitals involved in the first two electronic transitions of **DMA-PT-DMA** and associated oscillator strength. 17

S11 Transient electronic absorption recorded upon 400 nm excitation of **PT-DMA** in TOL (left), THF (middle), and DMSO (right). 18

S12 Transient electronic absorption recorded upon 400 nm excitation of **PT-PT'** in TOL (left), THF (middle), and DMSO (right). 18

S13 Evolution-associated difference absorption spectra and time constants obtained from a global analysis of the data shown in Figure S12 assuming a series of successive exponential steps. . . . 18

S14 Transient electronic absorption recorded upon 400 nm excitation of **PT'-PT-PT'** in TOL (left) and DMSO (right). 19

S15 Evolution-associated difference absorption spectra and time constants obtained from a global analysis of the data shown in Figure S14 assuming a series of successive exponential steps. . . . 19

S16 Transient electronic absorption recorded upon 400 nm excitation of **DMA-PT-DMA** in TOL (left), THF (middle), and DMSO (right). 20

S17 Evolution-associated difference absorption spectra and time constants obtained from a global analysis of the data shown in Figure S16 assuming a series of successive exponential steps. . . . 20

S18 Transient electronic absorption recorded upon 400 nm excitation of **PTO-DMA** in TOL (left), THF (middle), and DMSO (right). 21

S19 Evolution-associated difference absorption spectra and time constants obtained from a global analysis of the data shown in Figure S18 assuming a series of successive exponential steps. . . . 21

S20 Transient electronic absorption recorded upon 400 nm excitation of **DMA-PTO-DMA** in TOL (left), THF (middle), and DMSO (right). 21

S21 Evolution-associated difference absorption spectra and time constants obtained from a global analysis of the data shown in Figure S20 assuming a series of successive exponential steps. . . . 21

S22 Transient IR absorption recorded upon 400 nm excitation of **PT-CARs** in CHX (left), THF (centre), and DMSO (right). 22

S23 Transient IR absorption recorded with **PT-CARs** in TOL upon 400 nm excitation (left) and in CHX upon 530 nm excitation (right). 22

S24 Evolution-associated difference absorption spectra and time constants obtained from a global analysis of the TRIR data shown in Figure S23 assuming a series of successive exponential steps. . 22

S25 Transient IR absorption recorded with **PT-CAR** upon 400 nm excitation in CHX (top left), TOL (top right), THF (bottom left) and upon 530 nm excitation in DMSO (bottom right). 23

S26 Evolution-associated difference absorption spectra and time constants obtained from a global analysis of the TRIR data shown in Figure S25 assuming a series of successive exponential steps. 24
S27 Transient IR absorption recorded upon 530 nm excitation of **PT-PT'** in CHX (top left), TOL (top right), THF (bottom left) and DMSO (bottom right). 25
S28 Evolution-associated difference absorption spectra and time constants obtained from a global analysis of the TRIR data shown in Figure S27 assuming a series of successive exponential steps. 26
S29 Transient IR absorption recorded with **PT-DMA** in CHX (400 nm excitation, top left) and in TOL (top right), THF (bottom left) and DMSO (bottom right) upon 530 nm excitation. 27
S30 Evolution-associated difference absorption spectra and time constants obtained from a global analysis of the TRIR data shown in Figure S29 assuming a series of successive exponential steps. 28
S31 Transient IR absorption recorded upon 400 nm excitation of **PTO-DMA** in CHX (top left), TOL (top right), THF (bottom left) and DMSO (bottom right). 29
S32 Evolution-associated difference absorption spectra and time constants obtained from a global analysis of the TRIR data shown in Figure S31 assuming a series of successive exponential steps. 30
S33 Transient IR absorption recorded upon 530 nm excitation of **CARs-PT-CARs** in CHX (top left) and upon 400 nm excitation in CHX(top right), THF (bottom left) and DMSO (bottom right). 31
S34 Evolution-associated difference absorption spectra and time constants obtained from a global analysis of the TRIR data measured with **CARs-PT-CARS** in CHX upon 400 nm excitation assuming a series of successive exponential steps. 31
S35 Transient IR absorption recorded with **CAR-PT-CAR** upon 400 nm excitation in TOL (left), and DMSO (right), and upon 530 nm excitation in THF (middle), ... 32
S36 Evolution-associated difference absorption spectra and time constants obtained from a global analysis of the TRIR data shown in Figure S35 assuming a series of successive exponential steps. 32
S37 Transient IR absorption recorded with **PT'-PT-PT'** upon 530 nm excitation in TOL (top right), THF (bottom left) and 400 nm excitation in CHX (top left) and DMSO (bottom right). 33
S38 Evolution-associated difference absorption spectra and time constants obtained from a global analysis of the TRIR data shown in Figure S37 assuming a series of successive exponential steps. 34
S39 Transient IR absorption recorded with **DMA-PT-DMA** upon 530 nm excitation in TOL (top right), THF (bottom left) and 400 nm excitation in CHX (top left) and DMSO (bottom right). 35
S40 Evolution-associated difference absorption spectra and time constants obtained from a global analysis of the TRIR data shown in Figure S39 assuming a series of successive exponential steps. 36
S41 Transient IR absorption recorded with **DMA-PTO-DMA** upon 530 nm excitation in TOL (left) and THF (middle) and 400 nm excitation in DMSO (right). 37
S42 Evolution-associated difference absorption spectra and time constants obtained from a global analysis of the TRIR data shown in Figure S41 assuming a series of successive exponential steps. 37
S43 Snapshot of a MD simulation of **CAR-PT-CAR** (left) and **PT'-PT-PT'** (right) in DMSO. 38
S44 Histogram of the number of DMSO molecules within a centre-of-mass (COM) distance of 6 nm of the central PT and one of the terminal carbazole (left) or PT' (right) units of **CAR-PT-CAR** and **PT'-PT-PT'**, respectively. This illustrates the larger exposure to solvent of the end donors, D', compared to the central donor, D. 38
S45 ^{1}H NMR spectrum of **PT-CARs**. 39
S46 ^{13}C NMR spectrum of **PT-CARs**. 39
S47 High resolution MS spectrogram of **PT-CARs**. 40
S48 ^{1}H NMR spectrum of **PT-CAR**. 40
S49 ^{13}C NMR spectrum of **PT-CAR**. 41
S50 High resolution MS spectrogram of **PT-CAR**. 41
S51 ^{1}H NMR spectrum of **PT-PT'**. 42
S52 ^{13}C NMR spectrum of **PT-PT'**. 42
S53 High resolution MS spectrogram of **PT-PT'**. 43
S54 ^{1}H NMR spectrum of **PT-DMA**. 43

S55 ^{13}C NMR spectrum of **PT-DMA**. . . . 44
S56 High resolution MS spectrogram of **PT-DMA**. . . . 44
S57 ^{1}H NMR spectrum of **PT'-PT-PT'**. . . . 45
S58 ^{13}C NMR spectrum of **PT'-PT-PT'**. . . . 45
S59 MALDI-TOF of **PT'-PT-PT'**. . . . 46
S60 ^{1}H NMR spectrum of **DMA-PT-DMA**. . . . 47
S61 ^{13}C NMR spectrum of **DMA-PT-DMA**. . . . 47
S62 High resolution MS spectrogram of **DMA-PT-DMA**. . . . 48
S63 ^{1}H NMR spectrum of **PTO-DMA**. . . . 48
S64 ^{13}C NMR spectrum of **PTO-DMA**. . . . 49
S65 High resolution MS spectrogram of **PTO-DMA**. . . . 49
S66 ^{1}H NMR spectrum of **DMA-PTO-DMA**. . . . 50
S67 ^{13}C NMR spectrum of **DMA-PTO-DMA**. . . . 50
S68 High resolution MS spectrogram of **DMA-PTO-DMA**. . . . 51

List of Tables

S1 Fluorescence quantum yields, Φ_{fl}, and lifetimes. τ_{fl}, of the dyes in various solvents. Error on τ_{fl}: ±5%. 14
S2 Unscaled calculated -C≡C- stretching frequencies (in cm^{-1}) and IR intensity (in brackets, in km/mol) of the trans and cis conformers of **PT-CAR** in the S_0 and S_1 states. 17
S3 S_2-S_1 energy gap of the 2B dyes obtained from TD-DFT calculations. 17

S1 Experimental Details

S1.1 Synthesis and characterisation

The synthetic route of the phenothiazine and phenothiazine 5,5-dioxide functionalized asymmetric D-A-D' (**PT-CARs**, **PT-CAR**, **PT-PT'**, **PT-DMA**, and **PTO-DMA**) and symmetric D'-A-D-A-D' (**PT'-PT-PT'**, **DMA-PT-DMA**, and **DMA-PTO-DMA**) conjugated dyes are shown in Schemes S1–3. The Sonogashira cross-coupling reaction of benzothiadiazole derivatives 4-bromo-7-(9H-carbazol-9-yl)benzo[c][1,2,5]thiadiazole (**5**), 4-((4-(9H-carbazol-9-yl)phenyl)ethynyl)-7-bromobenzo[c][1,2,5]thiadiazole (**6**), and 3-((7-bromobenzo[c][1,2,5]thiadiazol-4-yl) ethynyl)-10-propyl-10H-phenothiazine (**7**) and 4-((7-bromobenzo[c][1,2,5]thiadiazol-4-yl)ethynyl)-N,N-dime thylaniline (**8**) with 3-ethynyl-10-octyl-10H-phenothiazine (**1**), resulted in the formation of phenothiazine functionalized asymmetric D-A-D' (**PT-CARs**, **PT-CAR**, **PT-PT'**, **PT-DMA**, and **PTO-DMA**), respectively. The derivatives **1**, **2**, **3**, **4**, **5**, **6**, and **7** were synthesized by the reported procedure. The reaction of 3-ethynyl-10-octyl-10H-phenothiazine (**1**) with 1.1 equivalent of benzothiadiazole functionalized derivatives **5**, **6**, **7**, and **8** in the presence of $Pd(PPh_3)_4$ as a catalyst and CuI as a co-catalyst in THF/TEA (1:1) solvent resulted in **PT-CARs**, **PT-CAR**, **PT-PT'**, and **PT-DMA** in 74%, 68%, 76%, and 78% yields, respectively (Scheme S1). Similarly, the Sonogashira cross-coupling reaction of 3,7-diethynyl-10-octyl-10H-phenothiazine (**2**) with 1.1 equivalent of benzothiadiazole functionalized derivatives **7** and **8** resulted in the formation of phenothiazine functionalized symmetric D'-A-D-A-D' (**PT'-PT-PT'** and **DMA-PT-DMA**) dyes in 56% and 52% yields, respectively (Scheme S2). Similarly, The Sonogashira cross-coupling reaction of benzothiadiazole derivative 4-((7-bromobenzo[c][1,2,5]thiadiazol-4-yl)ethynyl)-N,N-dimethylaniline (**8**) with 3-ethynyl-10-octyl-10H-phenothiazine 5,5-dioxide (**3**) and 3,7-diethynyl-10-octyl-10H-phenothiazine 5,5-dioxide (**4**) resulted in the formation of phenothiazine and phenothiazine 5,5-dioxide functionalized asymmetric (**PTO-DMA**) and symmetric (**DMA-PTO-DMA**) conjugated dyes in 72% and 68% yields respectively (Scheme S3). All the dyes were purified by column chromatography and further characterized by using NMR and HRMS spectroscopic techniques.

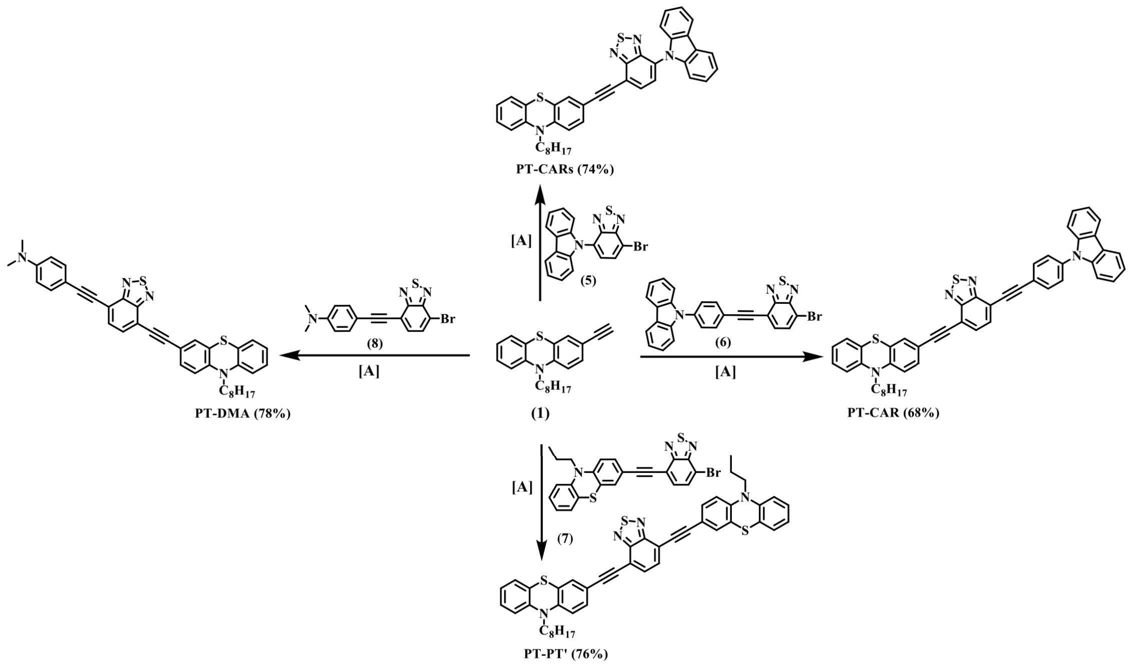

Scheme S1 Synthetic route of phenothiazine functionalized asymmetric D-A-D' (**PT-CARs**, **PT-CAR**, **PT-PT'**, and **PT-DMA**) conjugated dyes: [A] $Pd(PPh_3)_4$, CuI, THF:TEA (1:1), 70°C, 12 h.

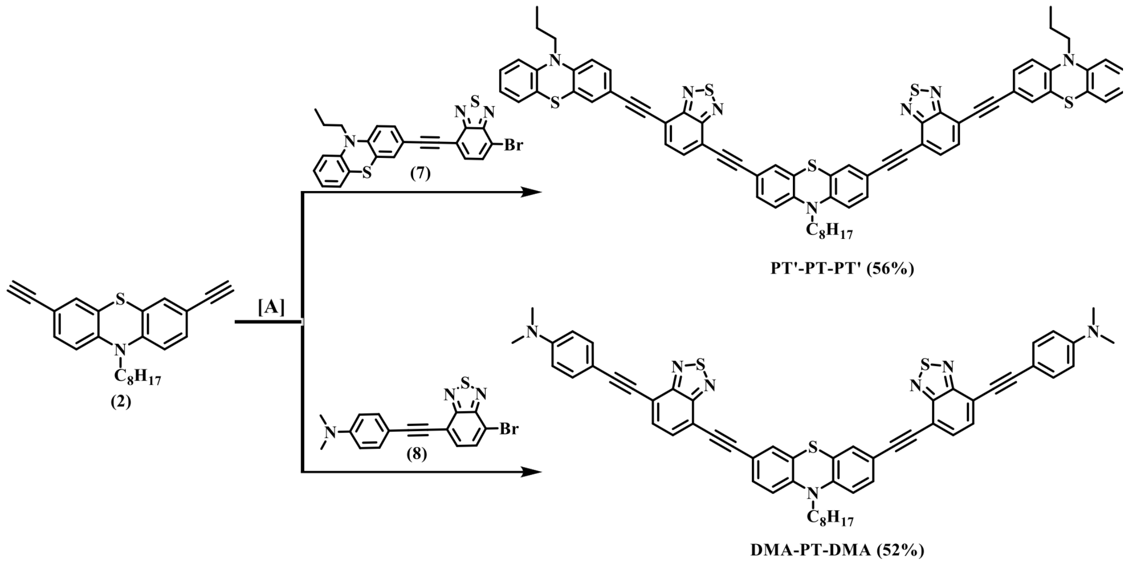

Scheme S2 Synthetic route of the phenothiazine functionalized symmetric D'-A-D-A-D' (**PT'-PT-PT'** and **DMA-PT-DMA**) conjugated dyes: [A] $Pd(PPh_3)_4$, CuI, THF:TEA (1:1), 70°C, 12 h.

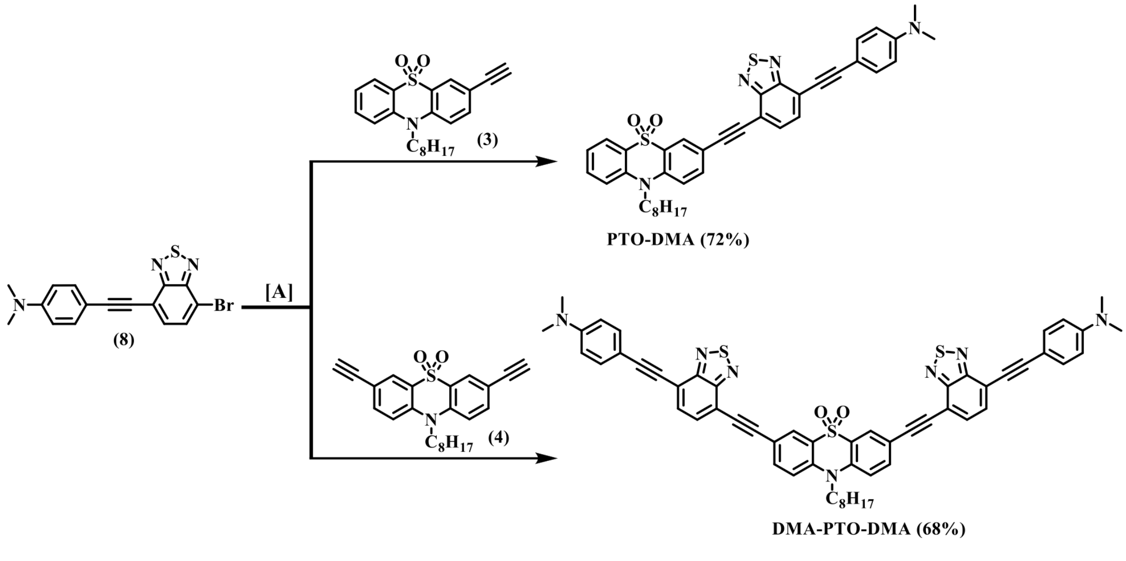

Scheme S3 Synthetic route of phenothiazine and phenothiazine 5,5-dioxide functionalized asymmetric (**PTO-DMA**) and symmetric (**DMA-PTO-DMA**) conjugated dyes; [A] $Pd(PPh_3)_4$, CuI, THF:TEA (1:1), 70°C, 12 h.

Chemicals were used as received unless otherwise indicated. All the oxygen- or moisture- sensitive reactions were carried out under an argon atmosphere, and the reflux reactions were performed in an oil bath. ^{1}H NMR (500 MHz) spectra were recorded on a Bruker 500 MHz FT-NMR spectrometer at room temperature. Chemical shifts are reported in delta (δ) units, expressed in parts per million (ppm) downfield from tetramethylsilane (TMS) using the residual protonated solvent as an internal standard $CDCl_3$, 7.26 ppm. The multiplicities are given as s (singlet), d (doublet), t (triplet) and m (multiplet) and the coupling constants, J, are given in Hertz. ^{13}C NMR (126 MHz) spectra were recorded on a Bruker 500 MHz FT-NMR spectrometer at room temperature. Chemical shifts are reported in delta (δ) units, expressed in parts per million (ppm) downfield from TMS using the solvent as internal standard $CDCl_3$, 77.16 ppm. All the measurements were carried out at 25°C. HRMS were recorded on a Bruker-Daltonics micrOTOF-Q II mass spectrometer.

Synthesis and characterization of 3-((7-(9H-carbazol-9-yl)benzo[c][1,2,5]thiadiazol-4-yl)ethynyl)-10-octyl-10H-phenothiazine (**PT-CARs**): In a 100 mL round bottomed flask 3-ethynyl-10-octyl-10H-phenothiazine (**1**) (0.100 g, 0.30 mmol) and 4-bromo-7-(9H-carbazol-9-yl)benzo[c][1,2,5]thiadiazole (**5**) (0.136 g, 0.36 mmol) were dissolved in 1:1 (v/v) triethylamine (TEA; 30 mL) and tetrahydrofuran (THF; 30 mL). The reaction mixture was purged with argon, and $Pd(PPh_3)_4$ (0.017 g, 0.015 mmol), and CuI (0.006 g, 0.029 mmol) were added. The reaction mixture was reflux for 12 h. Upon the completion of the reaction, the mixture was evaporated and purified by silica gel column chromatography with hexane/CH_2Cl_2 (1:1) to get the desired compound **PT-CARs** as a red colored solid. Yield 74%; ^{1}H NMR (500 MHz, $CDCl_3$) δ: 8.17 (d, J = 7.6 Hz, 2 H), 7.94 (d, J = 7.5 Hz, 1 H), 7.81 (d, J = 7.5 Hz, 1 H), 7.52 - 7.44 (m, 2 H), 7.39 (t, J = 7.2 Hz, 2 H), 7.32 (t, J = 7.1 Hz, 2 H), 7.21 - 7.09 (m, 4 H), 6.94 (t, J = 7.2 Hz, 1 H), 6.85 (d, J = 8.5 Hz, 1 H), 6.87 (d, J = 8.7 Hz, 1 H), 3.86 (t, J = 7.1 Hz, 2 H), 1.82 (quin, J = 7.3 Hz, 2 H), 1.36 - 1.21 (m, 10 H), 0.88 (t, J = 6.3 Hz, 3 H); ^{13}C NMR (125 MHz, $CDCl_3$) δ: 156.0, 151.2, 146.2, 144.5, 141.0, 132.2, 131.4, 130.6, 129.8, 127.7, 127.5, 127.4, 126.0, 124.9, 124.1, 124.0, 122.9, 120.7, 120.5, 117.0, 116.0, 115.6, 115.1, 110.4, 96.8, 85.0, 47.7, 31.8, 29.7, 29.2, 29.2, 26.9, 26.8, 22.6, 14.1; HRMS (ESI-TOF) m/z $[M]^+$ calculated for $C_{40}H_{34}N_4S_2$ 634.2219, measured 634.2214.

Synthesis and characterization of 3-((7-((4-(9H-carbazol-9-yl)phenyl)ethynyl)benzo[c][1,2,5] thiadiazol-4-yl) ethynyl)-10-octyl-10H-phenothiazine (**PT-CAR**): In a 100 mL round bottomed flask 3-ethynyl-10-octyl-10H- phenothiazine (**1**) (0.100 g, 0.30 mmol) and 4-((4-(9H-carbazol-9-yl)phenyl)ethynyl)-7-bromobenzo[c][1,2,5] thiadiazole (**6**) (0.172 g, 0.36 mmol) were dissolved in 1:1 (v/v) triethylamine (TEA; 30 mL) and tetrahydrofuran (THF; 30 mL). The reaction mixture was purged with argon, and $Pd(PPh_3)_4$ (0.017 g, 0.015 mmol), and CuI (0.006 g, 0.029 mmol) were added. The reaction mixture was reflux for 12 h. Upon the completion of the reaction, the mixture was evaporated and purified by silica gel column chromatography with hexane/CH_2Cl_2 (1:1) to get the desired compound **PT-CAR** as a red colored solid. Yield 68%; ^{1}H NMR (500 MHz, $CDCl_3$) δ: 8.15 (d, J = 7.8 Hz, 2 H), 7.94 - 7.86 (m, J = 8.4 Hz, 2 H), 7.82 (d, J = 7.3 Hz, 1 H), 7.76 (d, J = 7.3 Hz, 1 H), 7.68 - 7.59 (m, J = 8.2 Hz, 2 H), 7.52 - 7.38 (m, 6 H), 7.31 (t, J = 6.9 Hz, 2 H), 7.18 - 7.10 (m, 2 H), 6.93 (t, J = 7.2 Hz, 1 H), 6.86 (d, J = 8.1 Hz, 1 H), 6.83 (d, J = 8.5 Hz, 1 H), 3.85 (t, J = 7.1 Hz, 2 H), 1.81 (quin, J = 7.2 Hz, 2 H), 1.30 - 1.23 (m, 10 H), 0.89 - 0.86 (m, 3 H); ^{13}C NMR (125 MHz, $CDCl_3$) δ: 154.4, 154.4, 146.2, 144.4, 140.5, 138.3, 133.5, 132.7, 132.0, 131.3, 130.5, 127.5, 127.4, 126.9, 126.1, 124.9, 124.1, 123.7, 122.9, 121.5, 120.4, 120.3, 117.7, 116.5, 116.0, 115.6, 115.0, 109.8, 97.7, 96.5, 86.3, 85.5, 47.7, 31.8, 29.7, 29.2, 29.2, 26.9, 26.8, 22.6, 14.1; HRMS (ESI-TOF) m/z $[M+H]^+$ calculated for $C_{48}H_{38}N_4S_2$ 735.2611, measured 735.2611.

Synthesis and characterization of 10-octyl-3-((7-((10-propyl-10H-phenothiazin-3-yl)ethynyl)benzo[c][1,2,5] thiadiazol-4-yl)ethynyl)-10H-phenothiazine (**PT-PT'**): In a 100 mL round bottomed flask 3-ethynyl-10-octyl-10H-phenothiazine (**1**) (0.100 g, 0.30 mmol) and 3-((7-bromobenzo[c][1,2,5]thiadiazol-4-yl)ethynyl)-10-propyl-10H-phenothiazine (**7**) (0.170 g, 0.36 mmol) were dissolved in 1:1 (v/v) triethylamine (TEA; 30 mL) and tetrahydrofuran (THF; 30 mL). The reaction mixture was purged with argon, and $Pd(PPh_3)_4$ (0.017 g, 0.015 mmol), and CuI (0.006 g, 0.029 mmol) were added. The reaction mixture was reflux for 12 h. Upon the completion of the reaction, the mixture was evaporated and purified by silica gel column chromatography with hexane/CH_2Cl_2

(1:1) to get the desired compound **PT-PT'** as a red colored solid. Yield 76%; ^{1}H NMR (500 MHz, $CDCl_3$) δ: 87.75 - 7.69 (m, 2 H), 7.43 (dd, J = 1.7, 8.4 Hz, 2 H), 7.40 (t, J = 1.5 Hz, 2 H), 7.17 - 7.11 (m, 4 H), 6.93 (t, J = 7.4 Hz, 2 H), 6.86 (d, J = 8.1 Hz, 2 H), 6.82 (d, J = 8.4 Hz, 2 H), 3.87 - 3.81 (m, 4 H), 1.87 - 1.77 (m, 4 H), 1.33 - 1.23 (m, 10 H), 1.02 (t, J = 7.3 Hz, 3 H), 0.89 - 0.85 (m, 3 H); ^{13}C NMR (125 MHz, $CDCl_3$) δ: 154.4, 146.1, 144.4, 132.1, 131.3, 130.5, 127.5, 127.4, 124.9, 124.8, 124.1, 124.1, 122.9, 122.9, 117.0, 116.2, 116.1, 115.6, 115.6, 115.0, 115.0, 97.3, 85.5, 49.4, 47.7, 31.7, 29.7, 29.2, 29.2, 26.9, 26.8, 22.6, 20.1, 14.1, 11.3; HRMS (ESI-TOF) m/z $[M+H]^+$ calculated for $C_{45}H_{40}N_4S_3$ 732.2410, measured 732.1968.

Synthesis and characterization of N,N-dimethyl-4-((7-((10-octyl-10H-phenothiazin-3-yl)ethynyl)benzo[c] [1,2,5] thiadiazol-4-yl)ethynyl)aniline (**PT-DMA**): In a 100 mL round bottomed flask 3-ethynyl-10-octyl-10H-phenothia zine (**1**) (0.100 g, 0.30 mmol) and 4-((7-bromobenzo[c][1,2,5]thiadiazol-4-yl)ethynyl)-N,N-dimethylaniline (**8**) (0.128 g, 0.36 mmol) were dissolved in 1:1 (v/v) triethylamine (TEA; 30 mL) and tetrahydrofuran (THF; 30 mL). The reaction mixture was purged with argon, and $Pd(PPh_3)_4$ (0.017 g, 0.015 mmol), and CuI (0.006 g, 0.029 mmol) were added. The reaction mixture was reflux for 12 h. Upon the completion of the reaction, the mixture was evaporated and purified by silica gel column chromatography with hexane/CH_2Cl_2 (1:1) to get the desired compound **PT-DMA** as a red colored solid. Yield 78%; ^{1}H NMR (500 MHz, $CDCl_3$) δ: 7.73 - 7.68 (m, 2 H), 7.56 - 7.53 (m, J = 8.7 Hz, 2 H), 7.43 (d, J = 8.4 Hz, 1 H), 7.40 (s, 1 H), 7.18 - 7.10 (m, 2 H), 6.95 - 6.91 (m, 1 H), 6.86 (d, J = 8.1 Hz, 1 H), 6.82 (d, J = 8.4 Hz, 1 H), 6.73 - 6.66 (m, J = 8.5 Hz, 2 H), 3.85 (t, J = 7.2 Hz, 2 H), 3.02 (s, 6 H), 1.81 (quin, J = 7.4 Hz, 2 H), 1.47 - 1.41 (m, 2 H), 1.33 - 1.23 (m, 8 H), 0.87 (t, J = 6.8 Hz, 3 H); ^{13}C NMR (125 MHz, CDCl3) δ: 154.4, 150.6, 145.9, 144.5, 133.3, 132.3, 131.5, 131.2, 130.5, 127.5, 127.3, 124.8, 124.1, 122.8, 117.9, 116.3, 116.1, 115.6, 115.0, 111.7, 109.1, 99.7, 96.8, 85.6, 84.2, 47.7, 40.2, 31.7, 29.2, 26.9, 26.8, 22.6, 14.1; HRMS (ESI-TOF) m/z $[M+H]^+$ calculated for $C_{38}H_{36}N_4S_2$ 613.2454, measured 613.2080.

Synthesis and characterization of 3,3'-((((10-octyl-10H-phenothiazine-3,7-diyl)bis(ethyne-2,1-diyl))bis(benzo [c][1,2,5]thiadiazole-7,4-diyl))bis(ethyne-2,1-diyl))bis(10-propyl-10H-phenothiazine) (**PT'-PT-PT'**): In a 100 mL round bottomed flask 3,7-diethynyl-10-octyl-10H-phenothiazine (**2**) (0.10 g, 0.27 mmol) and 3-((7-bromobenzo [c][1,2,5]thiadiazol-4-yl)ethynyl)-10-propyl-10H-phenothiazine (**7**) (0.21 g, 0.45 mmol) were dissolved in 1:1 (v/v) triethylamine (TEA; 30 mL) and tetrahydrofuran (THF; 30 mL). The reaction mixture was purged with argon, and $Pd(PPh_3)_4$ (0.032 g, 0.027 mmol), and CuI (0.010 g, 0.054 mmol) were added. The reaction mixture was reflux for 12 h. Upon the completion of the reaction, the mixture was evaporated and purified by silica gel column chromatography with hexane/CH_2Cl_2 (1:1) to get the desired compound **PT'-PT-PT'** as a red colored solid. Yield 56%; ^{1}H NMR (400 MHz, $CDCl_3$): δ 7.94 (d, 2H), 7.78 (d, 2H), 7.49 (d, 2H), 7.45 (s, 2H), 7.07 (d, 4H), 6.87 (d, 2H), 6.80 (d, 4H), 6.77 (d, 4H), 6.03 (d, 4H), 3.91(t, 2H), 1.87-1.83 (m, 2H), 1.30-1.26 (m,), 0.89 (t, 3H); ^{13}C NMR (400 MHz, $CDCl_3$): δ: 154.34, 146.04, 144.39, 132.17, 132.07, 131.30, 127.50, 124.83, 124.05, 122.88, 116.13, 115.62, 115.22, 49.36, 47.85, 31.74, 29.72, 29.27, 26.92, 22.64, 20.09, 14.13, 11.29 ; MALDI-TOF-MS: m/z: calcd for $C_{70}H_{55}N_7S_5$ 1153.3122 $[M]^+$, measured 1153.4545.

Synthesis and characterization of 4,4'-((((10-octyl-10H-phenothiazine-3,7-diyl)bis(ethyne-2,1-diyl))bis(ben zo [c][1,2,5]thiadiazole-7,4-diyl))bis(ethyne-2,1-diyl))bis(N,N-dimethylaniline) (**DMA-PT-DMA**): In a 100 mL round bottomed flask 3,7-diethynyl-10-octyl-10H-phenothiazine (**2**) (0.10 g, 0.27 mmol) and 4-((7-bromobenzo[c] [1,2,5] thiadiazol-4-yl)ethynyl)-N,N-dimethylaniline (**8**) (0.23 g, 0.64 mmol) were dissolved in 1:1 (v/v) triethylamine (TEA; 30 mL) and tetrahydrofuran (THF; 30 mL). The reaction mixture was purged with argon, and $Pd(PPh_3)_4$ (0.032 g, 0.027 mmol), and CuI (0.010 g, 0.057 mmol) were added. The reaction mixture was reflux for 12 h. Upon the completion of the reaction, the mixture was evaporated and purified by silica gel column chromatography with hexane/CH_2Cl_2 (1:1) to get the desired compound **DMA-PT-DMA** as a red colored solid. Yield 52%; ^{1}H NMR (400 MHz, $CDCl_3$): δ 7.72 (d, 4H), 7.54 (d, 4H), 7.44 (d, 2H), 7.40 (s, 2H), 6.83 (d, 2H), 6.68 (d, 2H), 3.81 (t, 2H), 3.03 (s, 12H), 1.97-1.94 (m, 2H), 1.35-1.31 (m, 10H), 0.90 (t, 3H); ^{13}C NMR (400 MHz, $CDCl_3$): δ: 156.12, 152.90, 143.38, 135.01, 134.00, 133.39, 132.27, 130.50, 126.98, 123.18, 121.07, 116.06, 53.44, 48.99, 31.72, 29.19, 26.87, 26.66, 22.62, 14.10 ; HRMS (ESI-TOF) m/z $[M]^+$ calculated

for $C_{56}H_{47}N_7S_3$ 914.3128, measured 914.2318.

Synthesis and characterization of 3-((7-((4-(dimethylamino)phenyl)ethynyl)benzo [c][1,2,5]thiadiazol-4-yl) ethynyl)-10-octyl-10H-phenothiazine 5,5-dioxide (**PTO-DMA**): In a 100 mL round bottomed flask 3-ethynyl-10-octyl-10H-phenothiazine 5,5-dioxide (**3**) (0.100 g, 0.27 mmol) and 4-((7-bromobenzo[c][1,2,5]thiadiazol-4-yl)ethynyl)-N,N-dimethylaniline (**8**) (0.117 g, 0.33 mmol) were dissolved in 1:1 (v/v) triethylamine (TEA; 30 mL) and tetrahydrofuran (THF; 30 mL). The reaction mixture was purged with argon, and $Pd(PPh_3)_4$ (0.017 g, 0.015 mmol), and CuI (0.006 g, 0.029 mmol) were added. The reaction mixture was reflux for 12 h. Upon the completion of the reaction, the mixture was evaporated and purified by silica gel column chromatography with hexane/CH_2Cl_2 (1:1) to get the desired compound **PTO-DMA** as a red colored solid. Yield 72%; ^1H NMR (500 MHz, $CDCl_3$) δ: 8.41 (s, 1 H), 8.14 (d, J = 7.9 Hz, 1 H), 7.86 (d, J = 8.7 Hz, 1 H), 7.78 (d, J = 7.5 Hz, 1 H), 7.73 (d, J = 7.5 Hz, 1 H), 7.65 (t, J = 7.9 Hz, 1 H), 7.55 (d, J = 8.7 Hz, 2 H), 7.38 - 7.34 (m, 1 H), 7.33 - 7.30 (m, 1 H), 7.21 - 7.14 (m, 1 H), 6.69 (d, J = 8.7 Hz, 2 H), 4.20 - 4.13 (m, 2 H), 3.03 (s, 5 H), 2.99 (s, 1 H), 1.98 - 1.90 (m, 2 H), 1.52 - 1.45 (m, 2 H), 1.41 - 1.36 (m, 2 H), 1.35 - 1.28 (m, 6 H), 0.90 (t, J = 6.7 Hz, 3 H); ^{13}C NMR (125 MHz, $CDCl_3$) δ: 154.4, 150.6, 140.5, 140.4, 136.1, 134.4, 133.4, 133.3, 132.9, 131.3, 127.5, 124.5, 124.3, 123.8, 122.3, 118.6, 116.3, 116.1, 115.4, 111.7, 109.0, 100.2, 95.0, 86.8, 84.2, 48.7, 40.2, 31.7, 30.3, 29.7, 29.2, 29.2, 26.8, 26.7, 22.6, 14.1; HRMS (ESI-TOF) m/z $[M+H]^+$ calculated for $C_{38}H_{36}N_4O_2S_2$ 644.2280, measured 644.8520.

Synthesis and characterization of 3,7-bis((7-((4-(dimethylamino)phenyl)ethynyl)benzo [c][1,2,5]thiadiazol-4-yl)ethynyl)-10-octyl-10H-phenothiazine 5,5-dioxide (**DMA-PTO-DMA**): In a 100 mL round bottomed flask 3,7-diethynyl-10-octyl-10H-phenothiazine 5,5-dioxide (**4**) (0.10 g, 0.23 mmol) and 4-((7-bromobenzo[c][1,2,5]thia diazol-4-yl)ethynyl)-N,N-dimethylaniline (**8**) (0.190 g, 0.54 mmol) were dissolved in 1:1 (v/v) triethylamine (TEA; 30 mL) and tetrahydrofuran (THF; 30 mL). The reaction mixture was purged with argon, and $Pd(PPh_3)_4$ (0.029 g, 0.025 mmol), and CuI (0.050 g, 0.078 mmol) were added. The reaction mixture was reflux for 12 h. Upon the completion of the reaction, the mixture was evaporated and purified by silica gel column chromatography with hexane/CH_2Cl_2 (1:4) to get the desired compound **DMA-PTO-DMA** as a colored solid. Yield 68%; ^1H NMR (400 MHz, $CDCl_3$): δ 8.28 (s, 2H), 7.88 (d, 2H), 7.79 (d, 2H), 7.75 (d, 2H), 6.83 (d, 4H), 6.68 (d, 4H), 4.24 (t, 2H), 3.03 (s, 12H), 1.97-1.94 (m, 2H), 1.35-1.31 (m, 10H), 0.90 (t, 3H); ^{13}C NMR (400 MHz, $CDCl_3$): δ: 156.12, 152.90, 143.38, 135.01, 134.00, 133.39, 132.27, 130.50, 126.98, 123.18, 121.07, 116.06, 53.44, 48.99, 31.72, 29.19, 26.87, 26.66, 22.62, 14.10 ; HRMS (ESI-TOF) m/z $[M]^+$ calculated for $C_{56}H_{47}N_7O_2S_3$ 963.3292, measured 963.4391.

S1.2 Stationary electronic spectroscopy

The absorption spectra of the sample solutions (ca. 10^{-5} M) were recorded using a Cary 4E (Varian) spectrophotometer. Fluorescence and excitation spectra were recorded with an FS5 spectrofluorometer from Edinburgh Instruments with the appropriate instrumental response corrections. The fluorescence quantum yields (Φ_{fl}, experimental error $\pm$10%) of dilute solutions (ca. 10^{-6} M) were obtained by employing tetracene ($\Phi_{fl} = 0.17$ in air-equilibrated cyclohexane) as reference.

S1.3 Time-resolved fluorescence

Fluorescence lifetimes were measured using the time-correlated single-photon counting (TC-SPC) method using an Edinburgh Instrument FS5 spectrofluorometer, equipped with a LED source centered at 375 nm, with a 0.2 ns temporal resolution.

S1.4 Electronic transient absorption spectroscopy

The experimental setup for the femtosecond transient absorption was based on a Helios system (Ultrafast Systems) as described before.[1,2] Excitation was carried out with 400 nm pulses, produced by frequency doubling part of the 800 nm output pulses of an amplified Ti:Sapphire laser system (ca. 60 fs, Spectra Physics) using an Apollo 2nd and 3rd harmonic generator. Probing was achieved with a white-light continuum (450-800 nm) produced by focusing a small fraction of the fundamental laser pulses onto a Sapphire crystal (2 mm thick) after passing through an optical delay line (time window of 3200 ps). The temporal resolution was about 150 fs, whereas the spectral resolution was 1.5 nm. The measurements were carried out at magic angle condition in a 2 mm cell. The samples had an absorbance at 400 nm between 0.5 and 1. The solutions were stirred during the measurements to avoid photoproduct interferences. The absence of relevant photodegradation was checked by recording the absorption spectra before and after measurements, and no significant change was observed.

S1.5 Time-resolved IR spectroscopy

Femtosecond time-resolved IR (TRIR) spectra were obtained using a homebuilt setup based on a Ti:Sapphire amplified system (Spectra Physics Solstice) generating 100 fs pulses at 800 nm and 1 kHz repetition rate as described in detail previously.[3,4] excitation was carried out either at 400 nm by frequency doubling a fraction of the amplifier output or at 532 nm using a TOPAS-Prime combined with a NirUVis module (Light Conversion). The linearity of the signal amplitude with respect to pump intensity was checked before each experiment and proper adjustment was made to ensure the maximum signal in a linear regime. The polarization was controlled with a combination of Glan-Laser polariser and zero-order half-wave plate, limiting the time resolution of the experiment to 300 fs. The pulses were focused on the sample onto 350 μm spot, resulting in a fluence of 0.05-0.3 mJ/cm^2. Mid-IR probe pulses at around 4.7-5.2 μm where generated by difference frequency mixing of the output of an optical parametric amplifier (Light Conversion, TOPAS-C with NDFG module) that was pumped at 800 nm. The polarization of the IR beam was controlled using a wire-grid polarizer. Two horizontally polarized IR beams were produced with a CaF2 wedge and focused onto the sample. One of the beams was overlapped with the pump beam, whereas the second was used as reference. Both IR beams were focused onto the entrance slit of an imaging spectrograph (Horiba, Triax 190, 150 lines/mm) equipped with a liquid nitrogen cooled 2 x 64 element MCT array (Infrared Systems Development), giving a resolution of 3-4 cm^{-1} in the -C≡C- stretching region. The sample area and the detection system were placed in a box that was purged with water- and carbon dioxide-free air for at least one hour before each experiment. The average of 500 signal shots was taken to collect one data point with the polarization of the pump pulses at the magic angle with respect to that of the IR pulse. This procedure was carried out for at least four times depending on the signal reproducibility and intensity.

S1.6 Quantum-chemical calculations

All calculations were carried out in the gas phase at the density functional theory (DFT) or time-dependent (TD) DFT levels using the CAM-B3LYP functional,[5] and the 6-31g(d,p) basis set, as implemented in Gaussian16 (Rev. B).[6] To speed up the calculations, the octyl substituent on the phenothiazine N atom was replaced by a methyl group.

S1.7 Molecular dynamics simulations

Molecular dynamics (MD) simulations were carried out using GROMACS 2023.1.[7] The optimised geometry of **CAR-PT-CAR** was determined from quantum-chemical calculations in the gas phase at the DFT level (B3LYP/6-31G+d) using Gaussian 16.[6] The topology files were generated using the Antechamber Python parser interface (ACPYPE)[8] with the general Amber force field (GAFF).[9] The atomic charges were determined from CHELPG fits of the electrostatic potential obtained from the quantum-chemical calculations.[10] The GAFF-ESP-2018 force field was used for the solvent.[11] Non-bonded interactions were evaluated with a cutoff of 1.2 nm, and long-range electrostatic interactions were accounted for by the particle mesh Ewald method,[12] with 0.16 nm grid spacing and forth-order interpolation. A long-range dispersion correction for energy was also included. The LINCS algorithm[13] was used to constrain the bonds of all system components. The equilibration of the system was ensured by inspecting the total energy drift. The isothermal-isobaric ensemble, NPT, was used for all productions with the Noose-Hoover thermostat at 295 K,[14] and the c-rescale barostat[15] at 1 atm using coupling constants 0.5 and 5 ps respectively.

A periodic cubic box (5x5x5 nm^3) filled with 1000 molecules of DMSO and one **CAR-PT-CAR** dye was used for the simulations, which were performed at constant pressure (1 atm) and temperature (295 K) with 2 fs steps for 25 to 100 ns.

S2 Additional results

S2.1 Stationary electronic spectroscopy

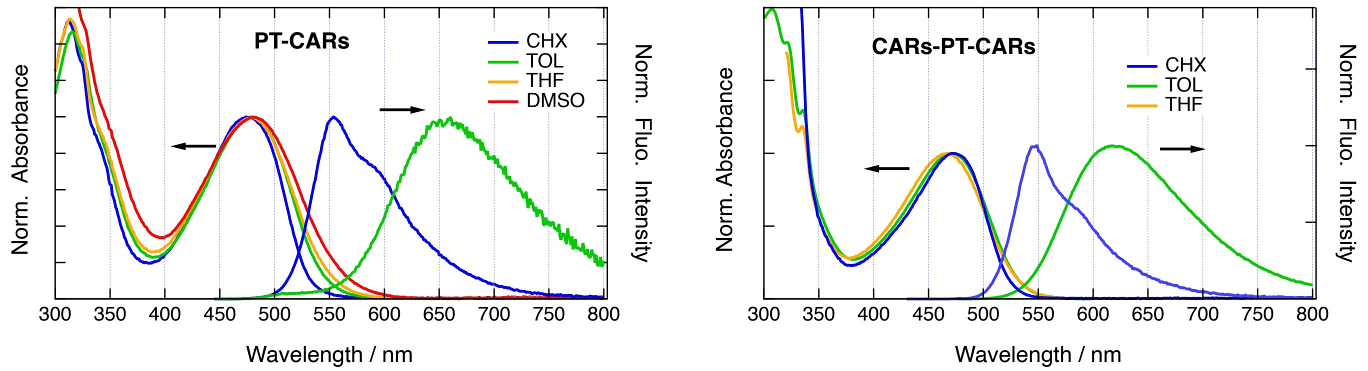

Figure S1 Stationary electronic absorption and fluorescene spectra of **PT-CARs** and **CARs-PT-CARs** in various solvents. CHX: cyclohexane; TOL: toluene; THF: tetrahydrofuran; DMSO: dimethylsulfoxide.

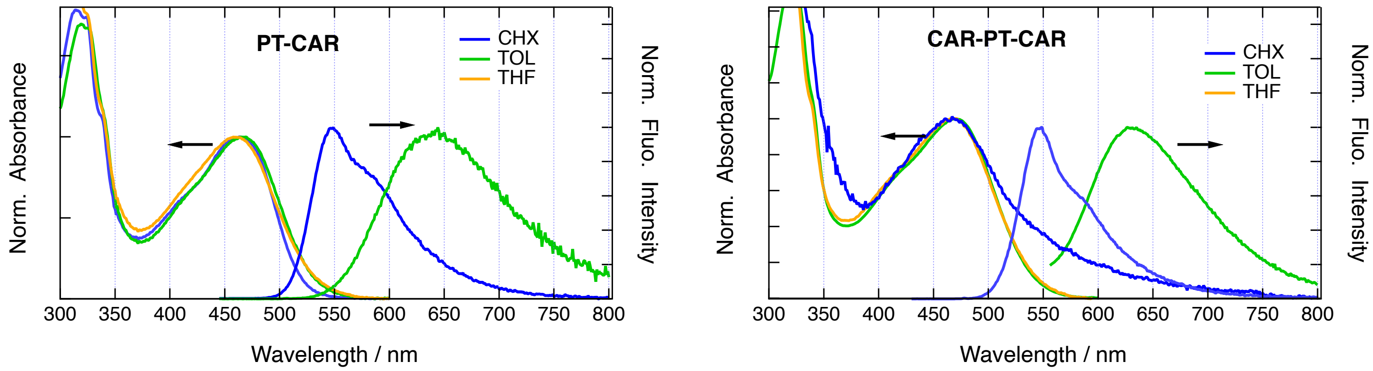

Figure S2 Stationary electronic absorption and fluorescene spectra of **PT-CAR** and **CAR-PT-CAR** in various solvents. The long-wavelength tail of the spectrum of **CAR-PT-CAR** in CHX point to the formation of aggregates.

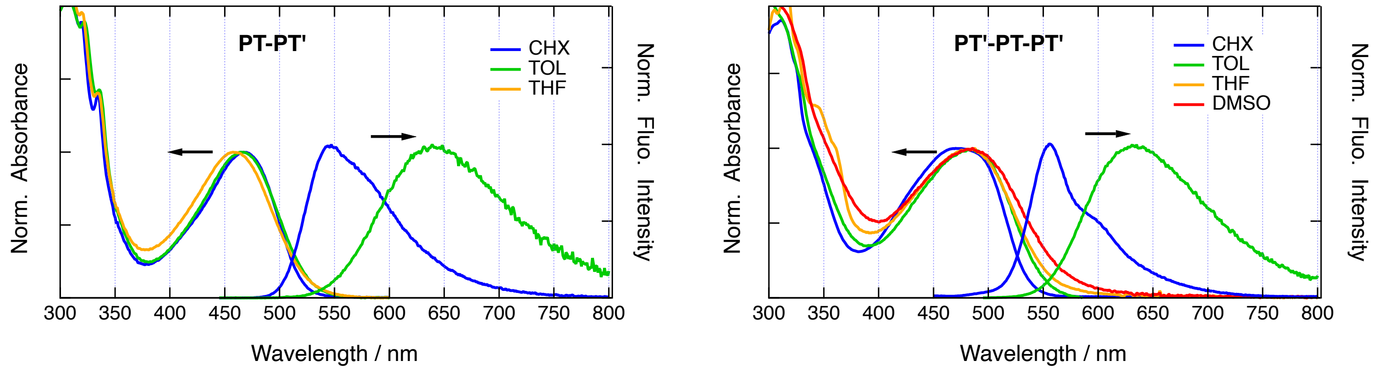

Figure S3 Stationary electronic absorption and fluorescene spectra of **PT-PT’** and **PT’-PT-PT’** in various solvents.

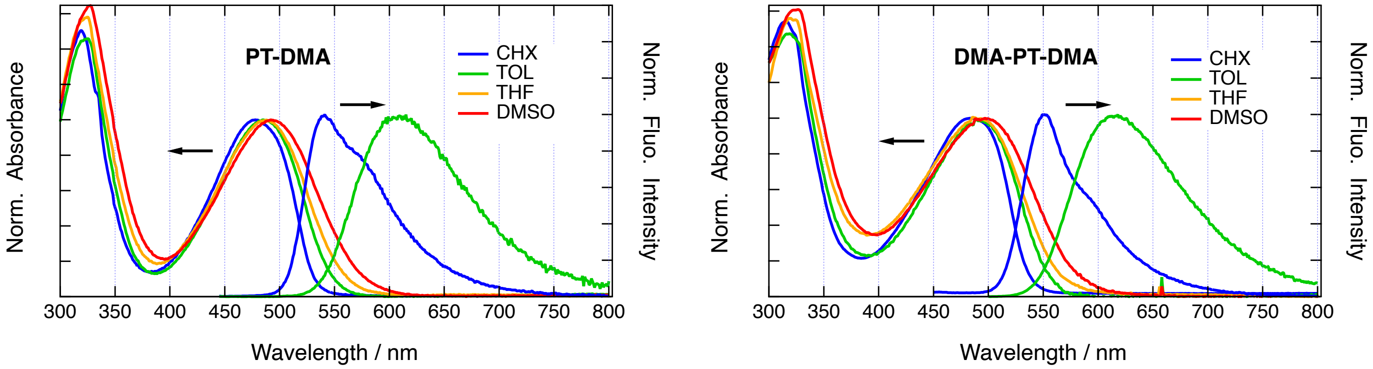

Figure S4 Stationary electronic absorption and fluorescene spectra of **PT-DMA** and **DMA-PT-DMA** in various solvents.

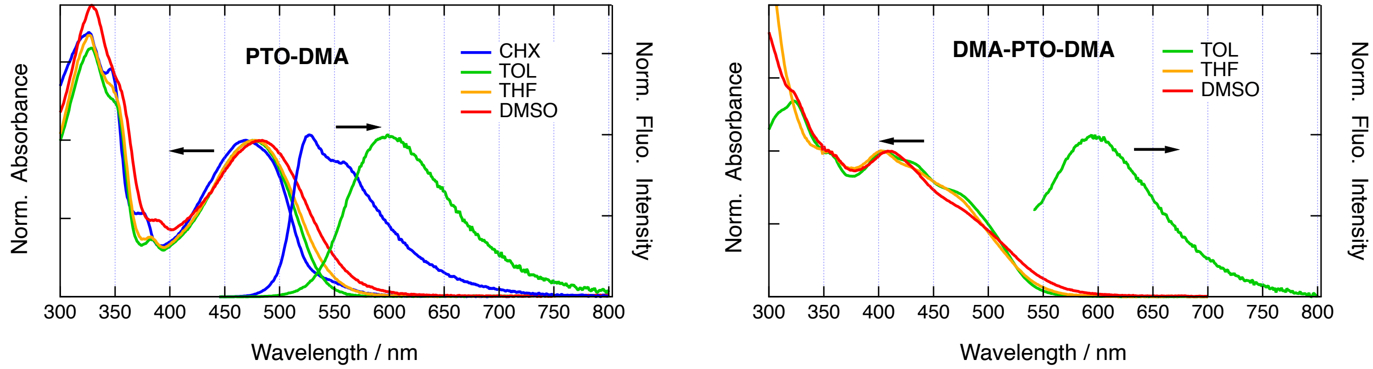

Figure S5 Stationary electronic absorption and fluorescene spectra of **PTO-DMA** and **DMA-PTO-DMA** in various solvents.

Table S1 Fluorescence quantum yields, Φ_{fl}, and lifetimes. τ_{fl}, of the dyes in various solvents. Error on τ_{fl}: $\pm 5\%$.

Dye	solvent	Φ_{fl}	τ_{fl} / ns
**PT-CARs**	CHX	0.59	5.6
	TOL	0.23	4.1
**CARs-PT-CARs**	TOL	0.16	
**PT-CAR**	CHX	0.65	4.0
	TOL	0.30	3.7
**CAR-PT-CAR**	TOL	0.20	
**PT-PT'**	CHX	0.54	3.3
	TOL	0.32	4.3
**PT'-PT-PT'**	CHX	0.51	2.6
	TOL	0.42	3.8
**PT-DMA**	CHX	0.55	3.3
	TOL	0.44	4.2
**DMA-PT-DMA**	CHX	0.32	2.6
	TOL	0.39	3.5
**PTO-DMA**	CHX	0.73	3.3
	TOL	0.57	4.2
**DMA-PTO-DMA**	TOL	0.98	3.8

S2.2 Stationary vibrational spectroscopy

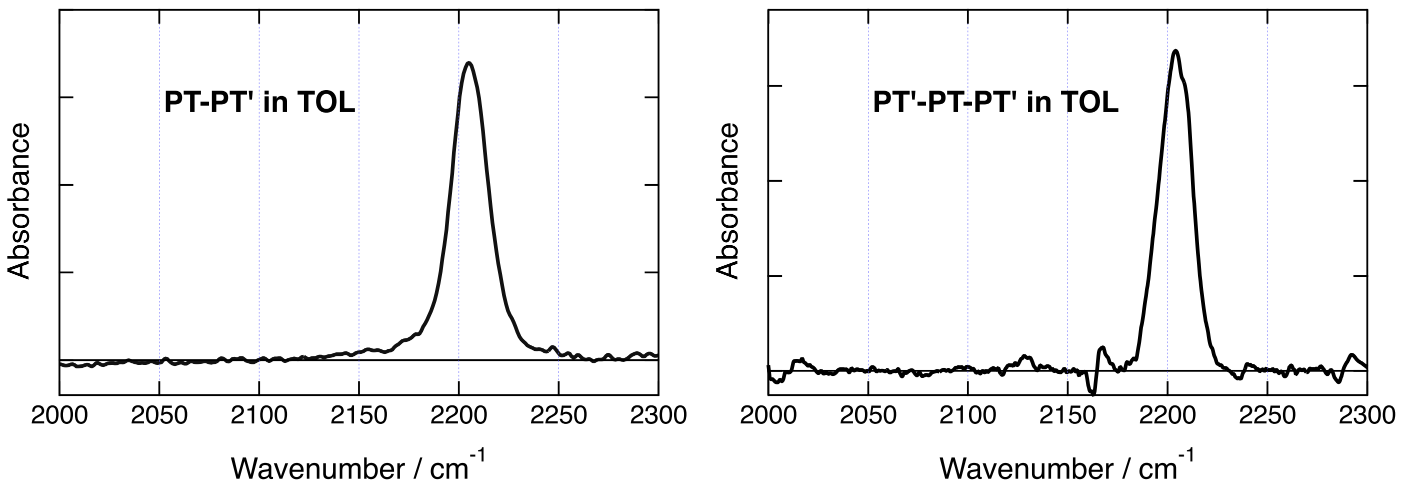

Figure S6 Stationary IR absorption spectra of **PT-PT'** and **PT'-PT-PT'** in toluene.

S2.3 Quantum-chemical calculations

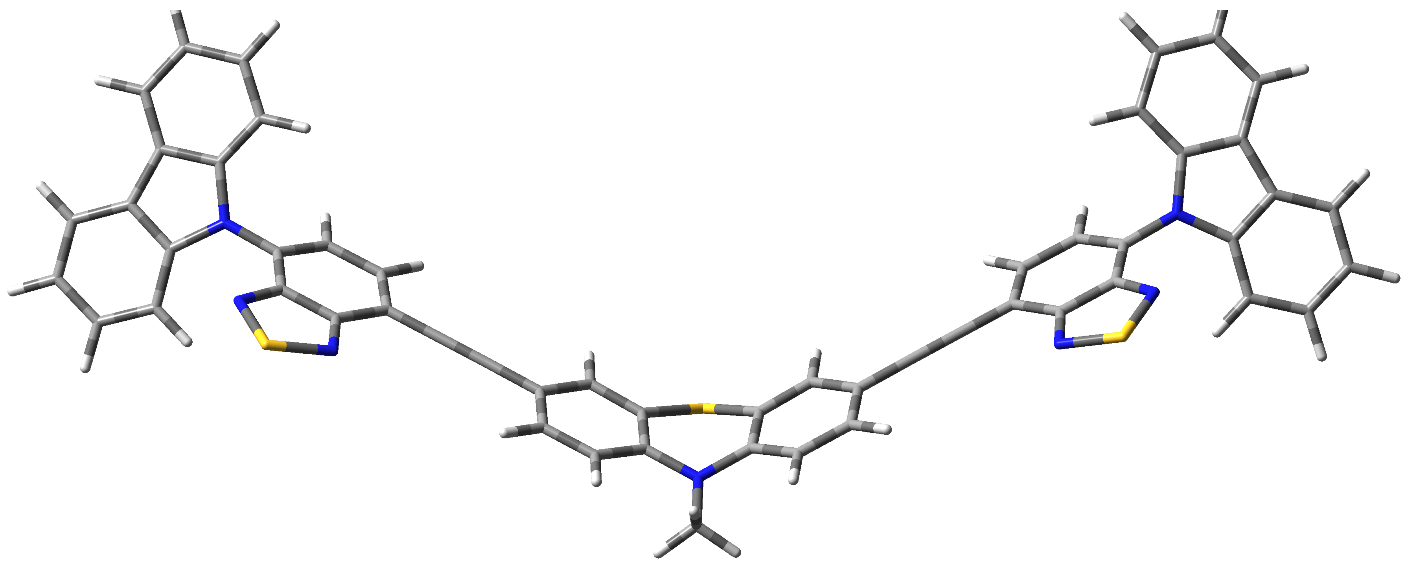

Figure S7 Ground-state optimised geometry of **CARs-PT-CARs**.

CAR-PT-CAR

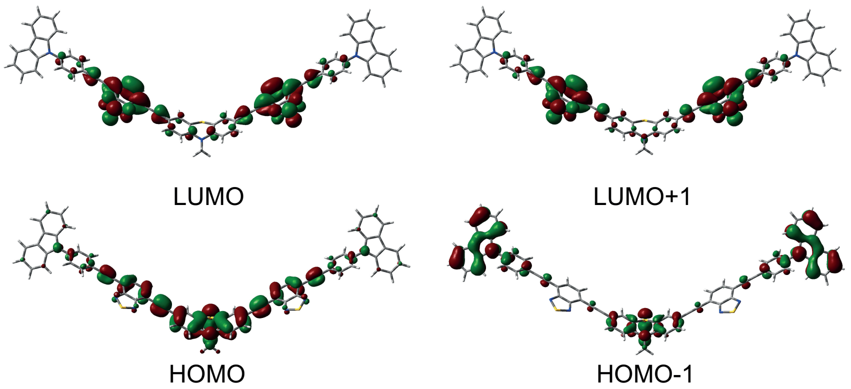

$S_1 \leftarrow S_0$: HOMO→LUMO + HOMO-1→LUMO+1, f = 2.6
$S_2 \leftarrow S_0$: HOMO→LUMO+1 + HOMO-1→LUMO, f = 0.6

Figure S8 Frontier molecular orbitals involved in the first two electronic transitions of **CAR-PT-CAR** and associated oscillator strength.

PT'-PT-PT'

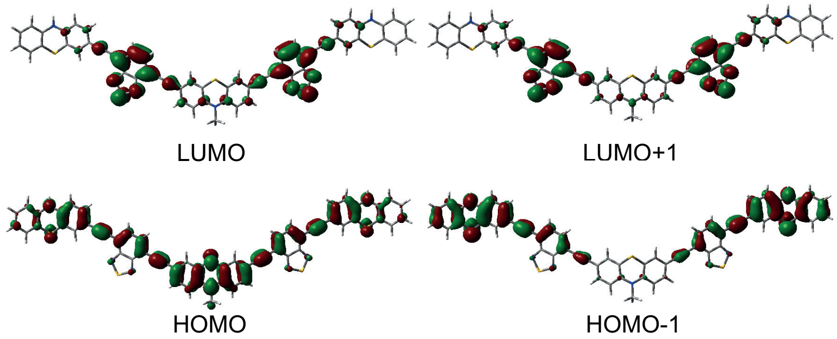

$S_1 \leftarrow S_0$: HOMO→LUMO + HOMO-1→LUMO+1, f = 2.6
$S_2 \leftarrow S_0$: HOMO→LUMO+1 + HOMO-1→LUMO, f = 0.49

Figure S9 Frontier molecular orbitals involved in the first two electronic transitions of **PT'-PT-PT'** and associated oscillator strength.

DMA-PT-DMA

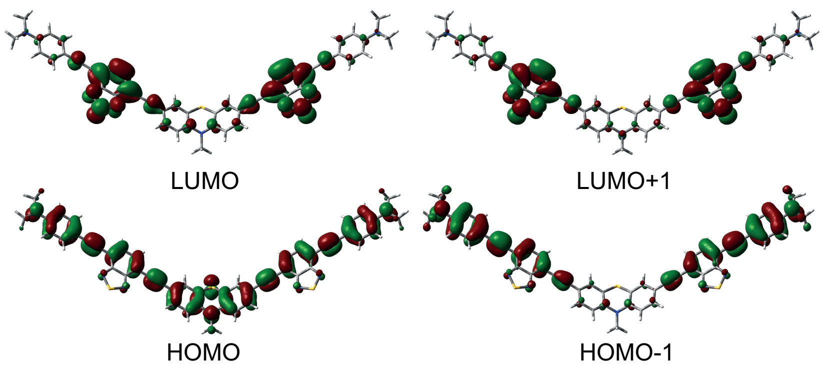

$S_1 \leftarrow S_0$: HOMO→LUMO + HOMO-1→LUMO+1, f = 2.3
$S_2 \leftarrow S_0$: HOMO→LUMO+1 + HOMO-1→LUMO, f = 0.56

Figure S10 Frontier molecular orbitals involved in the first two electronic transitions of **DMA-PT-DMA** and associated oscillator strength.

Table S2 Unscaled calculated -C≡C- stretching frequencies (in cm^{-1}) and IR intensity (in brackets, in km/mol) of the trans and cis conformers of **PT-CAR** in the S_0 and S_1 states.

Vibration	trans S_0	cis S_0	trans S_1	cis S_1
symmetric -C≡C-	2367 (121)	2366 (121)	2287 (440)	2287 (390)
antisym. -C≡C-	2375 (216)	2375 (212)	2232 (6100)	2231 (6050)

Table S3 S_2-S_1 energy gap of the 2B dyes obtained from TD-DFT calculations.

**Dye**	S_2-S_1 gap / eV
**CARs-PT-CARs**	0.08
**CAR-PT-CAR**	0.15
**PT'-PT-PT'**	0.13
**DMA-PT-DMA**	0.11
**DMA-PTO-DMA**	0.11

S2.4 Electronic transient absorption spectroscopy

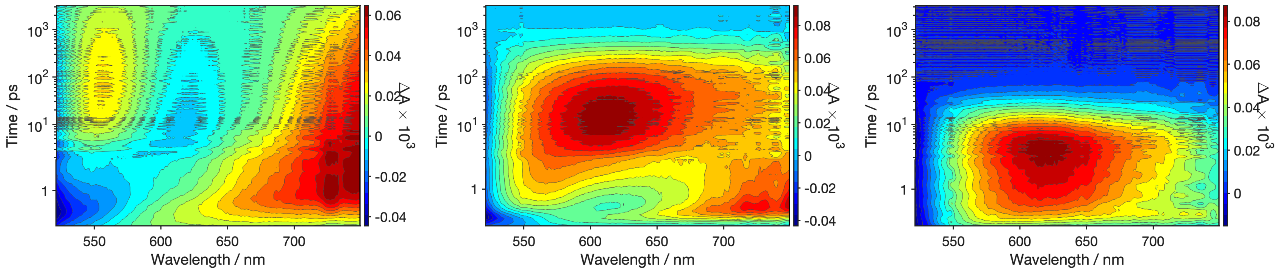

Figure S11 Transient electronic absorption recorded upon 400 nm excitation of **PT-DMA** in TOL (left), THF (middle), and DMSO (right).

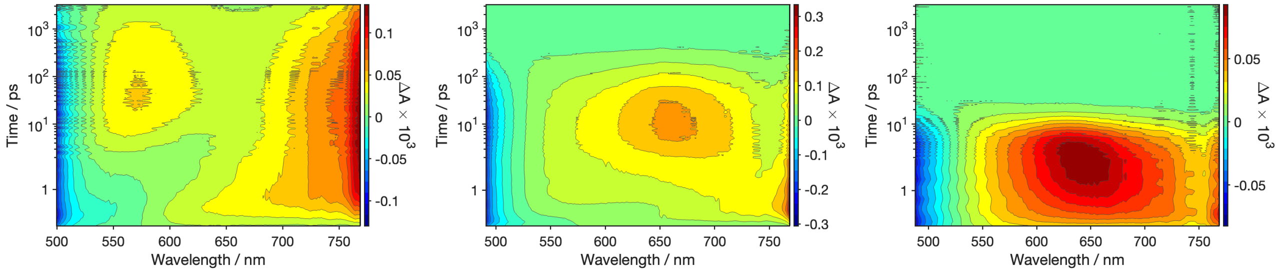

Figure S12 Transient electronic absorption recorded upon 400 nm excitation of **PT-PT'** in TOL (left), THF (middle), and DMSO (right).

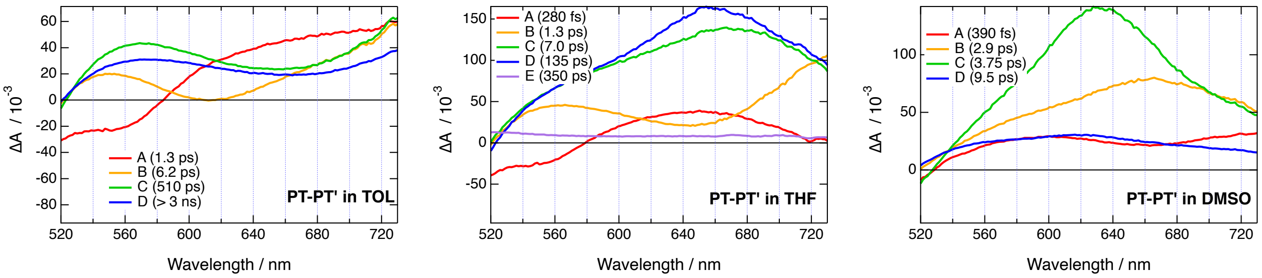

Figure S13 Evolution-associated difference absorption spectra and time constants obtained from a global analysis of the data shown in Figure S12 assuming a series of successive exponential steps.

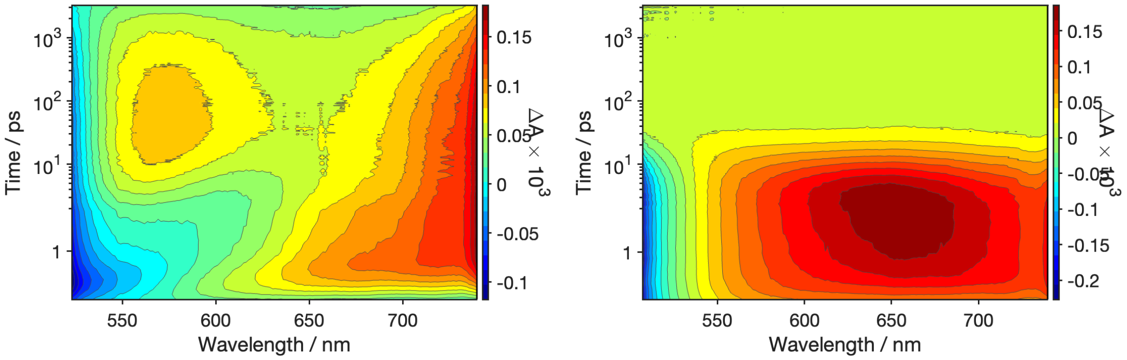

Figure S14 Transient electronic absorption recorded upon 400 nm excitation of **PT’-PT-PT’** in TOL (left) and DMSO (right).

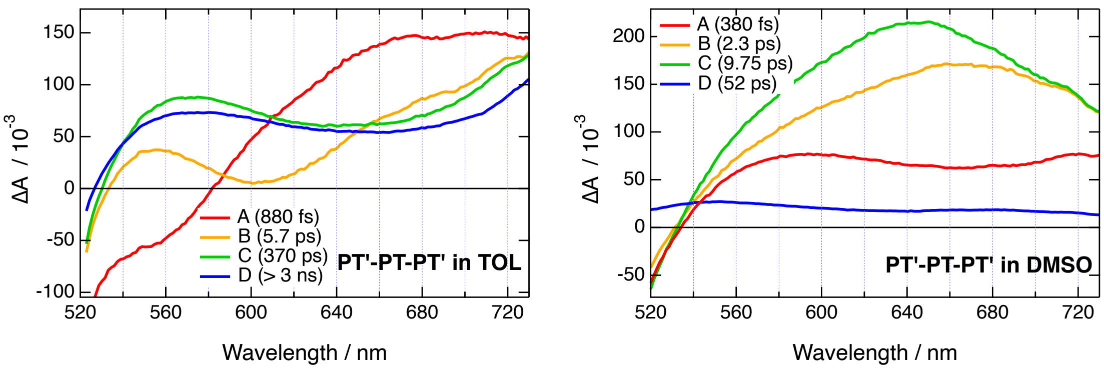

Figure S15 Evolution-associated difference absorption spectra and time constants obtained from a global analysis of the data shown in Figure S14 assuming a series of successive exponential steps.

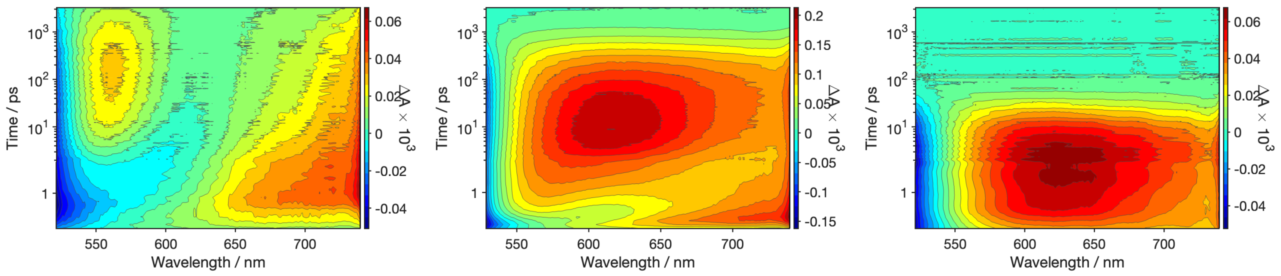

Figure S16 Transient electronic absorption recorded upon 400 nm excitation of **DMA-PT-DMA** in TOL (left), THF (middle), and DMSO (right).

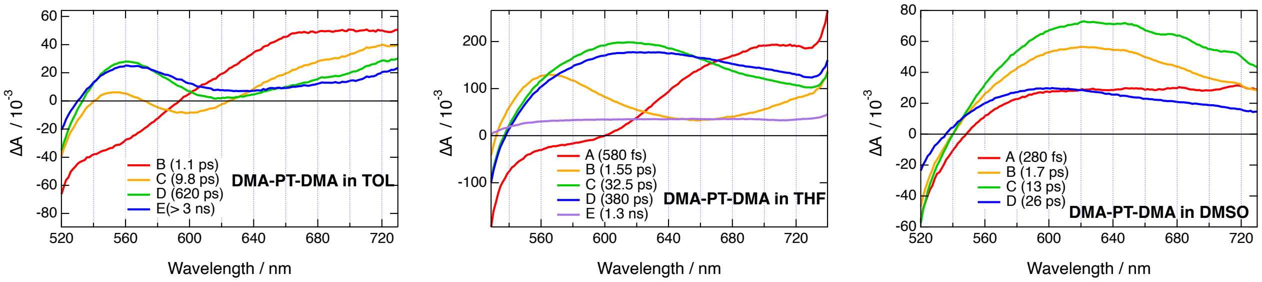

Figure S17 Evolution-associated difference absorption spectra and time constants obtained from a global analysis of the data shown in Figure S16 assuming a series of successive exponential steps.

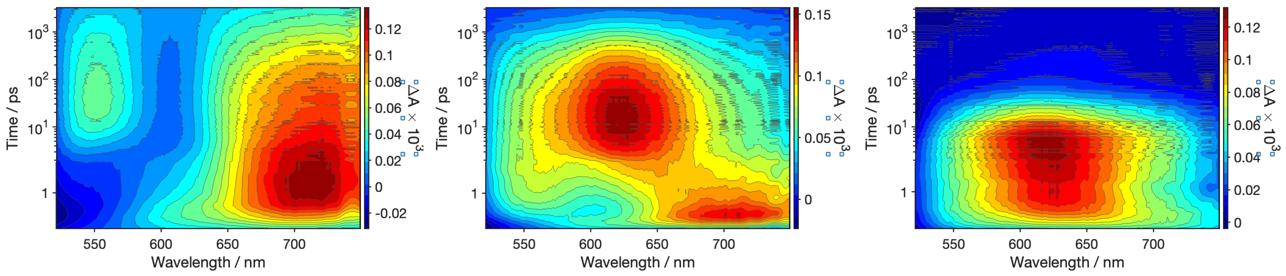

Figure S18 Transient electronic absorption recorded upon 400 nm excitation of **PTO-DMA** in TOL (left), THF (middle), and DMSO (right).

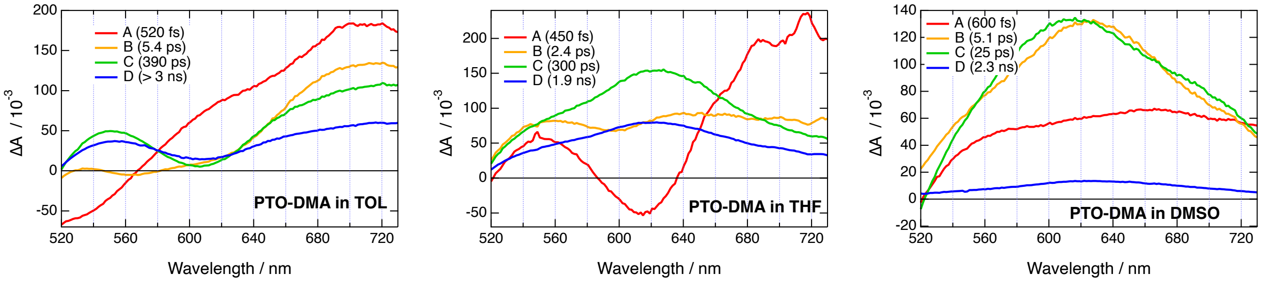

Figure S19 Evolution-associated difference absorption spectra and time constants obtained from a global analysis of the data shown in Figure S18 assuming a series of successive exponential steps.

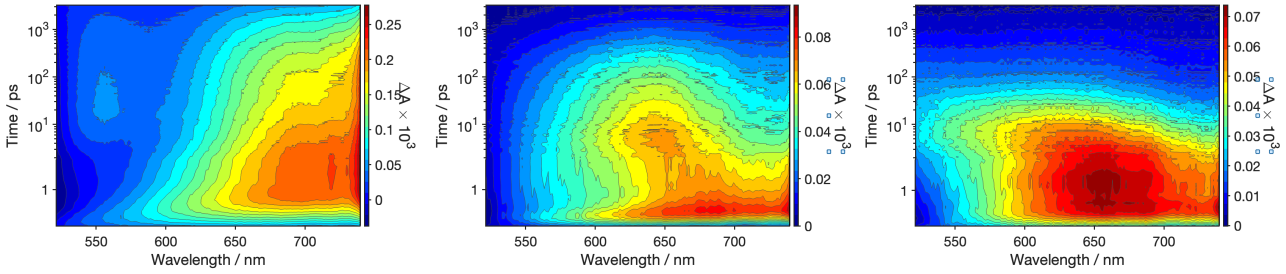

Figure S20 Transient electronic absorption recorded upon 400 nm excitation of **DMA-PTO-DMA** in TOL (left), THF (middle), and DMSO (right).

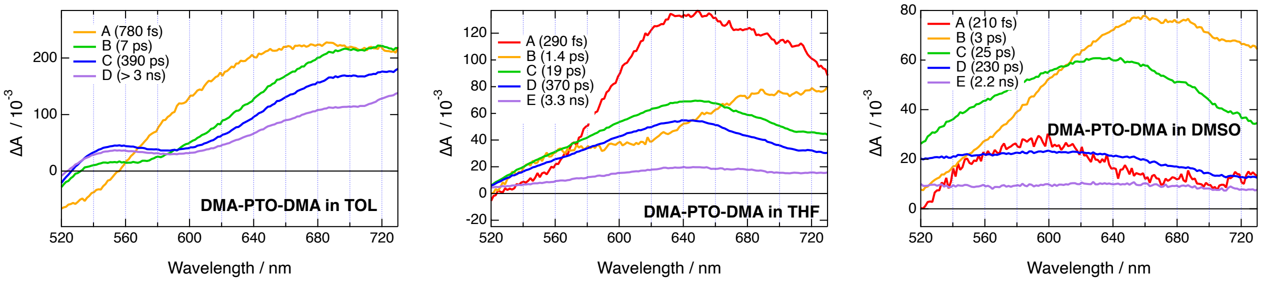

Figure S21 Evolution-associated difference absorption spectra and time constants obtained from a global analysis of the data shown in Figure S20 assuming a series of successive exponential steps.

S2.5 Time-resolved IR spectroscopy

PT-CARs

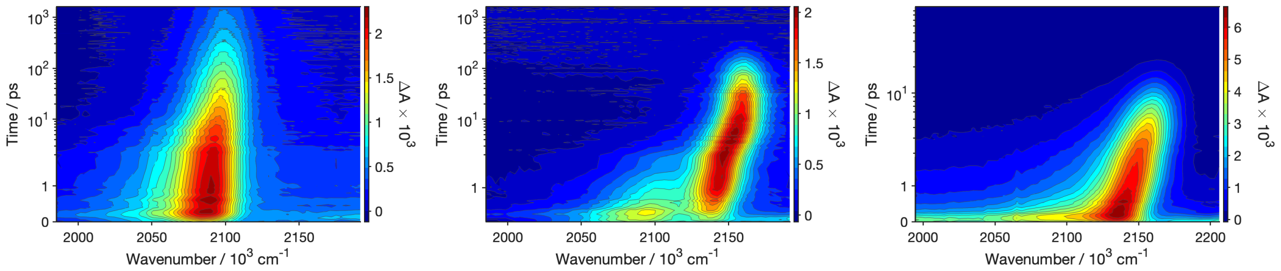

Figure S22 Transient IR absorption recorded upon 400 nm excitation of **PT-CARs** in CHX (left), THF (centre), and DMSO (right).

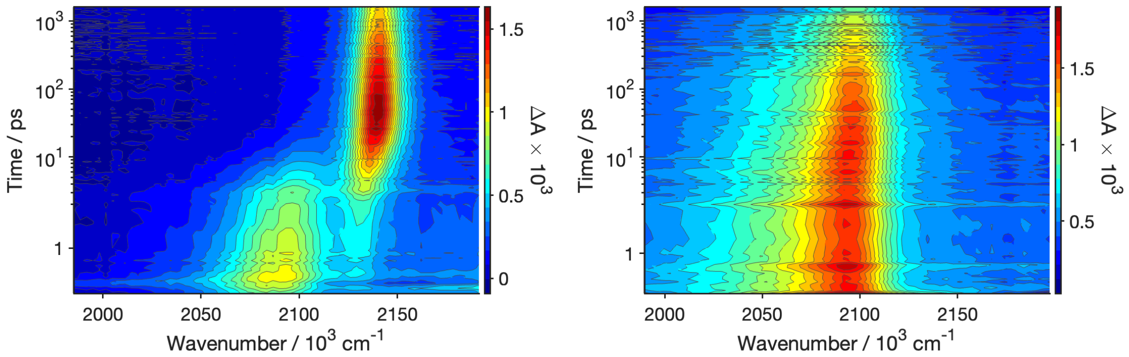

Figure S23 Transient IR absorption recorded with **PT-CARs** in TOL upon 400 nm excitation (left) and in CHX upon 530 nm excitation (right).

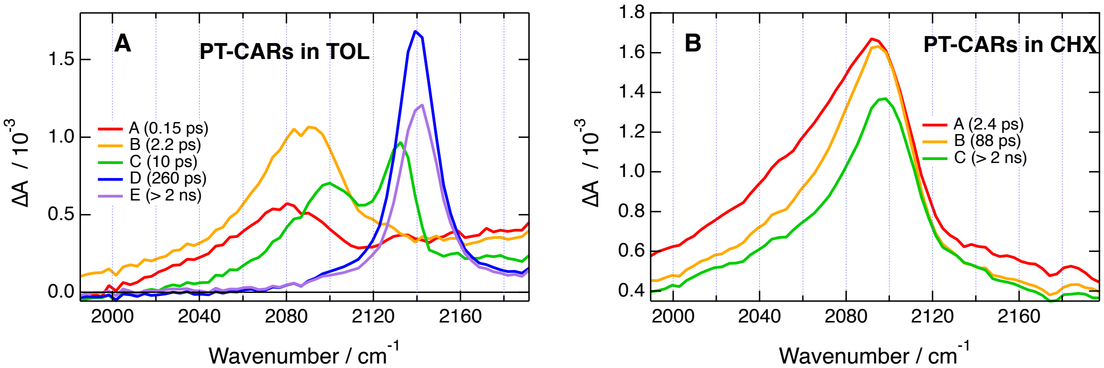

Figure S24 Evolution-associated difference absorption spectra and time constants obtained from a global analysis of the TRIR data shown in Figure S23 assuming a series of successive exponential steps.

PT-CAR

In CHX, the single -C≡C- band after the initial up-shift is located at 2130 cm^{-1} compared to 2100 cm^{-1} with **PT-CARs** (Figures S25 and S26). As solvent polarity increases, the amplitude of the early frequency up-shift becomes larger and, in the most polar DMSO, the band moves to 2160 cm^{-1} like with **PT-CARs**. The presence of a single -C≡C- band in the TRIR spectra of **PT-CAR** can be explained by a localisation of the excitation on either end of the molecule or by a fully delocalised excitation considering that only the antisymmetric -C≡C- stretch has significant IR intensity. The 30 cm^{-1} frequency up-shift of the band in CHX compared to **PT-CARs** is consistent with a delocalised excitation and a vibration involving the antisymmetric stretch of both -C≡C- groups. By contrast, the similar position of the band in DMSO rather agrees with a localisation of the excitation on the PT-BTD end of the molecule like for **PT-CARs**.

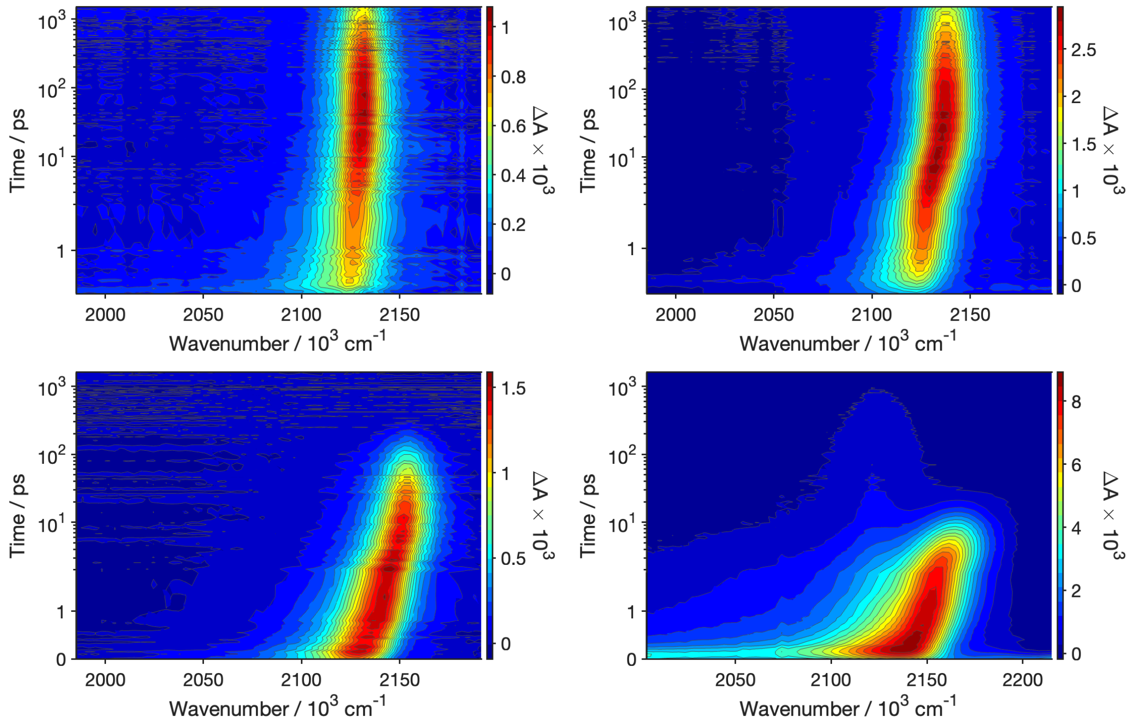

Figure S25 Transient IR absorption recorded with **PT-CAR** upon 400 nm excitation in CHX (top left), TOL (top right), THF (bottom left) and upon 530 nm excitation in DMSO (bottom right).

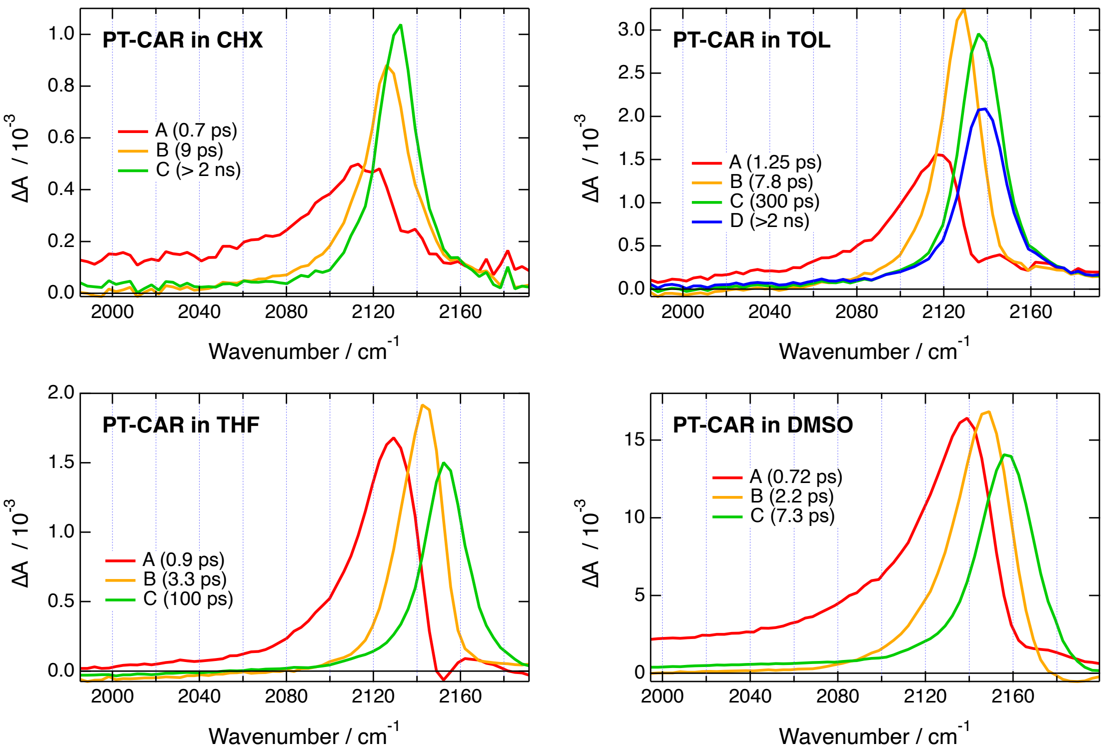

Figure S26 Evolution-associated difference absorption spectra and time constants obtained from a global analysis of the TRIR data shown in Figure S25 assuming a series of successive exponential steps.

PT-PT’

The TRIR spectra measured with **PT-PT’** in CHX consist in an intense and broad band at 2055 cm^{-1} and a smaller one at 2128 cm^{-1}(Figures S27 and S28). Given that **PT-PT’** can be considered as a linear D-A-D dye, only the antisymmetric -C≡C- stretching mode should be IR active if the excitation were evenly distributed over the whole molecule. Some asymmetry in the distribution of the excitation due to the different alkyl chains on the PT nitrogen atoms might explain the presence of the weak band, that most probably correspond to the symmetric -C≡C- stretching mode. In THF and DMSO, this spectrum transforms in less than 500 fs into one with a single band, shifting within 10 ps to about 2160 cm^{-1}, as observed with the previous two dyes. These results suggest that excitation remains mostly delocalised in CHX, but localises rapidly on one of the two PT-BTD ends in polar media.

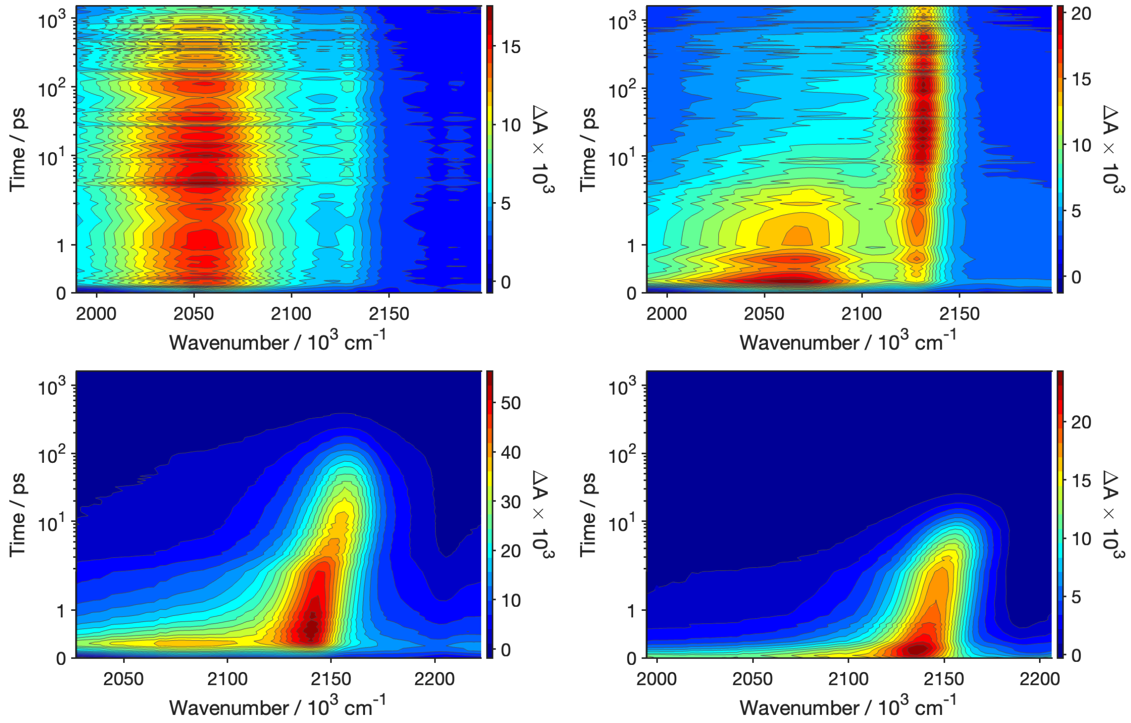

Figure S27 Transient IR absorption recorded upon 530 nm excitation of **PT-PT’** in CHX (top left), TOL (top right), THF (bottom left) and DMSO (bottom right).

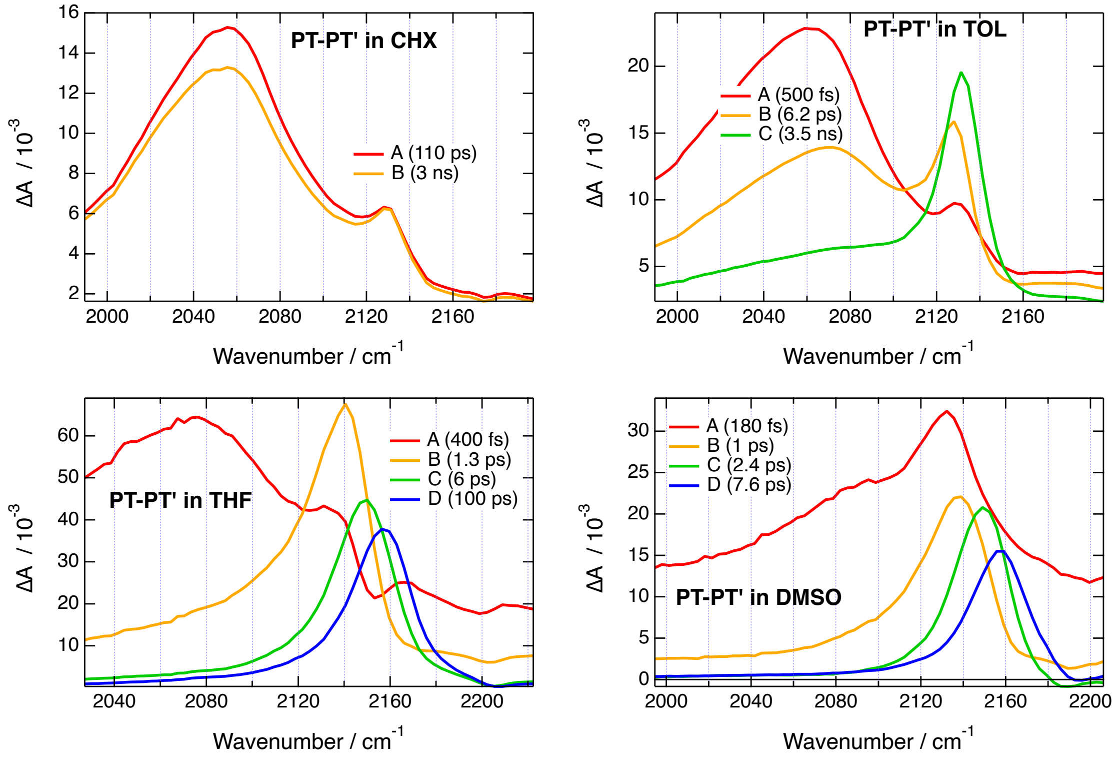

Figure S28 Evolution-associated difference absorption spectra and time constants obtained from a global analysis of the TRIR data shown in Figure S27 assuming a series of successive exponential steps.

PT-DMA

The transient spectra measured with **PT-DMA** in CHX are similar to those recorded with **PT-PT'** with an intense band at 2068 cm^{-1} and a much weaker one at 2128 cm^{-1} (Figures S29 and S30) and can be interpreted likewise. When going to THF, this spectrum evolves in less than 1 ps into one with a single band at 2122 cm^{-1}. Afterwards, a prominent shoulder at 2148 cm^{-1} develops in 2-3 ps and the spectrum remains unchanged before decaying with a approx. 240 ps time constant (Figure S30). In DMSO, this shoulder vanishes in 7 ps and only a single band at 2120 cm^{-1} is visible in the spectrum of the relaxed excited state. This band is not present with the previously discussed dyes with weaker D', and is assigned to the -C≡C- stretching mode of the S_1 state with the CT excitation localised at the BTD-DMA end.

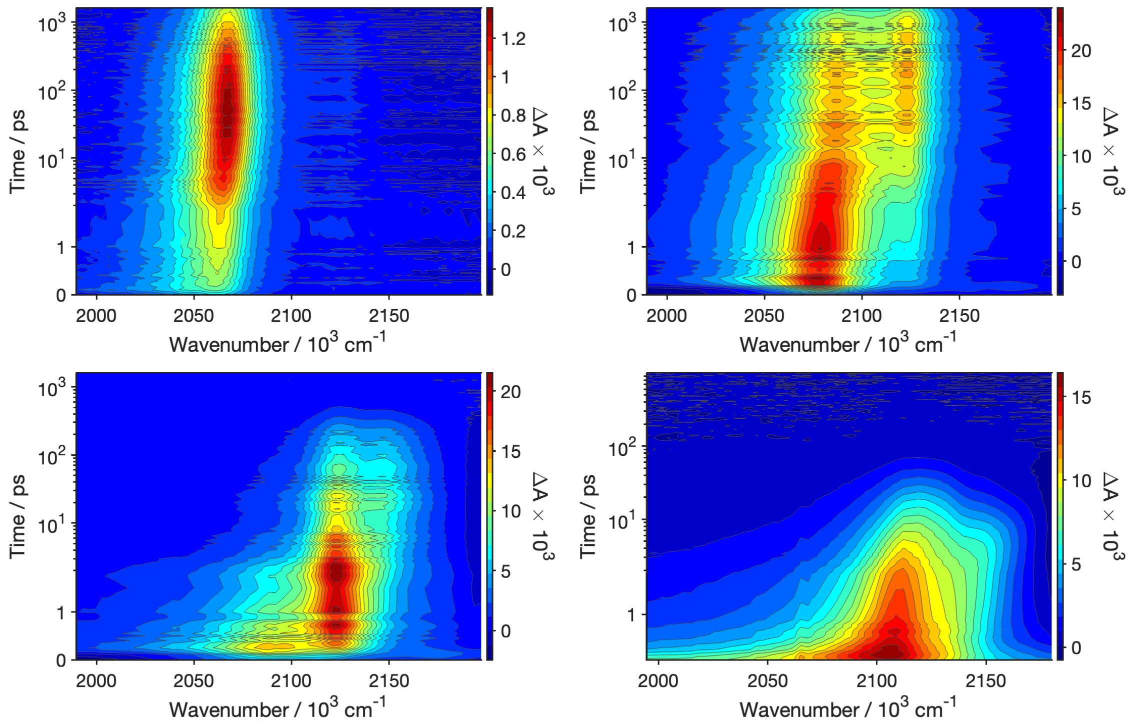

Figure S29 Transient IR absorption recorded with **PT-DMA** in CHX (400 nm excitation, top left) and in TOL (top right), THF (bottom left) and DMSO (bottom right) upon 530 nm excitation.

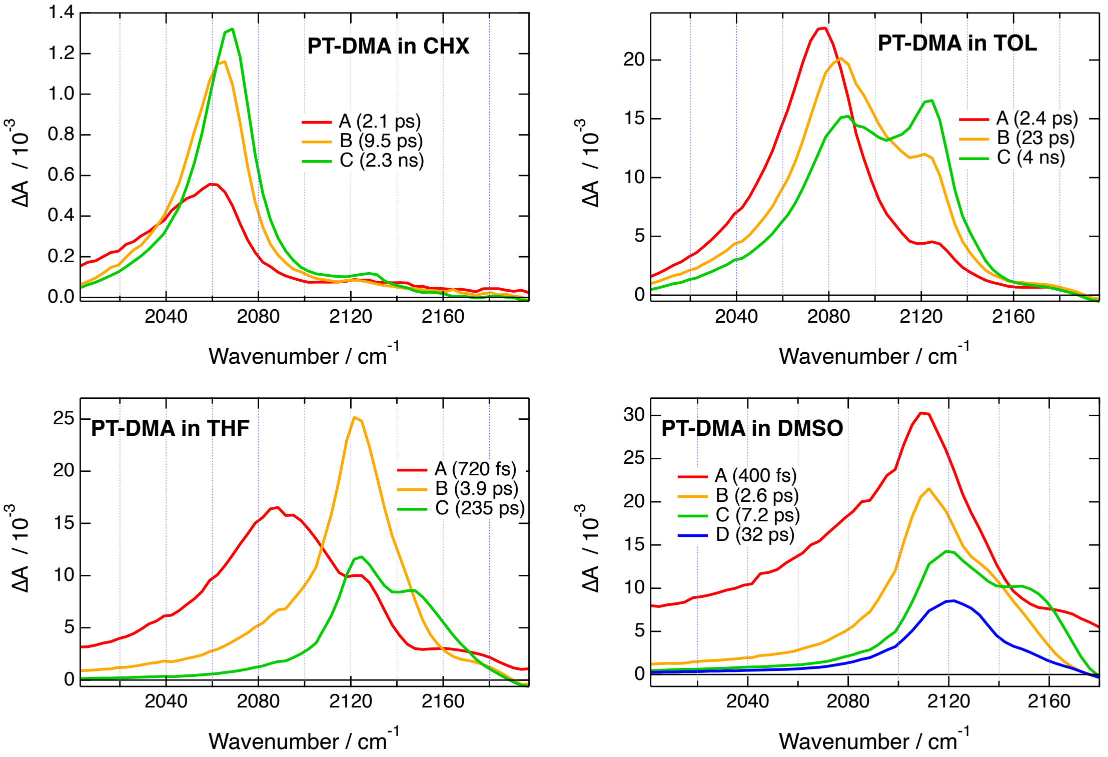

Figure S30 Evolution-associated difference absorption spectra and time constants obtained from a global analysis of the TRIR data shown in Figure S29 assuming a series of successive exponential steps.

PTO-DMA

This assignement of the 2120 cm^{-1} measured with **PT-DMA** in DMSO is supported by the results obtained with **PTO-DMA**, where PTO is a weaker donor than PT (Figures S31 and S32). In THF, the initial spectrum consists in a very broad band centred around 2090 cm^{-1}, which rapidly shifts and narrows to 2120 cm^{-1}. In DMSO, this dynamics is even faster and the band is finally at 2125 cm, similar to **PT-DMA**.

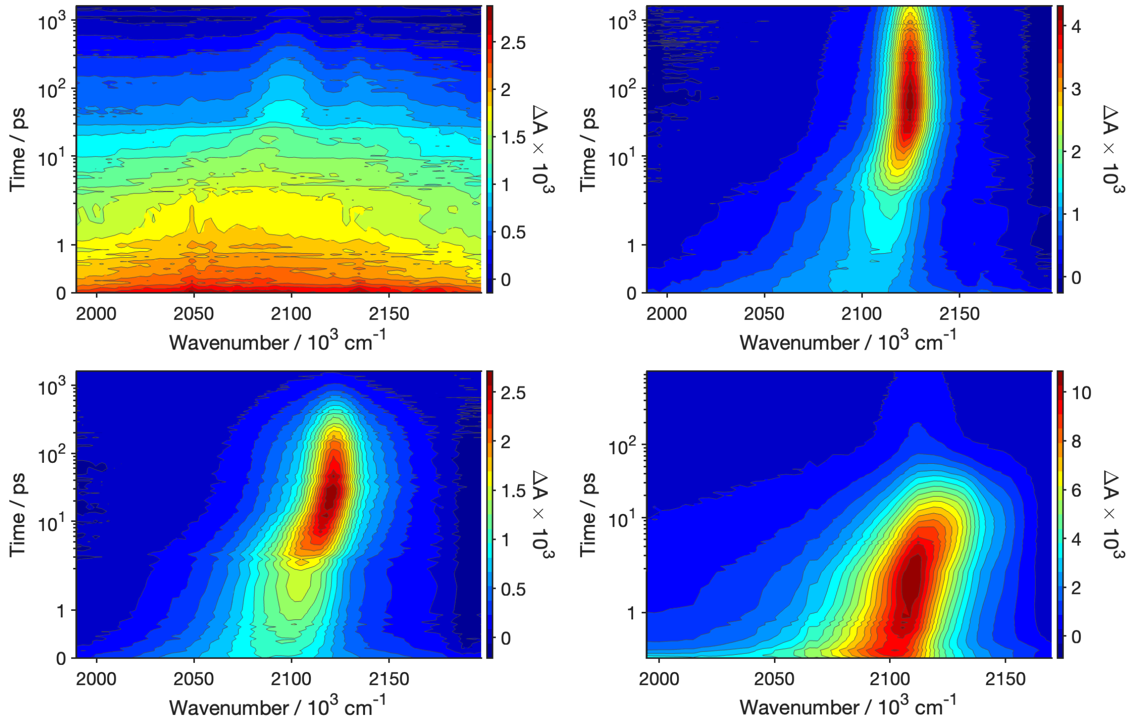

Figure S31 Transient IR absorption recorded upon 400 nm excitation of **PTO-DMA** in CHX (top left), TOL (top right), THF (bottom left) and DMSO (bottom right).

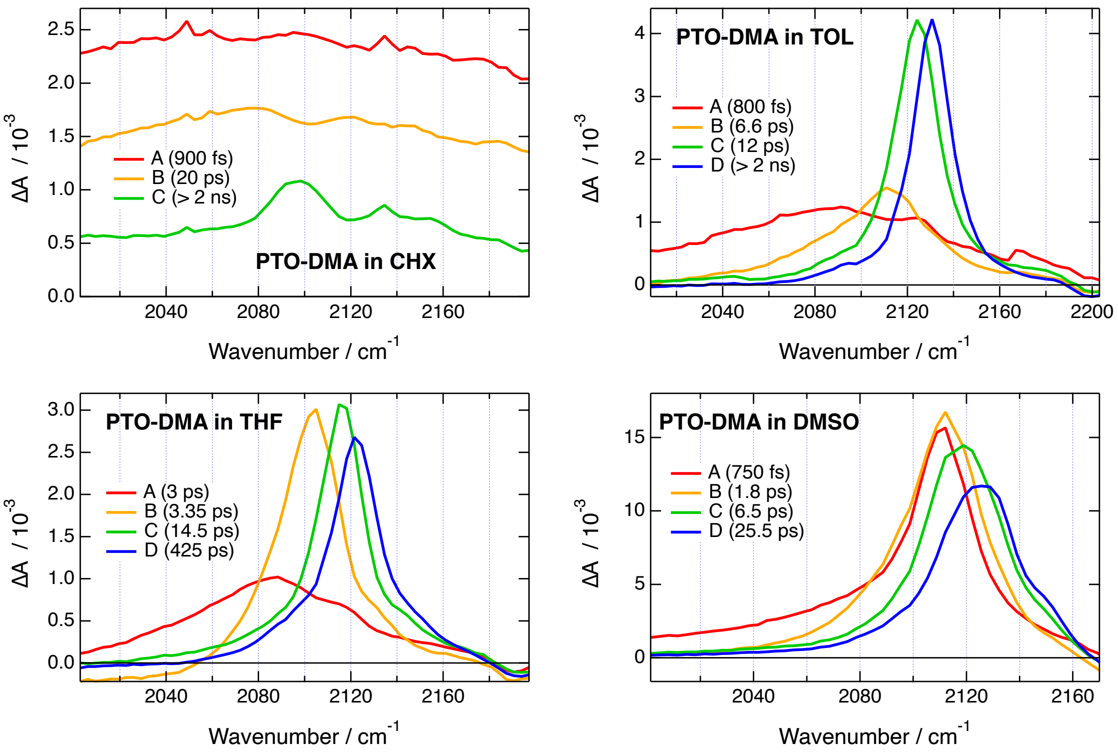

Figure S32 Evolution-associated difference absorption spectra and time constants obtained from a global analysis of the TRIR data shown in Figure S31 assuming a series of successive exponential steps.

CARs-PT-CARs

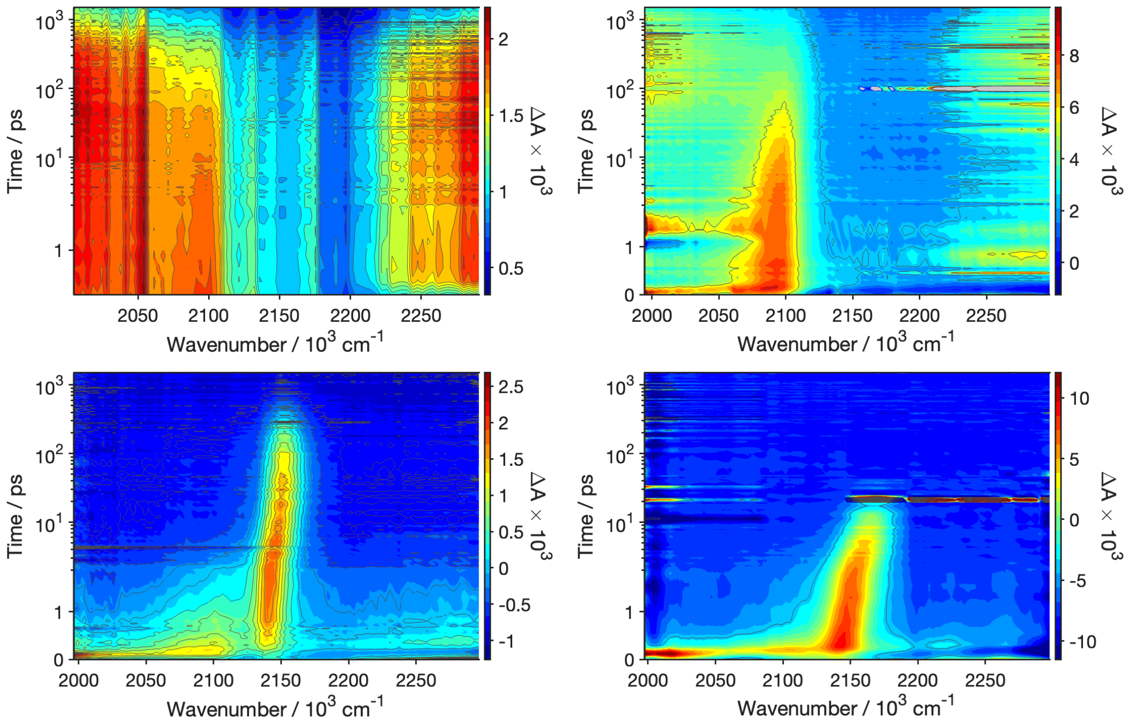

Figure S33 Transient IR absorption recorded upon 530 nm excitation of **CARs-PT-CARs** in CHX (top left) and upon 400 nm excitation in CHX(top right), THF (bottom left) and DMSO (bottom right).

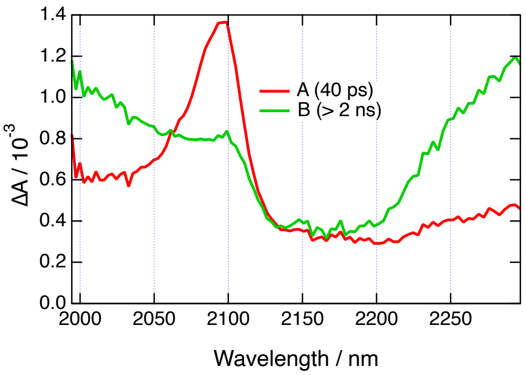

Figure S34 Evolution-associated difference absorption spectra and time constants obtained from a global analysis of the TRIR data measured with **CARs-PT-CARS** in CHX upon 400 nm excitation assuming a series of successive exponential steps.

CAR-PT-CAR

The transient spectra recorded with **CAR-PT-CAR** in TOL also exhibit the broad background additionally to a band around 2150 cm^{-1}(Figures S35 and S36). Little dynamics are observed apart from a partial increase of the -C≡C- band intensity. These results point to delocalised excitation in this solvent. In medium polar media, the early spectra are similar to those in TOL, but the electronic background signal decays in a few ps while the vibrational band at 2150 cm^{-1} decays on the hundreds of ps timescale. The disappearance of the broad signal can be interpreted as a localisation of the excitation, most probably on a PT-BTD pair according to the 2150 cm^{-1} frequency. Similar dynamics are observed in DMSO, but the vibrational band is at 2120 cm^{-1}, i.e. 30 cm^{-1} down-shifted relatively to the less polar solvents, and 40 cm^{-1} down-shifted relative to the band measured with the **PT-CAR** analogue in the same solvent. These results point to the occurrence of ESSB as well, but not to a localisation of the excitation on a PT-BTD D-A pair like in **CARs-PT-CARs** and **PT-CAR**. None of the dyes with D'=CAR discussed so far exhibit such 2120 cm^{-1} band. Therefore, we attribute it to the -C≡C- stretching mode of the CT state localised on one of the two BTD-CAR ends of the molecule. It should also be noted that the lifetime of the relaxed S_1 state of this 2B dye in DMSO is much larger, i.e. 710 ps, relatively to the 7.3 ps measured with the 1B analogue.

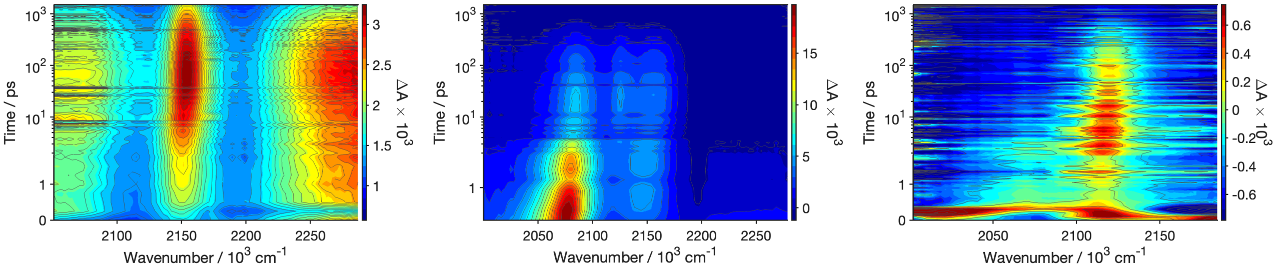

Figure S35 Transient IR absorption recorded with **CAR-PT-CAR** upon 400 nm excitation in TOL (left), and DMSO (right), and upon 530 nm excitation in THF (middle), .

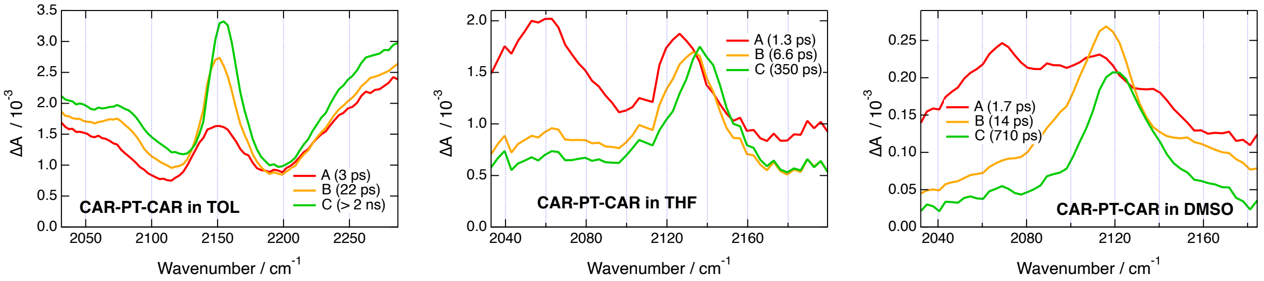

Figure S36 Evolution-associated difference absorption spectra and time constants obtained from a global analysis of the TRIR data shown in Figure S35 assuming a series of successive exponential steps.

PT'-PT-PT'

The TRIR spectra measured with **PT'-PT-PT'** in CHX and TOL are similar to those obtained with **PT-PT'** with the additional presence of the electronic background signal (Figures S37 and S38), suggesting a delocalised excitation over the two branches. Localisation occurs in THF and DMSO, as testified by the complete decay of the background signal, and only a band around 2135 cm^{-1} is visible after a few ps. This band is ~20 cm down-shifted compared to that measured with **PT-PT'** and its lifetime is 10 times as long. This frequency shift could arise from a slight disparity in donating strength of the central and terminal phenothiazine units due to their different substituents on the N atom.

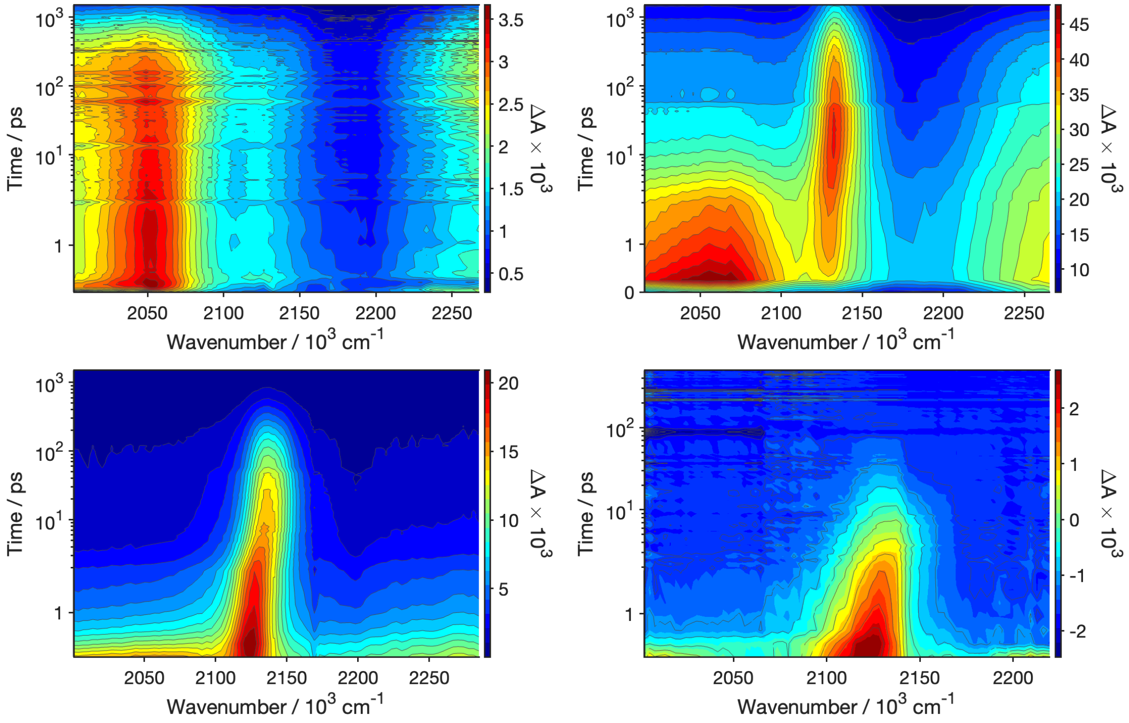

Figure S37 Transient IR absorption recorded with **PT'-PT-PT'** upon 530 nm excitation in TOL (top right), THF (bottom left) and 400 nm excitation in CHX (top left) and DMSO (bottom right).

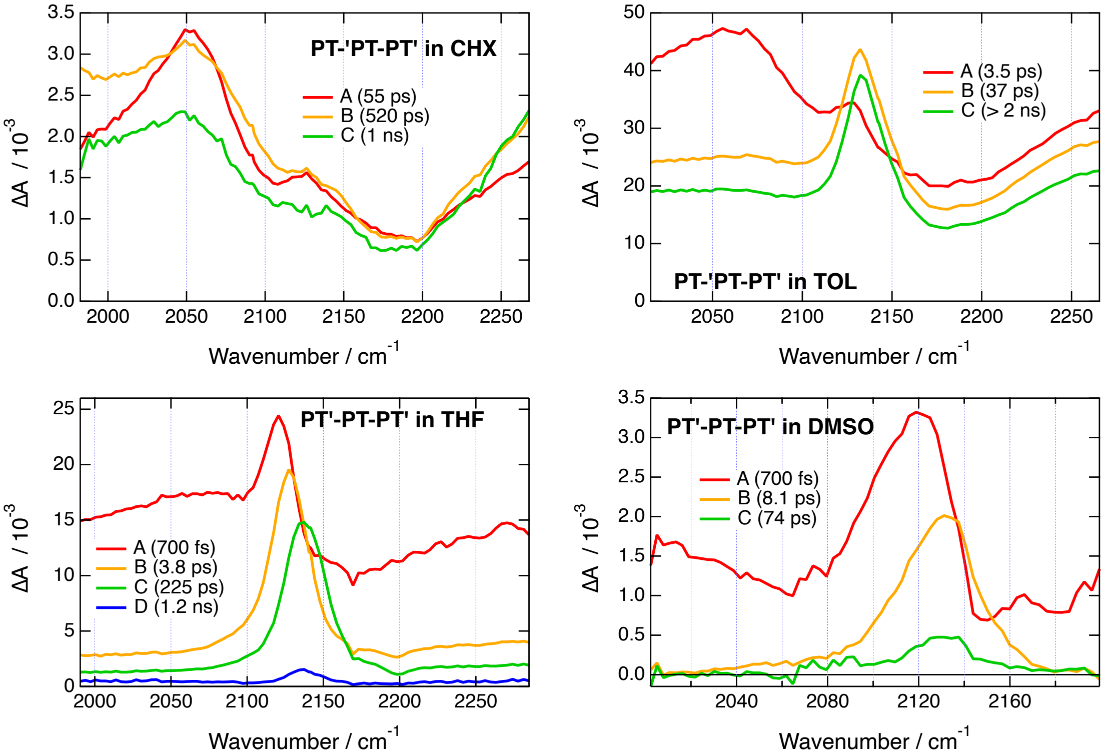

Figure S38 Evolution-associated difference absorption spectra and time constants obtained from a global analysis of the TRIR data shown in Figure S37 assuming a series of successive exponential steps.

DMA-PT-DMA

In CHX, the transient spectra of **DMA-PT-DMA** are also similar to those of the 1B analogue, except for the presence of the background signal (Figures S39 and S40). In polar solvents, this broad feature vanishes rapidly and a two partially overlapping vibrational bands appears within 1 ps at 2120 and 2150 cm^{-1}. The low-frequency band decreases partially in 18 ps, while the other one decays totally. Finally, the weak residual band around 2120 cm^{-1} decays completely on a few hundreds of ps timescale. This band is also present with **PT-DMA** (Figure S30) and was attributed to the -C≡C- vibration of the CT state localised at the BTD-DMA end. This band decays in about 200 ps vs. 32 ps for **PT-DMA**. The same assignment can be done here. On the other hand, the 2150 cm^{-1} band observed transiently is probably due the PT-BTD -C≡C- vibration. This indicates that excitation first localises on a single branch with a possible temporary equilibrium between the PT-BTD and BTD-DMA CT states, and finally localises on a BTD-DMA end as solvent relaxation takes place.

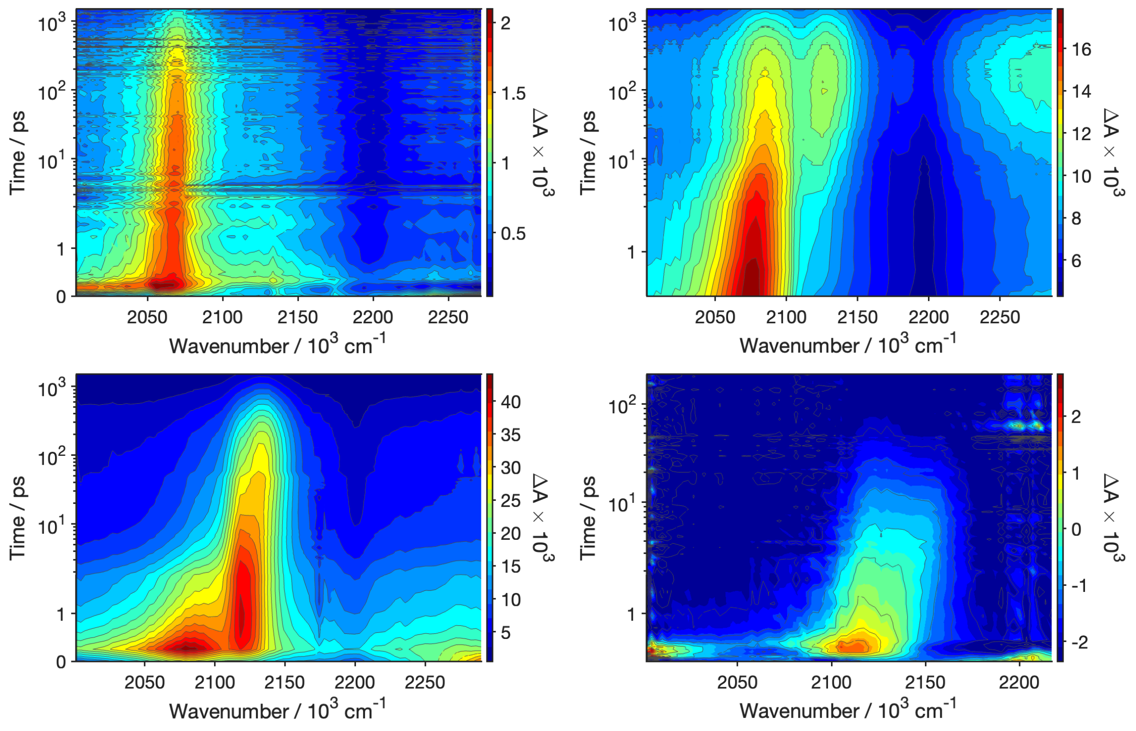

Figure S39 Transient IR absorption recorded with **DMA-PT-DMA** upon 530 nm excitation in TOL (top right), THF (bottom left) and 400 nm excitation in CHX (top left) and DMSO (bottom right).

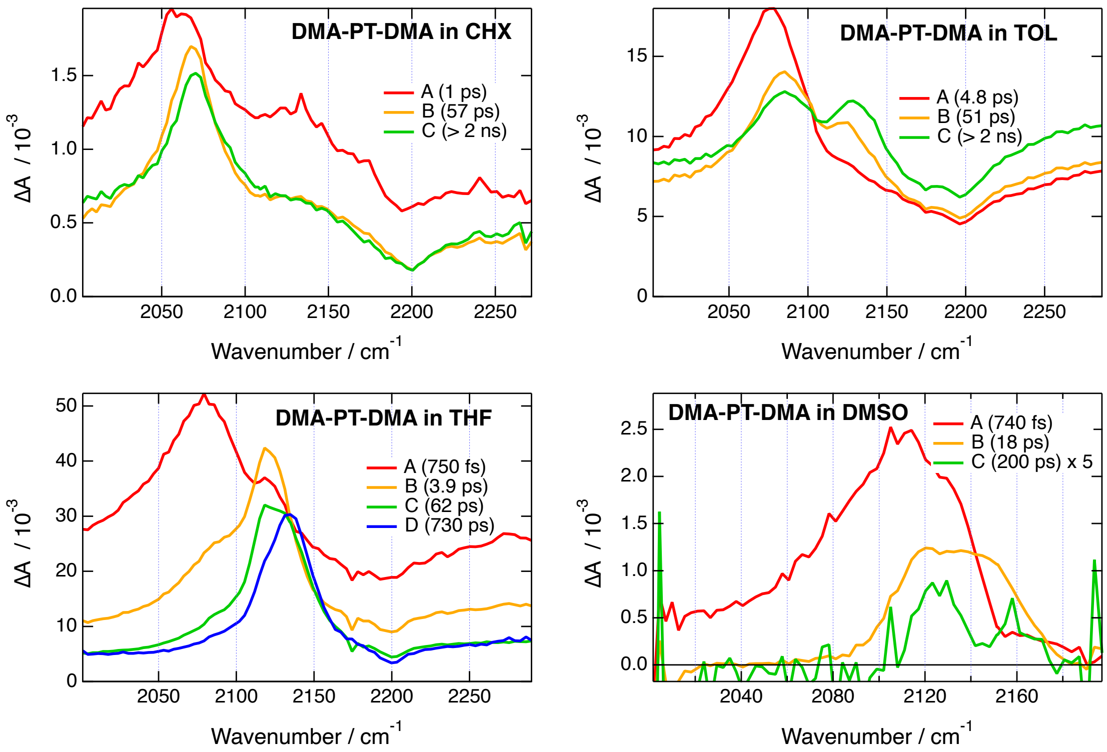

Figure S40 Evolution-associated difference absorption spectra and time constants obtained from a global analysis of the TRIR data shown in Figure S39 assuming a series of successive exponential steps.

DMA-PTO-DMA

Contrary to the other 2B dyes in CHX/TOL, the TRIR spectra recorded with **DMA-PTO-DMA** in TOL do not exhibit any electronic background signal, but are similar to those measured with the **PTO-DMA** with a single band around 2125 cm^{-1} (Figures S41 and S42). This points to a localisation at a BTD-DMA end of the molecule. The same behaviour is observed in THF and in DMSO, i.e., the spectra resemble those found with **PTO-DMA** (Figure S32). However, as observed with the other dyes, the decay of the relaxed S_1 state in DMSO is significantly slower than for the single branch.

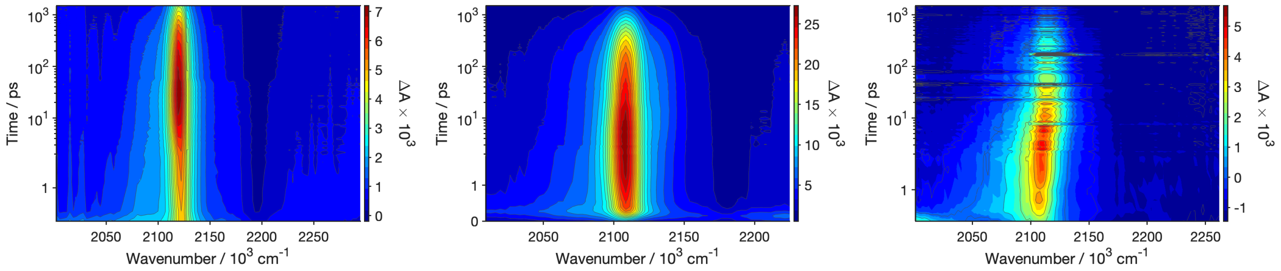

Figure S41 Transient IR absorption recorded with **DMA-PTO-DMA** upon 530 nm excitation in TOL (left) and THF (middle) and 400 nm excitation in DMSO (right).

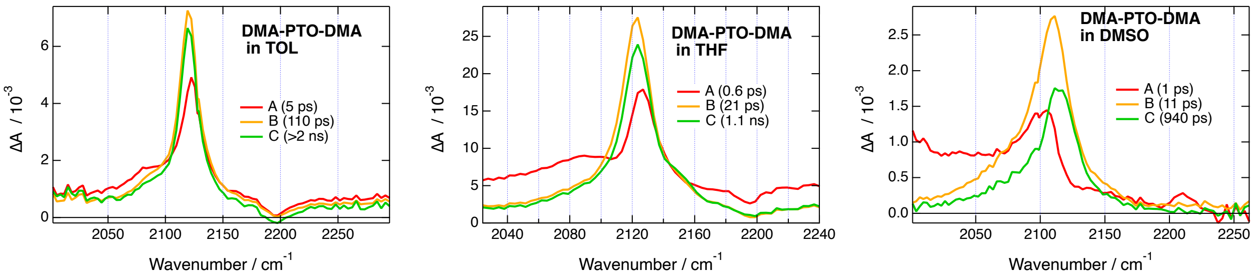

Figure S42 Evolution-associated difference absorption spectra and time constants obtained from a global analysis of the TRIR data shown in Figure S41 assuming a series of successive exponential steps.

S2.6 Molecular Dynamics (MD) simulations

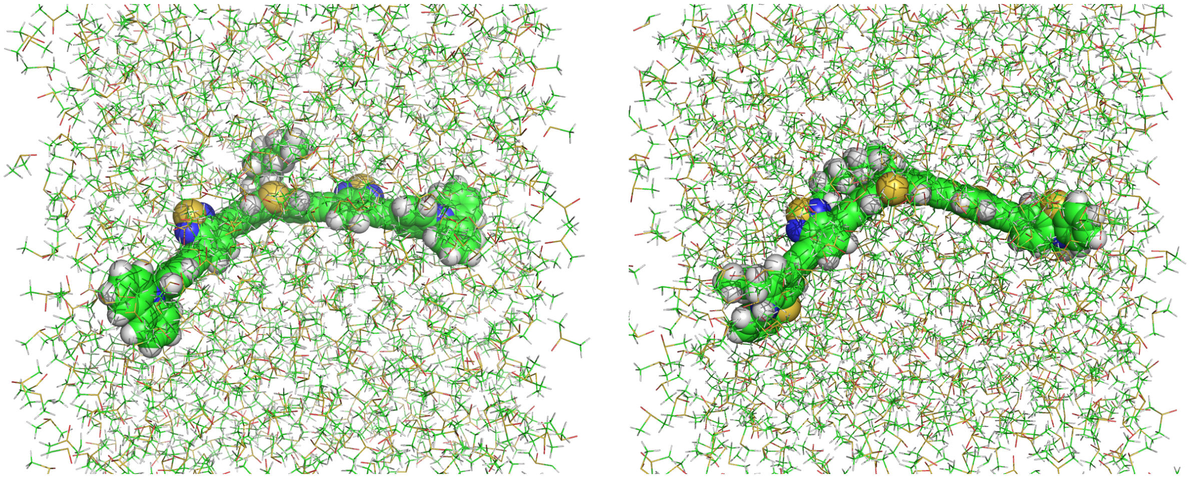

Figure S43 Snapshot of a MD simulation of **CAR-PT-CAR** (left) and **PT'-PT-PT'** (right) in DMSO.

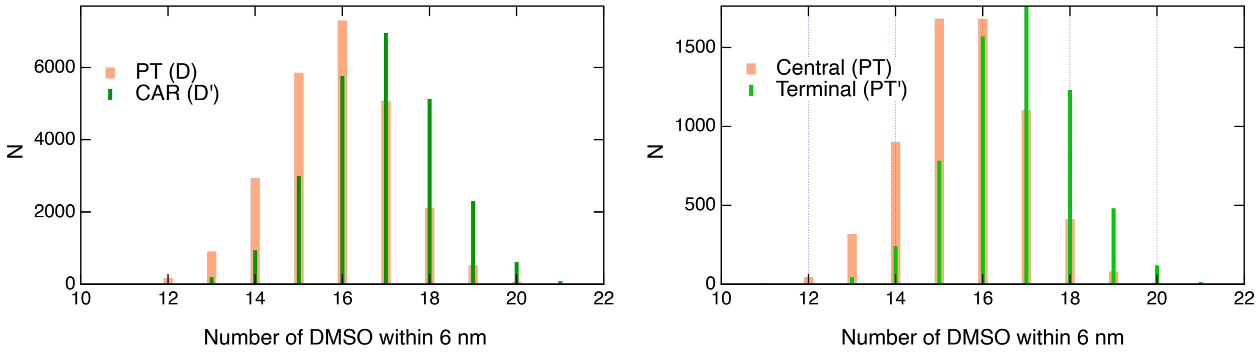

Figure S44 Histogram of the number of DMSO molecules within a centre-of-mass (COM) distance of 6 nm of the central PT and one of the terminal carbazole (left) or PT' (right) units of **CAR-PT-CAR** and **PT'-PT-PT'**, respectively. This illustrates the larger exposure to solvent of the end donors, D', compared to the central donor, D.

S2.7 NMR spectroscopy and MS spectrometry

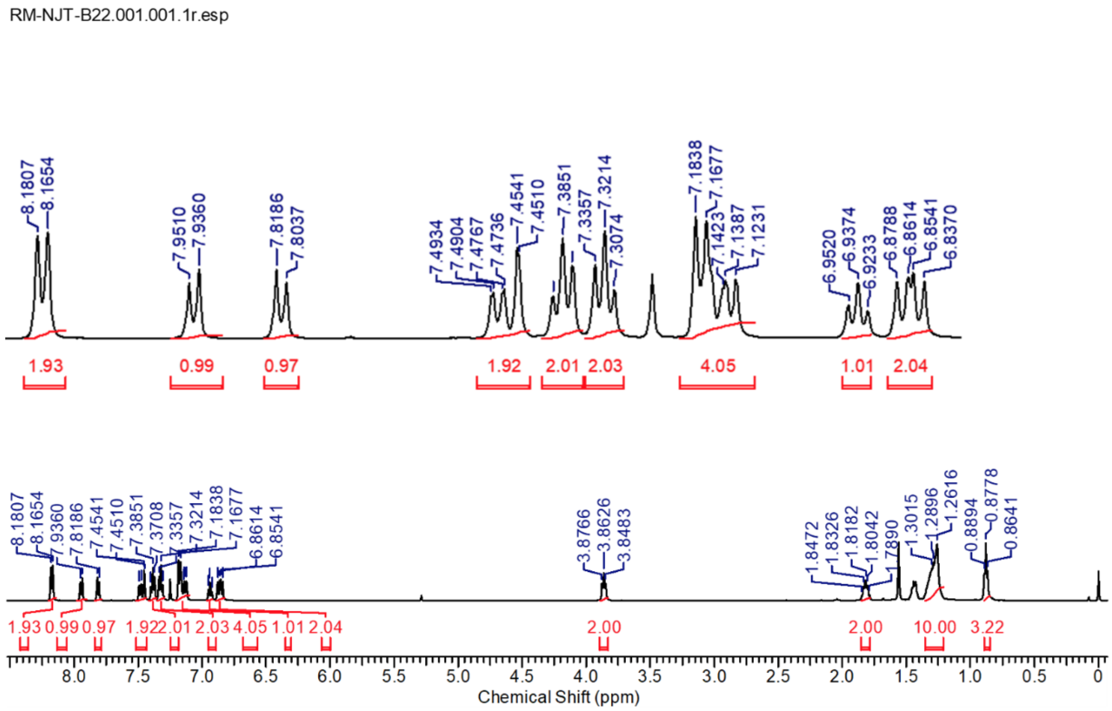

Figure S45 ^{1}H NMR spectrum of **PT-CARs**.

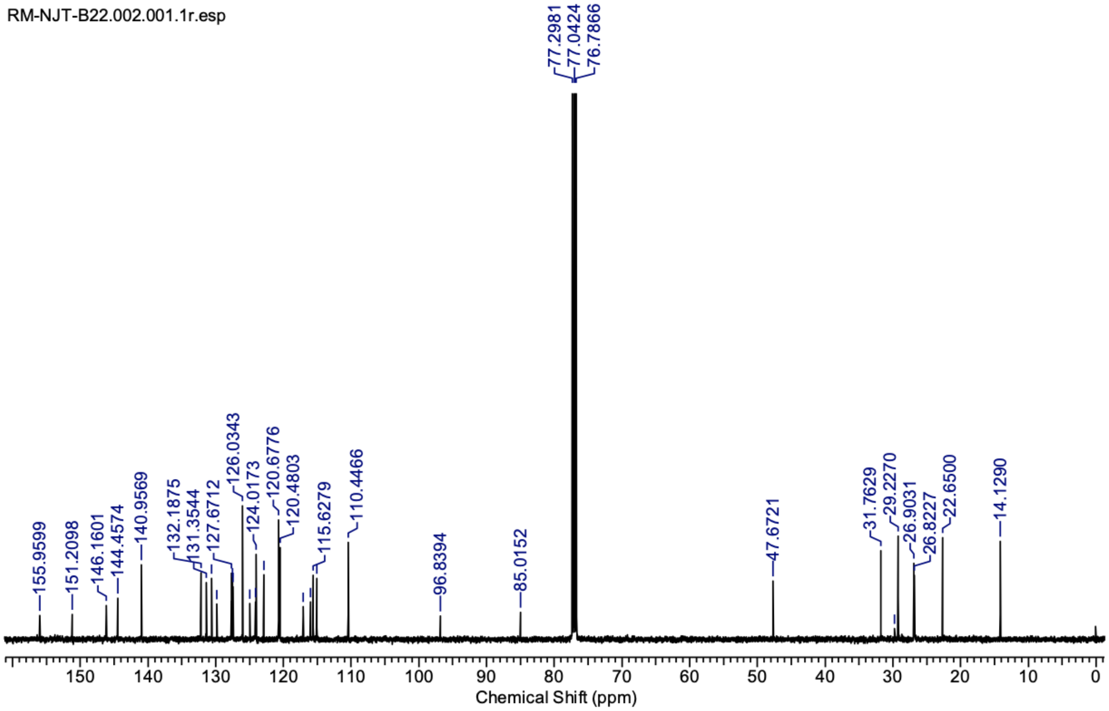

Figure S46 ^{13}C NMR spectrum of **PT-CARs**.

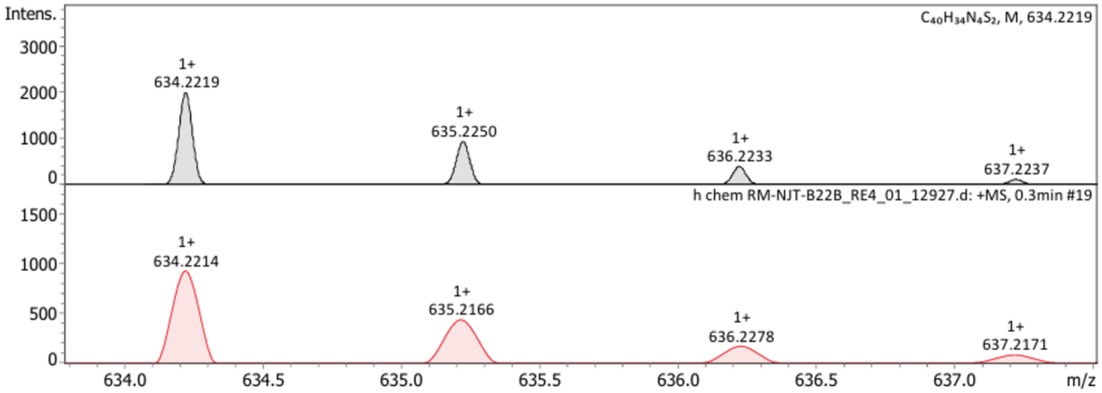

Figure S3. HRMS of **PT-CARs**.

Figure S47 High resolution MS spectrogram of **PT-CARs**.

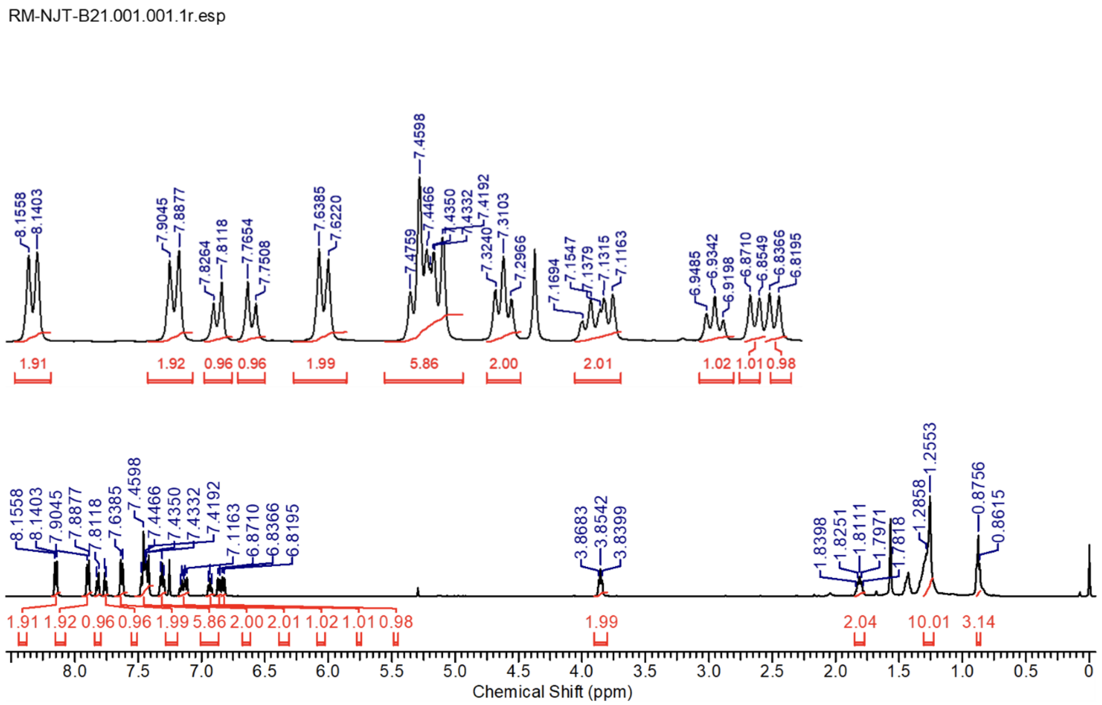

Figure S48 ^{1}H NMR spectrum of **PT-CAR**.

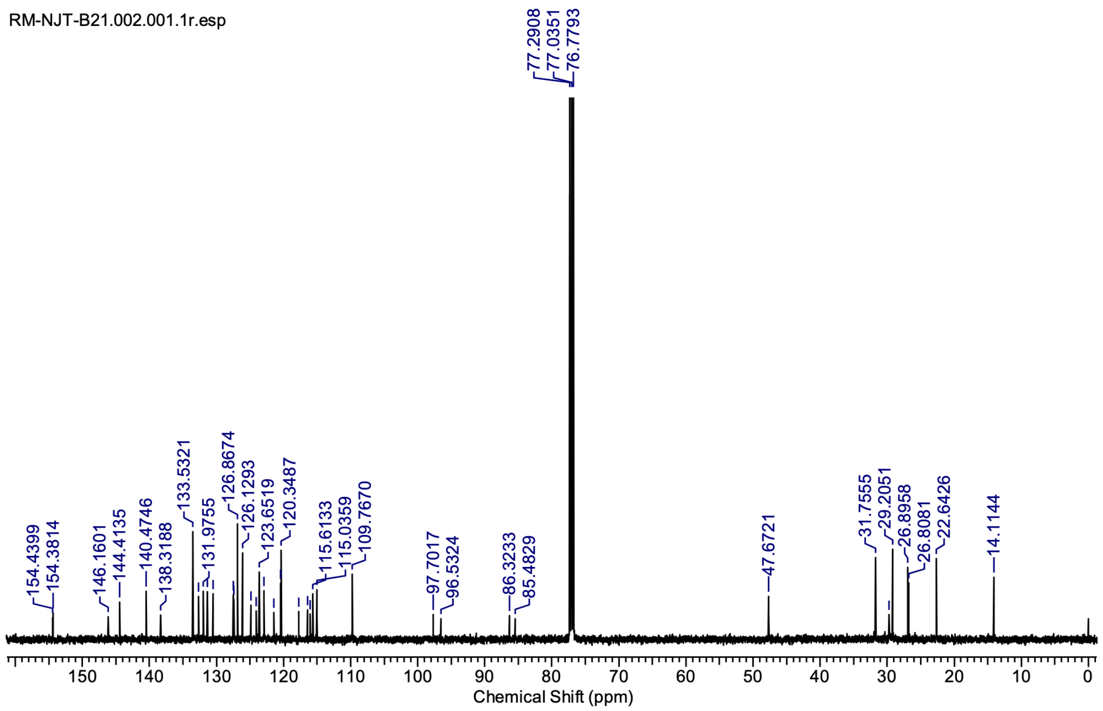

Figure S49 ^{13}C NMR spectrum of **PT-CAR**.

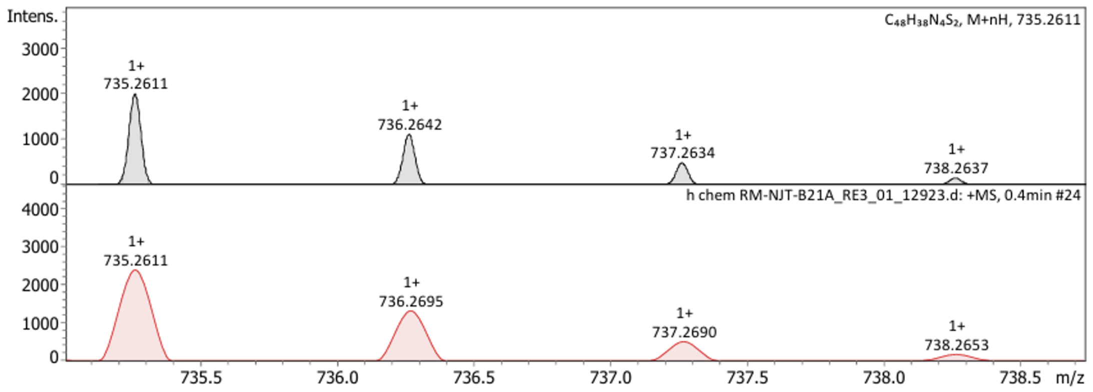

Figure S50 High resolution MS spectrogram of **PT-CAR**.

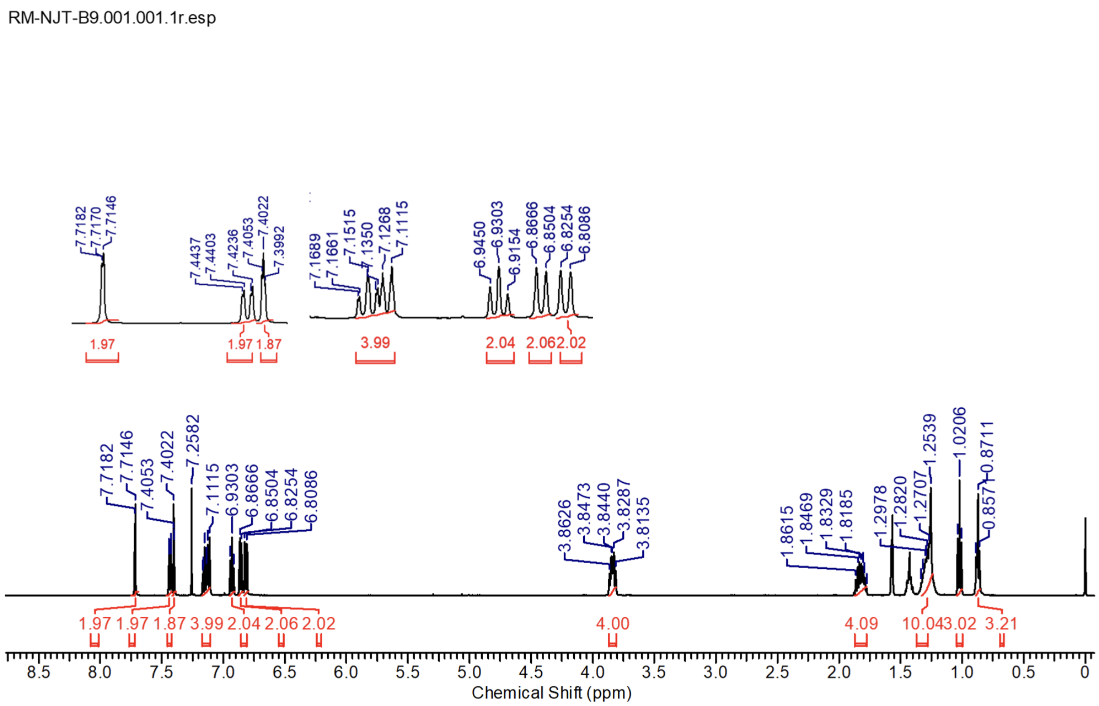

Figure S51 ^{1}H NMR spectrum of **PT-PT'**.

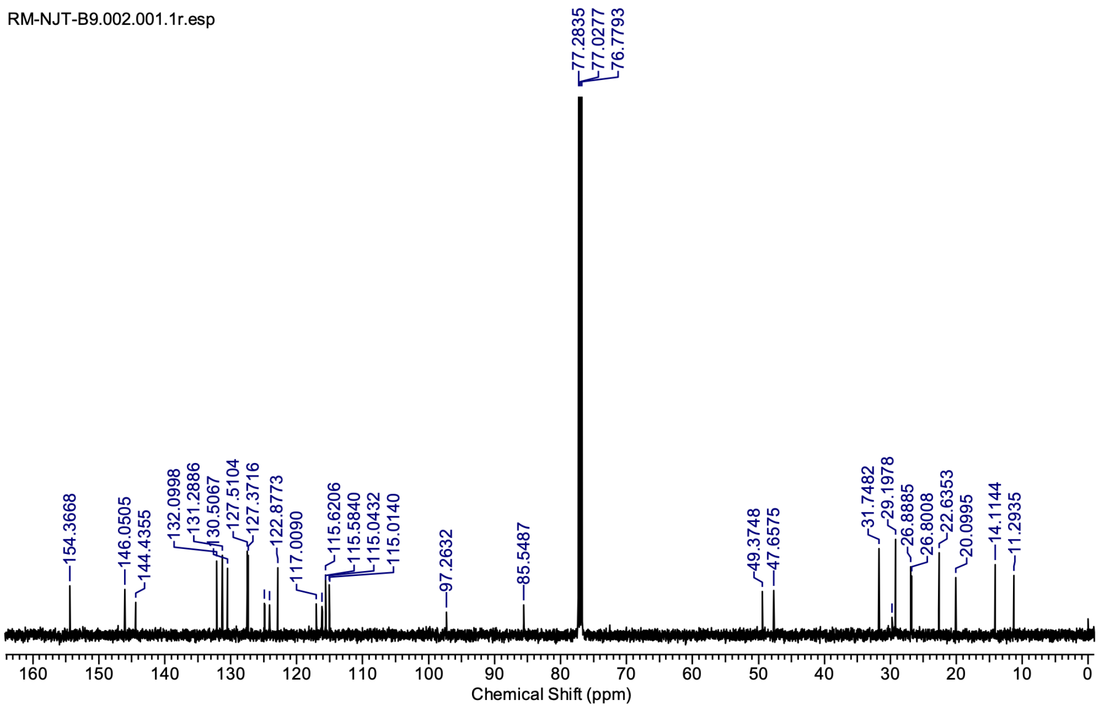

Figure S52 ^{13}C NMR spectrum of **PT-PT'**.

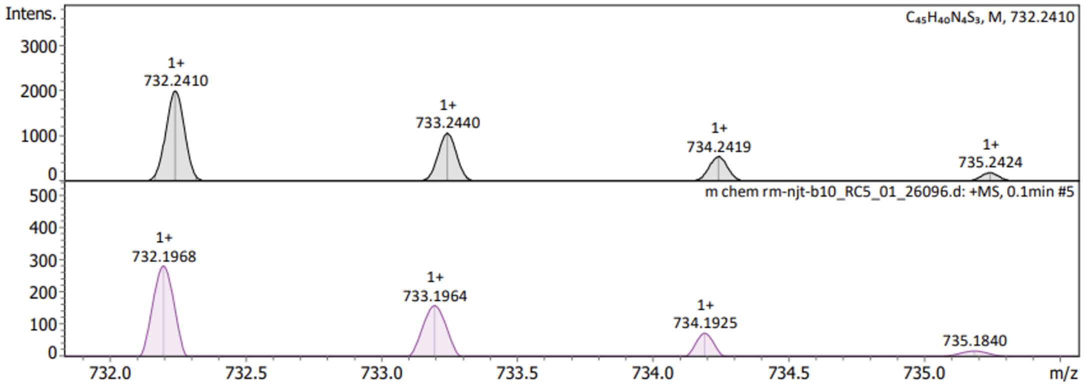

Figure S53 High resolution MS spectrogram of **PT-PT'**.

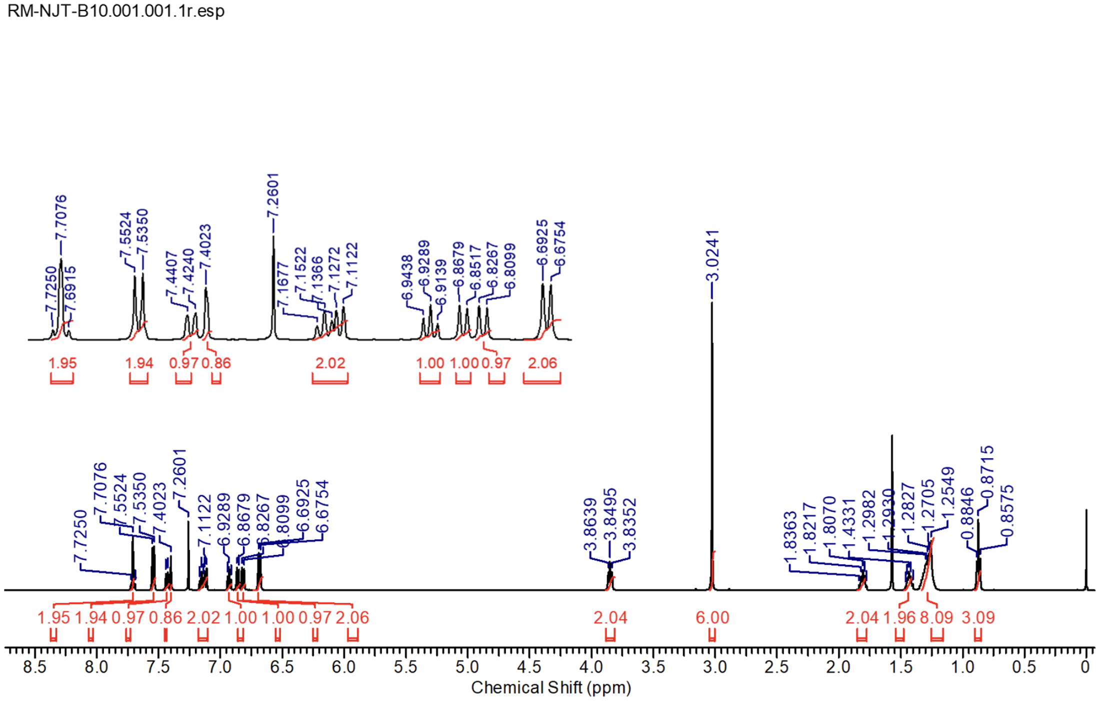

Figure S54 ^{1}H NMR spectrum of **PT-DMA**.

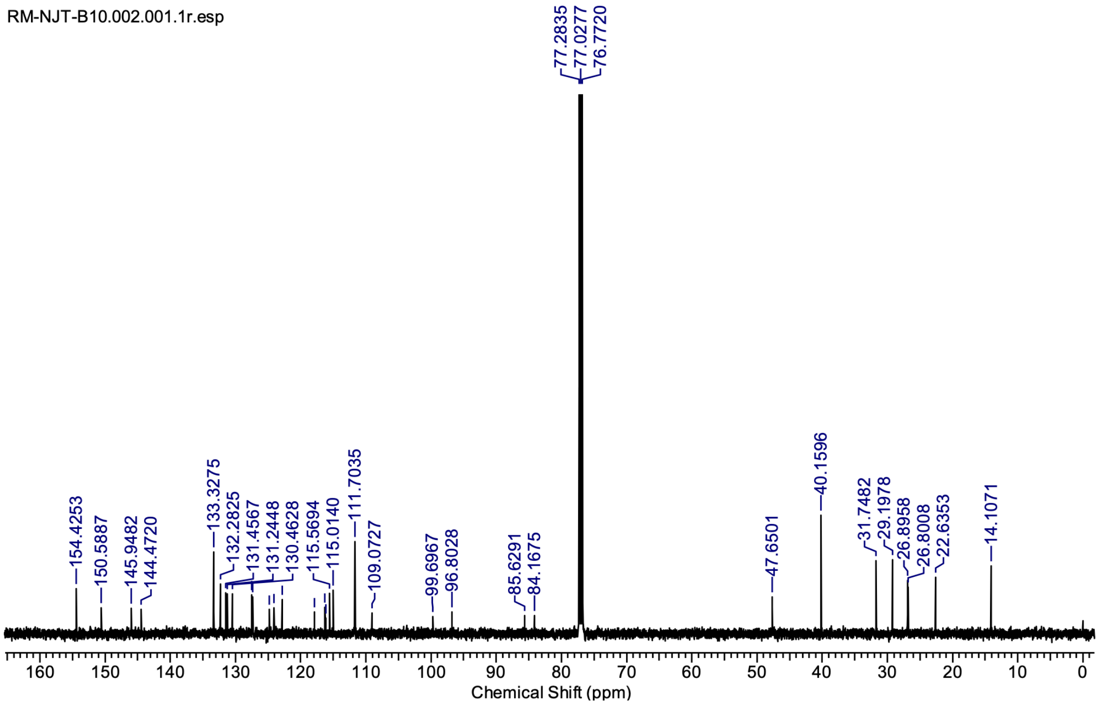

Figure S55 ^{13}C NMR spectrum of **PT-DMA**.

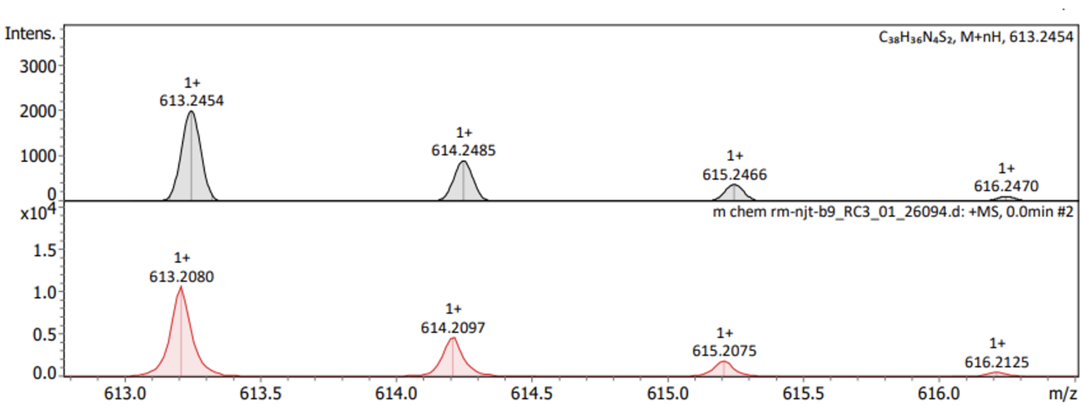

Figure S56 High resolution MS spectrogram of **PT-DMA**.

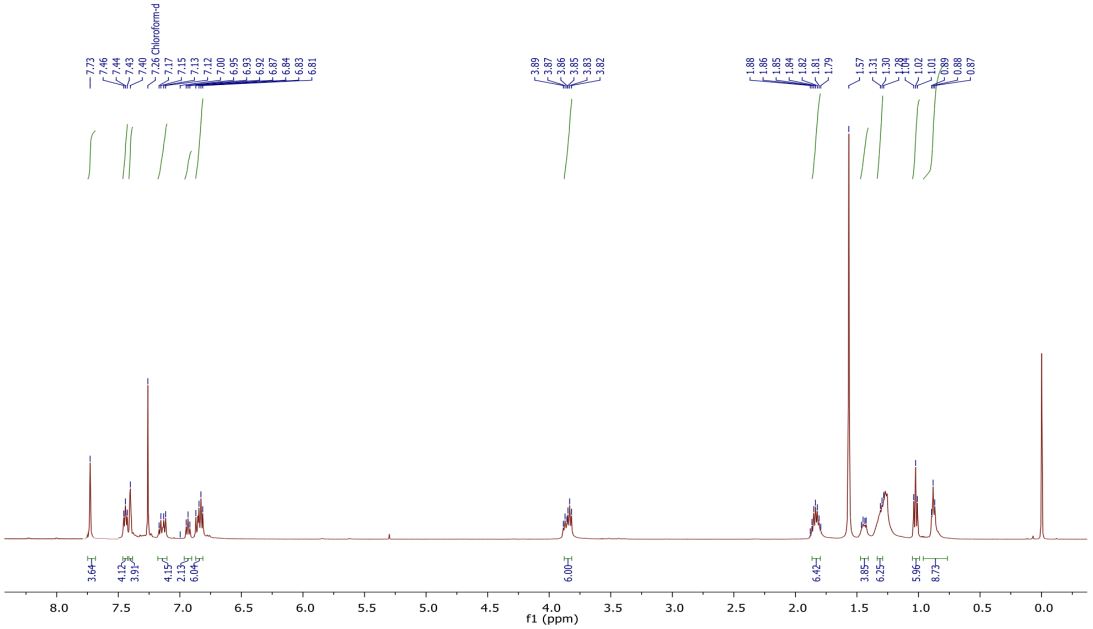

Figure S57 ^{1}H NMR spectrum of **PT’-PT-PT’**.

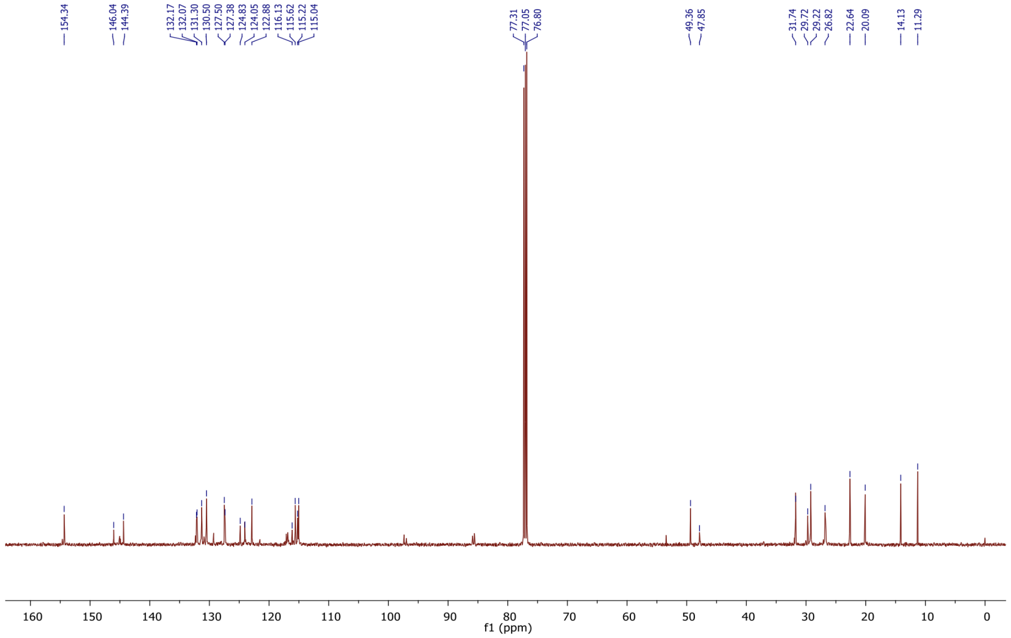

Figure S58 ^{13}C NMR spectrum of **PT’-PT-PT’**.

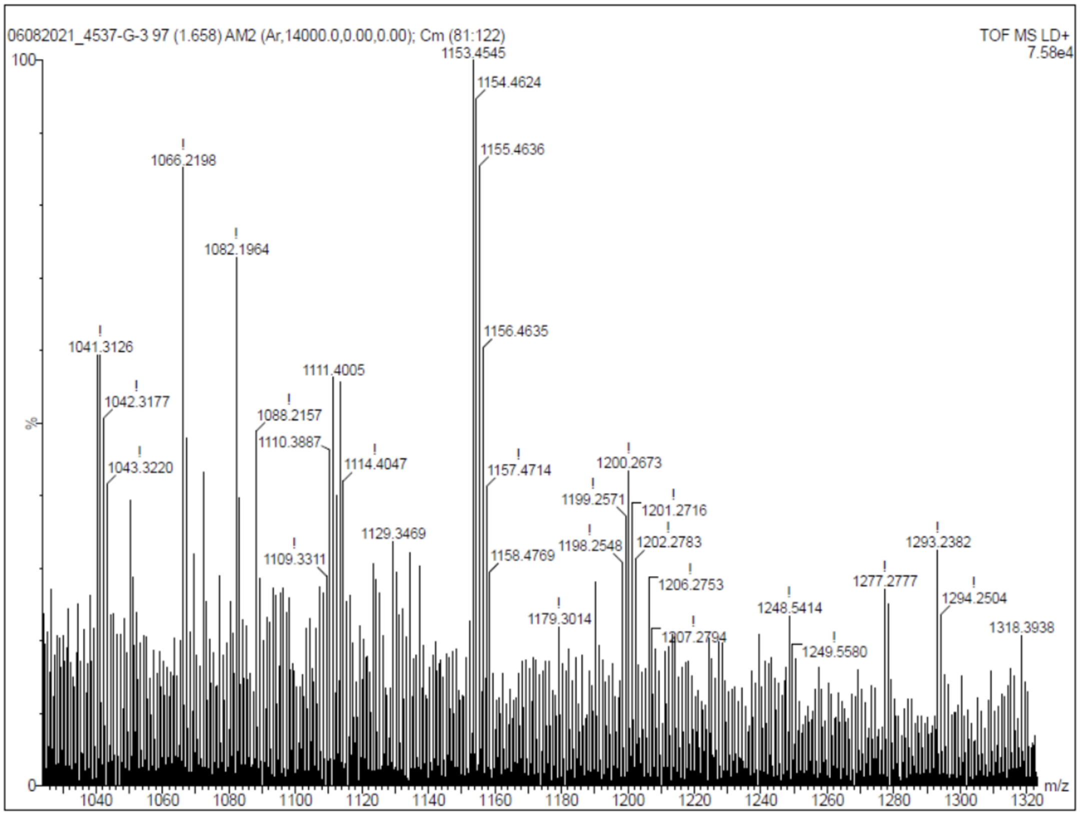

Figure S59 MALDI-TOF of **PT'-PT-PT'**.

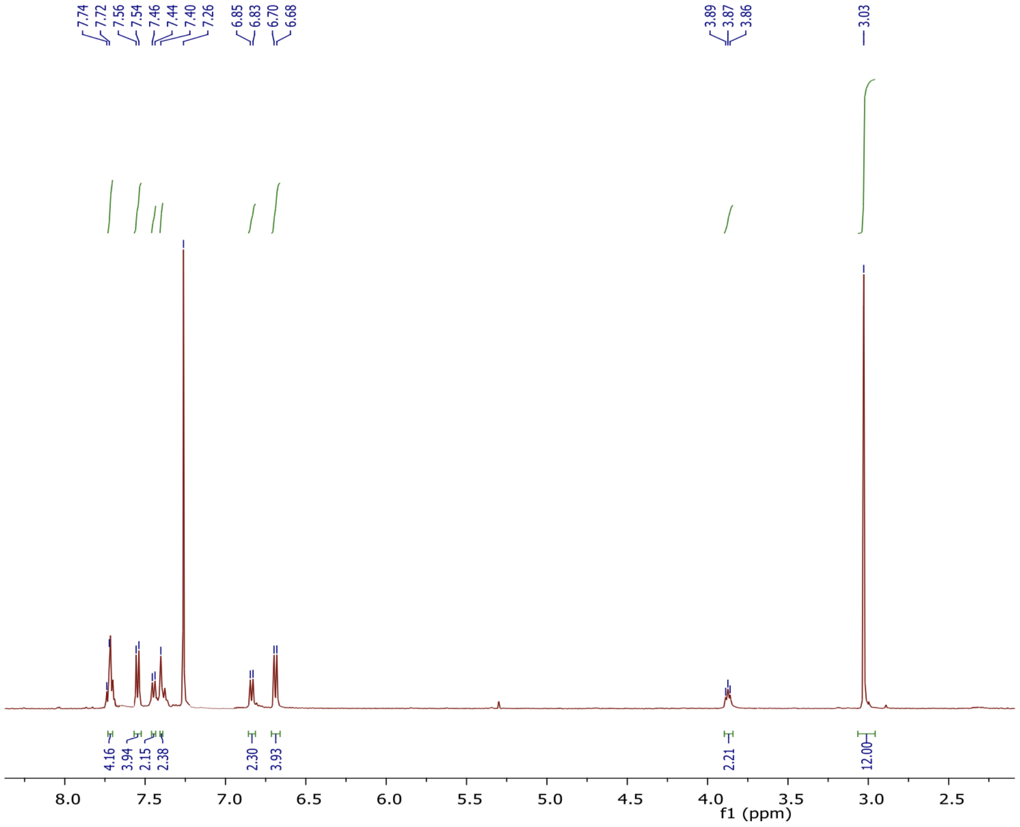

Figure S60 ^{1}H NMR spectrum of **DMA-PT-DMA**.

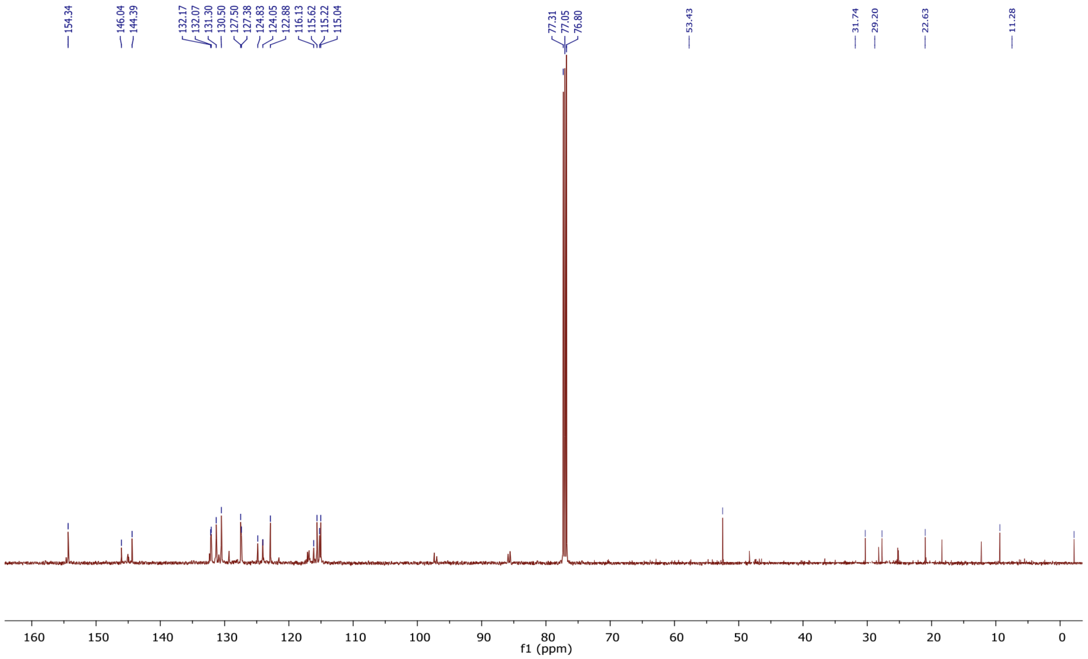

Figure S61 ^{13}C NMR spectrum of **DMA-PT-DMA**.

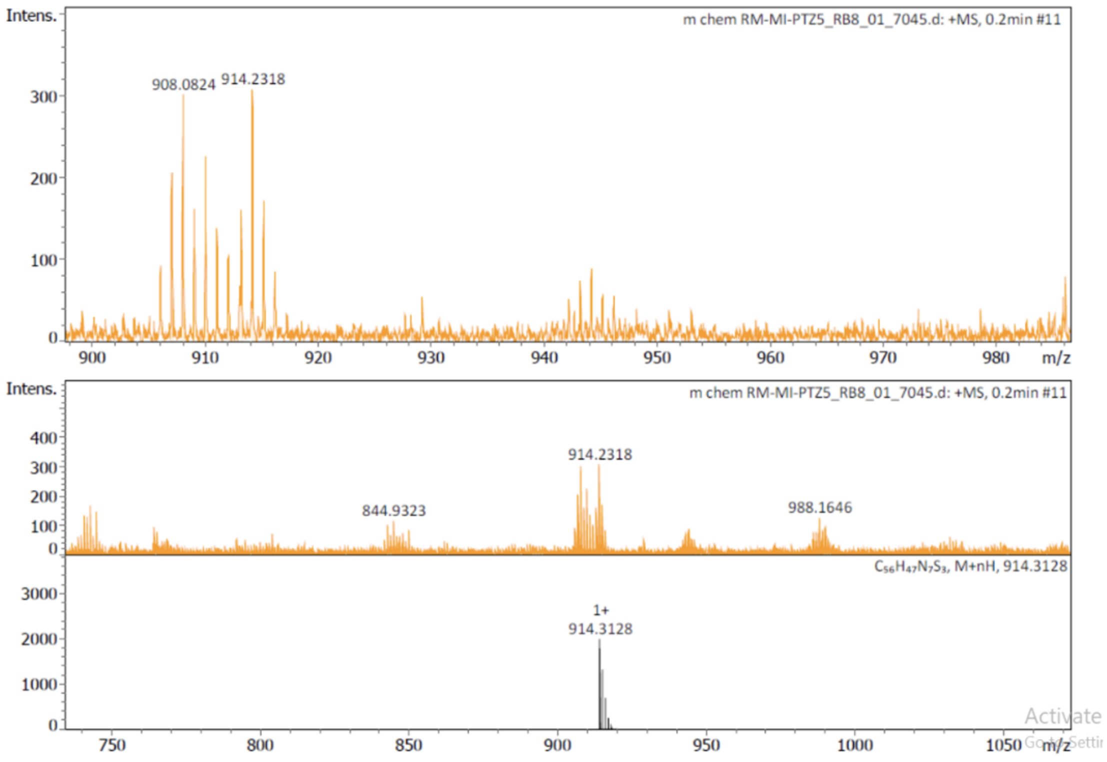

Figure S62 High resolution MS spectrogram of **DMA-PT-DMA**.

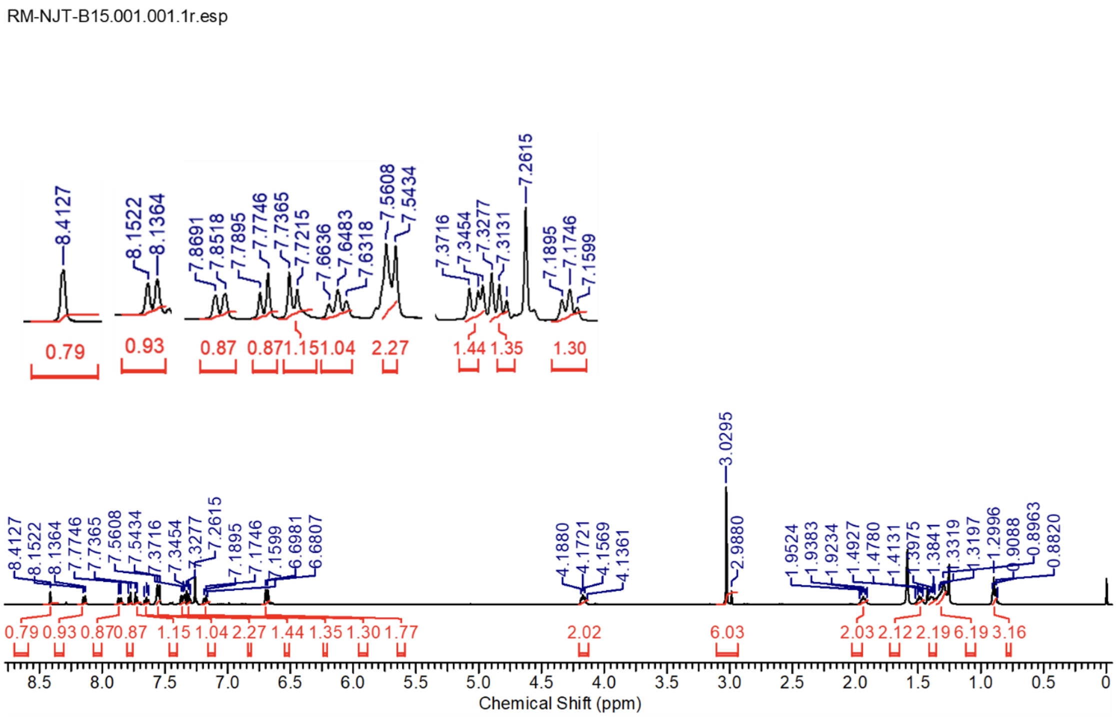

Figure S63 ^{1}H NMR spectrum of **PTO-DMA**.

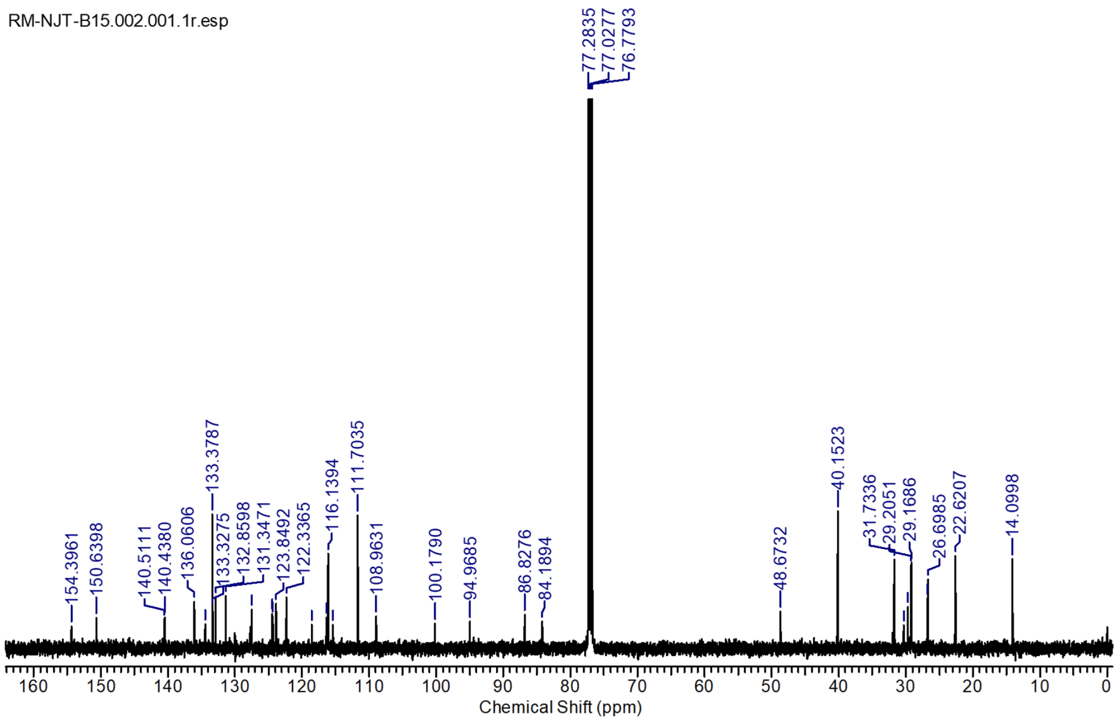

Figure S64 ^{13}C NMR spectrum of **PTO-DMA**.

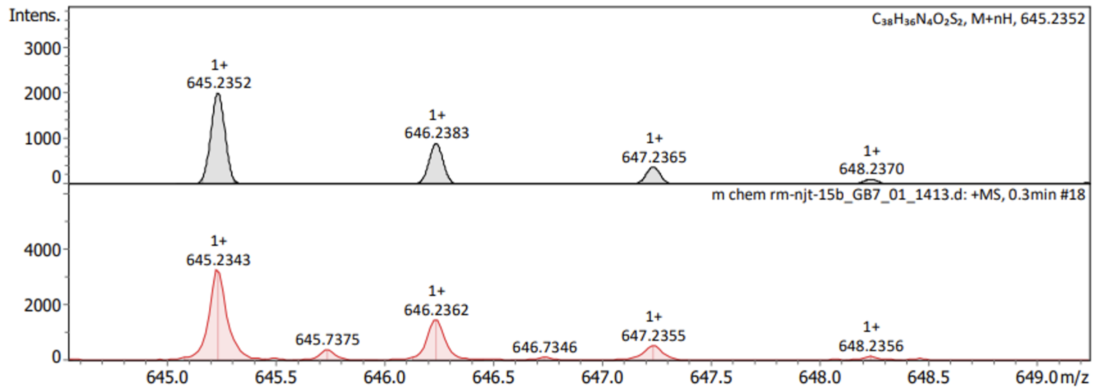

Figure S65 High resolution MS spectrogram of **PTO-DMA**.

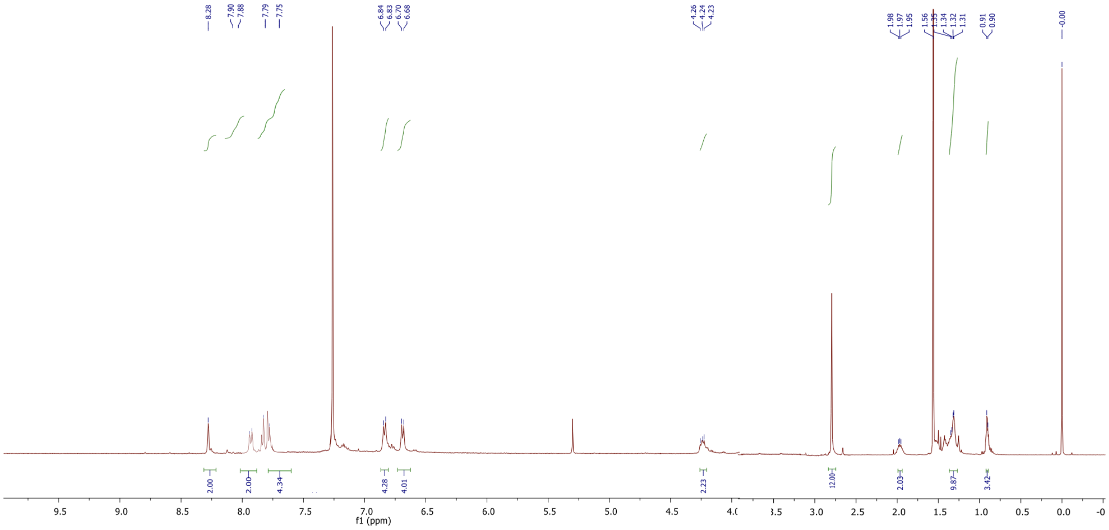

Figure S66 ^{1}H NMR spectrum of **DMA-PTO-DMA**.

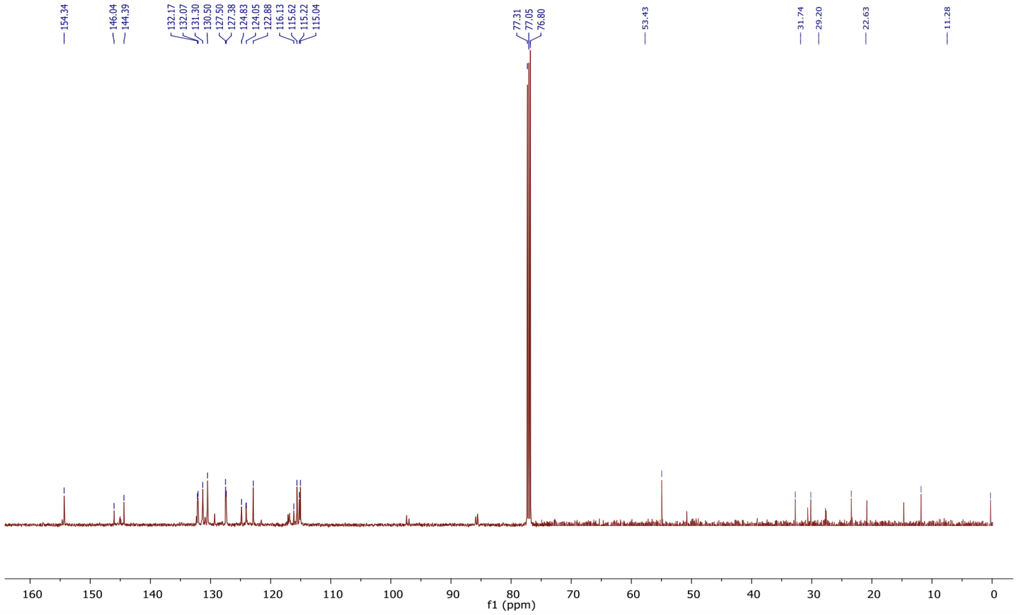

Figure S67 ^{13}C NMR spectrum of **DMA-PTO-DMA**.

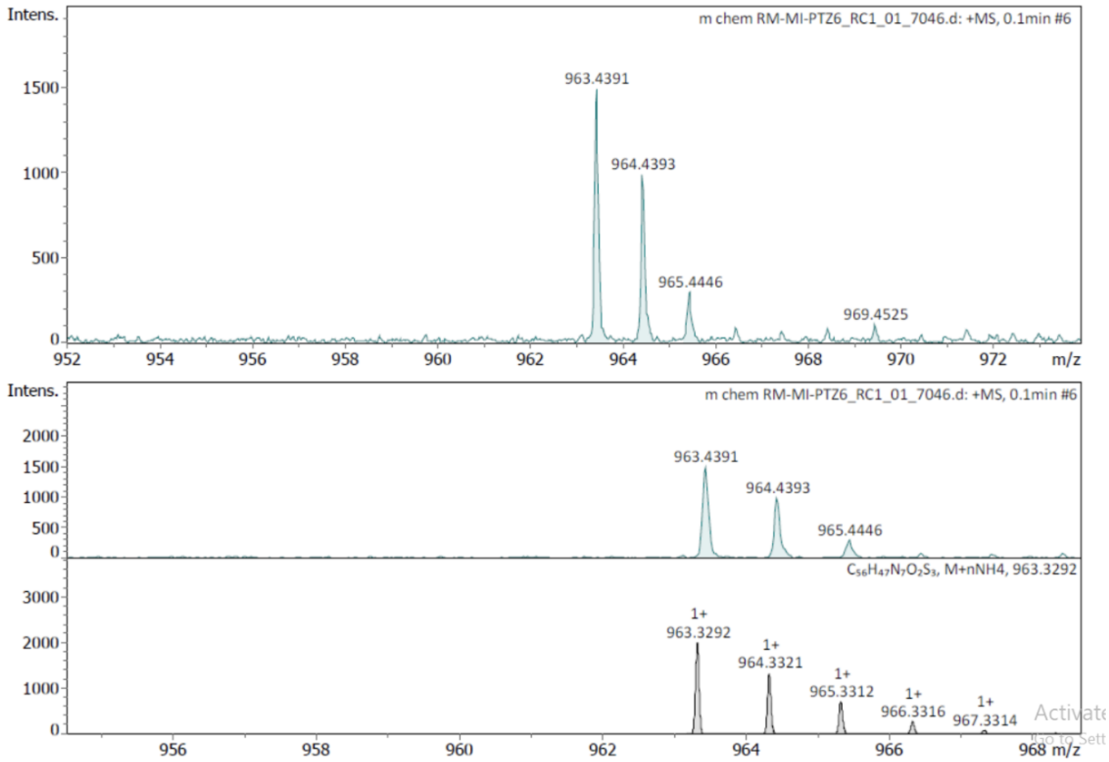

Figure S68 High resolution MS spectrogram of **DMA-PTO-DMA**.

References

[1] L. Mencaroni, B. Carlotti, F. Elisei, A. Marrocchi and A. Spalletti, *Chem. Sci.*, 2022, **13**, 2071–2078.

[2] L. Mencaroni, M. Alebardi, F. Elisei, I. Škorić, A. Spalletti and B. Carlotti, *Phys. Chem. Chem. Phys.*, 2023, **25**, 21089–21099.

[3] M. Koch, R. Letrun and E. Vauthey, *J. Am. Chem. Soc.*, 2014, **136**, 4066–4074.

[4] M. Soederberg, B. Dereka, A. Marrocchi, B. Carlotti and E. Vauthey, *J. Phys. Chem. Lett.*, 2019, **10**, year.

[5] T. Yanai, D. P. Tew and N. C. Handy, *Chem. Phys. Lett.*, 2004, **393**, 51–57.

[6] M. J. Frisch, G. W. Trucks, H. B. Schlegel, G. E. Scuseria, M. A. Robb, J. R. Cheeseman, G. Scalmani, V. Barone, G. A. Petersson, H. Nakatsuji, X. Li, M. Caricato, A. V. Marenich, J. Bloino, B. G. Janesko, R. Gomperts, B. Mennucci, H. P. Hratchian, J. V. Ortiz, A. F. Izmaylov, J. L. Sonnenberg, D. Williams-Young, F. Ding, F. Lipparini, F. Egidi, J. Goings, B. Peng, A. Petrone, T. Henderson, D. Ranasinghe, V. G. Zakrzewski, J. Gao, N. Rega, G. Zheng, W. Liang, M. Hada, M. Ehara, K. Toyota, R. Fukuda, J. Hasegawa, M. Ishida, T. Nakajima, Y. Honda, O. Kitao, H. Nakai, T. Vreven, K. Throssell, J. A. Montgomery Jr., J. E. Peralta, F. Ogliaro, M. J. Bearpark, J. J. Heyd, E. N. Brothers, K. N. Kudin, V. N. Staroverov, T. A. Keith, R. Kobayashi, J. Normand, K. Raghavachari, A. P. Rendell, J. C. Burant, S. S. Iyengar, J. Tomasi, M. Cossi, J. M. Millam, M. Klene, C. Adamo, R. Cammi, J. W. Ochterski, R. L. Martin, K. Morokuma, O. Farkas, J. B. Foresman and D. J. Fox, *Gaussian 16 Rev. B.01*, 2016.

[7] M. J. Abraham, T. Murtola, R. Schulz, S. Páll, J. C. Smith, B. Hess and E. Lindahl, *SoftwareX*, 2015, **1-2**, 19–25.

[8] A. W. Sousa da Silva and W. F. Vranken, *BMC Res. Notes*, 2012, **5**, 367.

[9] J. Wang, R. M. Wolf, J. W. Caldwell, P. A. Kollman and D. A. Case, *J. Comput. Chem.*, 2004, **25**, 1157–1174.

[10] L. E. Chirlian and M. M. Francl, *J. Comput. Chem.*, 1987, **8**, 894–905.

[11] D. van der Spoel, M. M. Ghahremanpour and J. A. Lemkul, *J. Phys. Chem. A*, 2018, **122**, 8982–8988.

[12] T. Darden, D. York and L. Pedersen, *J. Chem. Phys.*, 1993, **98**, 10089–10092.

[13] B. Hess, H. Bekker, H. J. C. Berendsen and J. G. E. M. Fraaije, *J. Comput. Chem.*, 1997, **18**, 1463–1472.

[14] W. G. Hoover, *Phys. Rev. A*, 1985, **31**, 1695–1697.

[15] M. Bernetti and G. Bussi, *J. Chem. Phys.*, 2020, **153**, 114107.
